# Prune-1 drives polarization of tumor-associated macrophages (TAMs) within the lung metastatic niche in triple-negative breast cancer

**DOI:** 10.1016/j.isci.2020.101938

**Published:** 2020-12-13

**Authors:** Veronica Ferrucci, Fatemeh Asadzadeh, Francesca Collina, Roberto Siciliano, Angelo Boccia, Laura Marrone, Daniela Spano, Marianeve Carotenuto, Cristina Maria Chiarolla, Daniela De Martino, Gennaro De Vita, Alessandra Macrì, Luisa Dassi, Jonathan Vandenbussche, Natascia Marino, Monica Cantile, Giovanni Paolella, Francesco D'Andrea, Maurizio di Bonito, Kris Gevaert, Massimo Zollo

**Affiliations:** 1 CEINGE, Biotecnologie Avanzate, Naples 80145, Italy; 2 Dipartimento di Medicina Molecolare e Biotecnologie Mediche (DMMBM), ‘Federico II’ University of Naples, Naples 80134, Italy; 3 European School of Molecular Medicine (SEMM), University of Milan, Milan, Italy; 4 Pathology Unit, Istituto Nazionale Tumori-IRCS- Fondazione G.Pascale, Naples 80131, Italy; 5 VIB-UGent Centre for Medical Biotechnology, Ghent 9052, Belgium; 6 Department of Medicine, Indiana University-Purdue University Indianapolis, Indianapolis 46202, USA; 7 Dipartimento di Sanità pubblica – AOU, Università; degli Studi di Napoli Federico II, Naples 80131, Italy; 8 DAI Medicina di Laboratorio e Trasfusionale, AOU Federico II, Naples 80131, Italy; 9 Department of Biomolecular Medicine, Ghent University, B9052 Ghent, Belgium

**Keywords:** Molecular Biology, Immunology, Cancer

## Abstract

M2-tumor-associated macrophages (M2-TAMs) in the tumor microenvironment represent a prognostic indicator for poor outcome in triple-negative breast cancer (TNBC).

Here we show that Prune-1 overexpression in human TNBC patients has positive correlation to lung metastasis and infiltrating M2-TAMs. Thus, we demonstrate that Prune-1 promotes lung metastasis in a genetically engineered mouse model of metastatic TNBC augmenting M2-polarization of TAMs within the tumor microenvironment. Thus, this occurs through TGF-β enhancement, IL-17F secretion, and extracellular vesicle protein content modulation.

We also find murine inactivating gene variants in human TNBC patient cohorts that are involved in activation of the innate immune response, cell adhesion, apoptotic pathways, and DNA repair. Altogether, we indicate that the overexpression of Prune-1, IL-10, COL4A1, ILR1, and PDGFB, together with inactivating mutations of PDE9A, CD244, Sirpb1b, SV140, Iqca1, and PIP5K1B genes, might represent a route of metastatic lung dissemination that need future prognostic validations.

## Highlights

• Prune-1 correlates to M2-TAMs confirming lung metastatic dissemination in GEMM
• Cytokines and EV proteins are responsible of M2-TAMs polarization processes
• A small molecule with immunomodulatory properties ameliorates metastatic dissemination
• Identification of gene variants within immune response and cell adhesion in TNBC

## Graphical abstract

**Figure:**
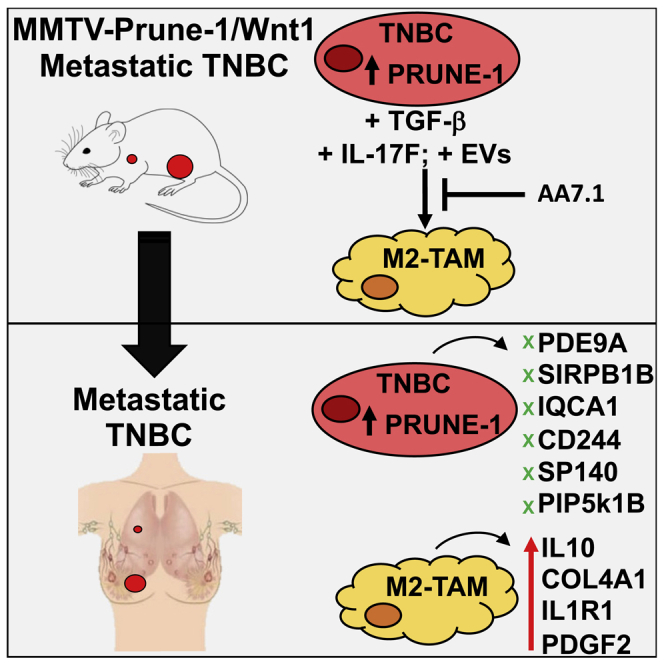

## Introduction

Triple-negative breast cancer (TNBC) accounts for 20% of breast cancers (BCs) (Neophytou et al., 2018), where the tumorigenic cells are negative for the estrogen receptor (ER), progesterone receptor (PgR), and human epidermal growth factor receptor 2 (HER2; i.e., ER^–^, PgR^–^, HER2^–^) (Rakha and Chan, 2011). TNBC is the most aggressive subtype of BCs due to its aggressive clinicopathological features, including young age at onset, large tumor size (Rakha and Chan, 2011), and greater propensity for visceral metastasis to distant sites (Lin et al., 2012). Among the metastatic patients diagnosed with TNBC, 49.3% develop metastasis in the lung (Xiao et al., 2018). Due to the absence of recognized molecular targets for therapy, TNBC patients with lung metastasis have the poorest outcome compared with those diagnosed with other metastatic BC subtypes (Xiao et al., 2018; Cancer Genome Atlas, 2012).

Due to its molecular features, TNBC can be considered one of the most complex tumor disorders in humans. Thus, the application of “-*omics*” technologies is of importance to monitor the genomic evolution of TNBC, which shows dominant TP53, PIK3CA, and PTEN somatic mutations (Shah et al., 2012). Indeed, six distinct molecular TNBC entities have been described: two basal-like-related subgroups (basal-like 1 [BL1] and 2 [BL2]), two mesenchymal-related subgroups (mesenchymal [M], mesenchymal stem-like [MSL]), one luminal androgen receptor (LAR) group, and one immunomodulatory (IM) subgroup, with MSL and IM subtypes that are driven by tumor-associated stromal cells and tumor-infiltrating lymphocytes (TILs), respectively, in the tumor microenvironment (TME) (Lehmann et al., 2011, 2016).

Thus, the immune cells that infiltrate the TME have a dual function in tumor development and metastatic progression. In the early stages of tumorigenesis, they identify and eliminate tumorigenic cells because of the expression of tumor-specific antigens (a mechanism known as “tumor immune surveillance”). Later, the tumors undergo immune-editing processes that allow the tumor variants with reduced immunogenicity to escape immunological detection and elimination, thus allowing tumor variants to be clonally selected (Spano and Zollo, 2012).

The greater genomic instability and the mutational burden of TNBC result in higher propensity to generate neoantigens (Spano and Zollo, 2012; Bianchini et al., 2016), thus generating selected tumorigenic clones within the TME that considerably influence the risk of response to chemotherapy and relapse in these patients (Dieci et al., 2014). Recently, new distinct TNBC subtypes were defined based on the types of immune infiltrating cells within the TME: i.e., the pro-tumorigenic M2-polarized tumor-associated macrophages (M2-TAMs) and the immunosuppressive regulatory T cells (Tregs) (Adams et al., 2017). The TNBC subtypes with the poorest prognosis are characterized by higher levels of infiltrating M2-TAMs (CD163^+^) and Tregs (FOXP3^+^) in a low TILs environment, with high levels of the immunosuppressive programmed death ligand-1 (PD-L1) and glycolytic monocarboxylate transporter 4 (MCT4) markers (Adams et al., 2017). Thus, clinical evidence indicates that M2-TAMs infiltrating tumor tissues act as a prognostic indicator for poor outcome for patients with TNBC, as they are correlated with higher risk for developing metastasis (Sousa et al., 2015; Yuan et al., 2014), as M2-TAMs exert their immunosuppressive functions through inhibition of the effector functions of TILs (Santoni et al., 2017).

It is worth noting the valuable functions of cytokines withing the TME, which are soluble factors that mediate the communication between tumorigenic and immune cells. Transforming growth factor β (TGF-β) is a master regulator of an immunosuppressive action, and it is produced by both tumorigenic and immune cells and can thus orchestrate the polarization processes of the immune system toward a pro-tumorigenic phenotype and promote cancer progression and metastasis, also in TNBC (Pickup et al., 2013). In addition, extracellular vesicles (EVs; including exosomes) generated by tumor cells can modulate the recruitment of the immune cells and their immunomodulation in the TME (Thery et al., 2002), with a crucial role in promoting organotropic pre-metastatic niche formation in TNBC (Hoshino et al., 2015; Chow et al., 2014).

Here, we focus on Prune-1 protein, which belongs to the phosphodiesterase DHH (Asp-His-His) family (D'Angelo et al., 2004) with exopolyphosphatase activity (Tammenkoski et al., 2008). Via its unfolded C-terminus domain, Prune-1 can interact with intracellular binding partners, including glycogen synthase kinase 3β (GSK-3β (Kobayashi et al., 2006)) and NDPK-A (NM23-H1 or NME1 (D'Angelo et al., 2004)). Through these interactors (i.e., GSK-3β, NDPK-A), Prune-1 modulates intracellular pathways that are in turn translated into extracellular signaling, which includes the canonical Wnt1 (Carotenuto et al., 2014) and TGF-β (Ferrucci et al., 2018) cascades. Prune-1 expression levels and metastatic progression have been described in different solid tumors (Oue et al., 2007; Noguchi et al., 2009; Carotenuto et al., 2014; Nambu et al., 2016; Hashimoto et al., 2016; Ferrucci et al., 2018), including metastatic BC (D'Angelo et al., 2004; Zollo et al., 2005). In this regard, the overexpression of Prune-1 has been correlated with advanced nodal status (*i.e.*, N2-N3 cases) and distant metastasis in BC (Zollo et al., 2005).

Our findings here show that Prune-1 is overexpressed in TNBC patients and positively correlates to lung metastasis, tumor grading, disease progression, and infiltrating M2-TAMs. Through intracellular and extracellular signalings in TNBC cells, Prune-1 takes part in the cross-talk with macrophages, thus enhancing their polarization status toward the M2 phenotype. This action is synergistically mediated by a dual extracellular mode of communication: (i) via enhancement of secretion of soluble cytokines (i.e., interleukin-17F [IL-17F] (Yang et al., 2008; Giles et al., 2016; Starnes et al., 2001), IL-28 (Zhou et al., 2007), IL-20 (Otkjaer et al., 2007)); (ii) using EVs to carry proteins (i.e., Vim (Yamashita et al., 2013), Sdcbp (Yang et al., 2013b; Qian et al., 2013; Koo et al., 2002), Ifitm3 (Yang et al., 2013a)) that are responsible for metastatic process. Furthermore, the overexpression of Prune-1 (together with Wnt1) in the mammary gland of a genetically engineered mouse model (GEMM) of metastatic TNBC (mouse mammary tumor virus-[MMTV]–Prune-1/Wnt1) drives genetic variants involved in activation of the innate immune response (leukocytes and macrophage activation; ANKHD1, FER1L5), cell adhesion (NEXN), apoptotic pathways (BID), and DNA repair (ERCC5). Genetic inactivating mutations in the PDE9A, CD244, Sirpb1b, SV140, Iqca1, and PIP5K1B genes were also found in human BC specimens.

Through this approach we have identified in TNBC a common mutation burden in tumorigenic cells and a related network of action on immunoregulation of the immunosystem, with a focus on macrophages.

## Results

### Overexpression of Prune-1 in metastatic TNBC cells enhances the canonical TGF-β and Wnt signaling cascades

Through interactions with its protein-binding partners (i.e., NDPK-A (Garzia et al., 2008; D'Angelo et al., 2004), GSK-3β (Kobayashi et al., 2006)), Prune-1 modulates signaling cascades, including the canonical Wnt (Carotenuto et al., 2014) and TGF-β pathways (Ferrucci et al.), which are also involved in BC progression and metastasis (Pohl et al., 2017; Ding et al., 2016).

**Figure 1:**
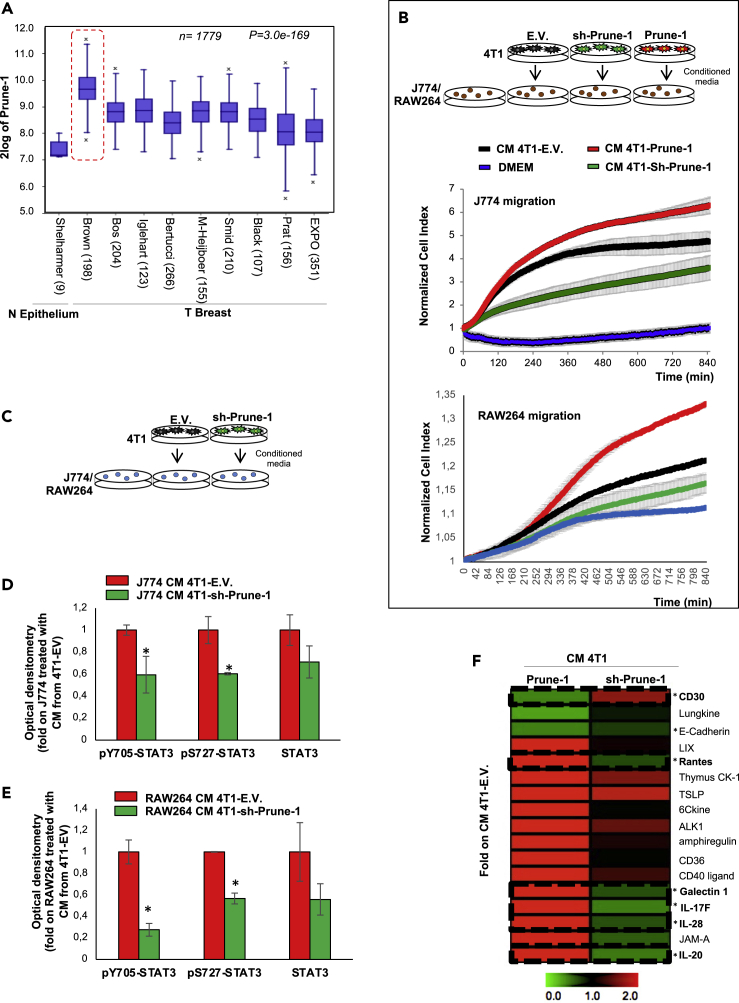
Prune-1 protein is overexpressed in TNBC and promotes macrophage chemotaxis through STAT3 via soluble cytokines (A) Prune-1 overexpression in TNBC. RNA log2 expression analysis of Prune-1 levels of primary BC samples across different publicly available datasets, compared with normal epithelium (N Epithelium; Shelharmer dataset only). Data from 10 independent public-domain BC gene-expression datasets show the overexpression of Prune-1 in all of the BC samples compared with normal epithelium. Higher Prune-1 expression levels are seen for TNBC samples (i.e., Brown (Burstein et al., 2015); red dashed line) (n = 1779; p = 3.0 × 10^−169^). (B) Overexpression of Prune-1 in TNBC cells enhances macrophage chemotaxis *in vitro*. Normalized Cell Index as a measure of cell migration/chemotaxis of J774A.1 (i.e., J774; **upper panel)** and RAW264.7 (i.e., RAW264; **lower panel**) macrophages as generated by the xCELLigence RTCA software. Migration kinetics were monitored in response to conditioned media from Prune-1-overexpressing (4T1–Prune-1, red), Prune-1-silenced (4T1–Sh-Prune-1, green), and empty vector 4T1 cell clones (black), used as chemoattractants. Dulbecco's modified Eagle's medium was used as the negative control (blue). (C–E) Conditioned media (CM) from the 4T1 clones were collected after 24 h. J774 or Raw264 macrophages were starved for 6 h in serum-free medium and then grown in the conditioned media for 30 min (C). Densitometer analyses of immunoblotting for the indicated proteins in J774 (D) and Raw264 (E) macrophages grown for 30 min in conditioned media from Prune-1-silenced and control 4T1 clones are shown. Empty vector (EV) 4T1 clones and untreated (UNT) macrophages were used as the negative controls. β-Actin levels were used as the loading control. ∗p < 0.05 in Student's t test compared with J774 (D) or Raw264 (E) treated with conditioned media from 4T1 EV control clones. (F) Prune-1 induces the secretion of soluble proteins by TNBC cells. Densitometer analyses of the cytokines upregulated and downregulated in the conditioned media (CM) derived from Prune-1-overexpressing (4T1–Prune-1) and Prune-1-silenced (4T1–Sh-Prune-1) 4T1 clones (MultiExperiment Viewer, http://www.tm4.org/mev.html). Among the 17 cytokines modulated by Prune-1 in the conditioned media collected from Prune-1-overexpressing (4T1–Prune-1), one was upregulated (CD30) and five were downregulated (Rantes, Galectin-1, IL-17F, IL-28, IL-20) in the conditioned media from 4T1–Sh-Prune-1 cell clones, thus following an opposite trend. ∗p < 0.05 in Student's t test comparing cytokines levels in conditioned media of 4T1–Sh-Prune-1 cells with those in 4T1 Empty Vector control clones.

Here, through applying data mining approaches in public gene expression resources available in BC datasets (R2 Genomics Analysis and Visualisation Platform; http://r2.amc.nl), we found higher Prune-1 expression in all BC samples (n = 1779, p = 3.0 × 10^−169^; Figure 1A), thus confirming Prune-1 overexpression in BC. Interestingly, we found the highest expression of Prune-1 in the public TNBC dataset (i.e., Brown (Burstein et al., 2015), n = 198; Figures 1A and S1A, within the red dashed line), which thus suggested a role for Prune-1 in this highly metastatic BC subgroup. These data are not surprising, because of the major frequency of chromosome 1q21 gain in basal-like and TNBC (30%–40% (Silva et al., 2015), (Cancer Genome Atlas, 2012)) and also in those with recurrent BC (70% (Goh et al., 2017)).

Triple-negative breast cancer is known to correlate to negative expression of ERs and PgR status (i.e., ER^–^/PgR^–^/HER2^–^) (Bianchini et al., 2016). To provide deeper insight into the role of Prune-1 in TNBC, its association with ER, PgR, and HER2 status was also investigated using the public accessible dataset of tumor breast invasive carcinoma, with gene expression data acquired for the BC cohort from The Cancer Genome Atlas (TCGA; n = 1,097). In this dataset, BC samples were stratified according to their ER, PgR, and HER2 scores (as evaluated by immunohistochemistry [IHC]), which ranged from 0 to 3^+^, with 0 indicating negativity. Higher expression levels of Prune-1 were identified only in those samples with negative scores for both ER and PgR (i.e., 0), and in those with scores ranging from 0 to 1^+^ for HER2 status (Figure S1B, within the red dashed lines). These data prompted us to suggest potential involvement of Prune-1 in the pathogenesis of TNBC.

We have previously defined Prune-1 as an inducer of the SMAD-mediated canonical TGF-β pathway in metastatic medulloblastoma group 3 (MB_group3_) (Ferrucci et al., 2018). As the TGF-β cascade has a crucial role in tumor metastasis, we investigated the expression levels of its downstream effectors (i.e., SMAD2, SMAD4) in BCs using different public BC datasets. These analyses showed that both SMAD2 and SMAD4 expression levels were higher in the TNBC dataset (i.e., Brown (Burstein et al., 2015); as shown in Figure S1C; SMAD2: p = 5.6 × 10^−230^; SMAD4: p = 2.1 × 10^−86^), thus indicating correlation between Prune-1 and canonical TGF-β signaling effectors in TNBC (Drabsch and Ten Dijke, 2012). Further correlation analyses were performed between Prune-1, SMAD2, and SMAD4 in BC samples stratified according to their ERs and PgR status, using additional gene-expression data acquired from the publicly accessible cohort of BC samples in TCGA (n = 1,097; Table S1). This analysis showed that Prune-1 positively correlated with both SMAD2 and SMAD4 levels in the BC samples with PgR, ER, and HER2 negative status (R value, 0.19–0.35; Table S1). Overall, Prune-1 positively correlated with the TGF-β downstream effectors in TNBC. This is of great interest for the immunoregulatory action of TGF-β exerted in the TME.

Using a murine model of metastatic TNBC cells (i.e., 4T1 cells (Yoneda et al., 2000)), independent stable clones were generated: Prune-1-silenced (0.6-fold, p = 0.02; Figure S2A) and h-Prune-1-FLAG-overexpressing (Figure S2B) 4T1 clones. We asked whether any Prune-1-induced perturbation can modulate TGF-β signaling pathways. As shown in Figure S2C, decreased levels of phosphorylated-(Ser467)-SMAD2 (i.e., phospho-SMAD2, the main effector of the canonical TGF-β cascade) were shown in Prune-1-silenced 4T1 clones. In contrast, increased phospho-SMAD2 levels were seen in the Prune-1-overexpressing 4T1 clones (Figure S2D), when compared with the control clones (i.e., empty vector, E.V.). Overall, these data confirmed that Prune-1 enhances the canonical (SMAD2-mediated) TGF-β pathway also in TNBC cells (Ferrucci et al., 2018).

As previous reports had shown that through binding to GSK-3β (Kobayashi et al., 2006), Prune-1 can enhance canonical Wnt signaling (Carotenuto et al., 2014), we also investigated the activated β-catenin protein levels in these 4T1 stable clones using western blotting (Figures S2C and S2D). These data showed reduced protein levels of activated β-catenin (i.e., dephosphorylated on Ser37 and/or Thr41) in Prune-1-silenced 4T1 clones (Figure S2C), whereas increased levels were observed in the Prune-1-overexpressing cells (Figure S2D). Overall, these results also confirm that Prune-1 can positively modulate the canonical Wnt signaling cascade in these TNBC cells, as previously shown for lung cancer (Carotenuto et al., 2014).

In summary, Prune-1 enhances the activation of canonical TGF-β and Wnt signaling pathways *in vitro* in a metastatic model of TNBC (i.e., 4T1 cells).

### Prune-1 at the interplay of communication between TNBC cells and macrophages

Tumorigenic and immune cells within the TME communicate through extracellular mediators (e.g., cytokines, EVs, exosomes), which are also sensors in the modulation of immune cells (Spano and Zollo, 2012). We previously reported that Prune-1 has an extracellular role in paracrine communication via Wnt3a cytokine secretion (Carotenuto et al., 2014). As clinical evidence indicates that M2-TAMs positively correlate with metastasis and poor outcome in TNBC (Sousa et al., 2015; Yuan et al., 2014), we here determined the potential involvement of Prune-1 in the intratumoral recruitment of M2-TAMs in TNBC.

In this regard, we evaluated the recruitment/migration of immune cells using murine J774A.1 (#ATCC-TIB-67 (Lam et al., 2009)) and Raw264.7 (ATCC-TIB-71) macrophages, by performing a real-time cell motility assays (Cell Index) in which conditioned media from 4T1-Prune-1 cell clones (as previously described) were used as chemoattractants. As shown in Figure 1B, the conditioned media from Prune-1-overexpressing 4T1 cell clones increased the migration rates of both J774A.1 and Raw264.7 macrophages (Figure 1B, red lines), whereas the media from Prune-1-silenced 4T1 cell clones reduced their migration rate (Figure 1B, green lines). These results were compared with those from EV control clones (Figure 1B, black lines).

Furthermore, the conditioned media from 4T1 cell clones were also used to grow macrophages *in vitro*, to determine the activation status of macrophages by measuring the phosphorylation status of the STAT3 protein (i.e., pY705-STAT3 is required for dimerization and nuclear translocation (Levy and Darnell, 2002), whereas pS727-STAT3 is linked to increased STAT3 transactivation (Decker and Kovarik, 2000)), due to its prominent role in macrophage activation and during M1-M2 switch in the polarization processe (Wang et al., 2014). As shown in Figures 1C–1E, S2E, and S2F, pY705-STAT3 and pS727-STAT3 were significantly decreased in the J774A.1 (pY705-STAT3: 0.6-fold, p = 0.05; pS727-STAT3: 0.6-fold, p = 0.04; Figures 1D and S2E) and Raw264.7 (pY705-STAT3: 0.27-fold, p = 0.01; pS727-STAT3: 0.56-fold, p = 0.006; Figures 1E and S2F) macrophages cultured in media from Prune-1-silenced 4T1 clones, compared with those cultured in media from the control clones (EV). In contrast, we did not find any significant downregulation of total STAT3 in both J774A.1 (0.7-fold, p = 0.17, Figures 1D and S2E) and Raw264.7 (0.55-fold, p = 0.18, Figures 1E and S2F) macrophages. However, the conditioned media collected from the Prune-1-silenced 4T1 cell clone do not completely abolish phosphorylated STAT3 protein levels in the recipient macrophages (Figures 1C–1E, S2E, and S2F). We believe that this is because of the incomplete knockdown of m-Prune-1 in the 4T1 cell clone (0.6-fold downregulation, p = 0.02, see Figure S2A). However, it is not possible to exclude the presence of activating factors of STAT3 that are independent of Prune-1 actions.

For the above reasons, we believe that this finding might represent one of the mechanisms in the downregulation of the STAT3 pathway in these macrophages grown in the conditioned media collected from Prune-1-silenced tumorigenic cells.

### Secretion of inflammatory cytokines from TNBC cells are modulated by Prune-1

We have here investigated whether Prune-1 can influence the recruitment/activation of macrophages by modulation of the secretion of extracellular mediators (i.e., cytokines, chemokines). For this purpose, we compared the levels of 144 cytokines secreted in the conditioned media collected from three different independently generated Prune-1-overexpressing 4T1 cell clones (pooled together) and compared them to those secreted by the EV control clones (Figure S3A, upper panel). These analyses showed 17 cytokines that were differentially secreted (14 upregulated >2-fold; three downregulated <0.5-fold; see fold intensity in Figure S3B). In particular, the levels of CD30, lungkine/CXCL15, and E-cadherin were significantly decreased in the pooled conditioned media from Prune-1-overexpressing 4T1 clones, compared with those from the control clones. In contrast, the levels of LIX/CXCL5, thymus CK-1/CXCL7, thymic stromal lymphopoietin (TSLP), 6Ckine, ALK1, amphiregulin (Areg), CD36, CD40 ligand/CD154, galectin 1, IL-17F, IL-28, IL-20, JAM-A/F11R, and “regulated upon activation, normal T cell expressed and secreted” (RANTES or CCL5) were increased in the pooled conditioned media derived from Prune-1-overexpressing 4T1 clones, compared with conditioned media from the control clones (Figure S3B). To further confirm these data, we determined the levels of the selected Prune-1-modulated cytokines (i.e*.*, n = 17) in the conditioned media from Prune-1-silenced 4T1 cell clones (Figure S3A, bottom panel). These data showed that among these 17 cytokines analyzed, E-cadherin, RANTES, galectin 1, IL-17F, IL-20, and IL-28 (also known as type III interferon-λ) levels were significantly decreased (Figures 1F and S3C). In contrast, CD30 levels increased in the conditioned media collected from Prune-1-silenced 4T1 cells, thus following an opposite trend compared with those in conditioned media from Prune-1-overexpressing 4T1 cell clones (Figure 1F). These findings indicated that secretion of the RANTES, galectin 1, IL-17F, IL-20, and IL-28 cytokines might be positively modulated by Prune-1 expression in TNBC cells. Gene Ontology analyses indicated that these cytokines are involved in immune system process (GO:0002376, fdr:0.0046), immune response (GO:0002376, fdr: 0.0138), immune effector processes (GO:0002252, fdr: 0.0302), and innate immune responses (GO:0045087, fdr: 0.0473), thus suggesting involvement in the regulation of immune cells within the TME. Of importance, among these cytokines, IL-20 and IL-28 were reported to take part to JAK-STAT signaling pathway activation (KEGG: mmu04630, fdr: 0.004), which is involved in M1- or M2-macrophage polarization (Wang et al., 2014). Of interest, through its binding to IL-17RA on recipient endothelial cells, the IL17A/IL17F heterodimer was previously reported to activate the STAT3 pathway (mainly via Y705 phosphorylation) and consequently to recruit immune cells (Yuan et al., 2015).

Altogether, we have described here that overexpression of Prune-1 in 4T1 murine TNBC cells might have a role in modulation of macrophage activation through the extracellular secretion of soluble cytokines (i.e., RANTES, galectin1, IL17-F, IL-20, IL-28). We measured the “activation” of macrophages by evaluating their migration rate and STAT3 pathway (Wang et al., 2014), because its activation in TAMs has been shown to induce immunosuppression, angiogenesis, cell growth, and metastasis (Yu et al., 2007; Dang et al., 2015). Furthermore, STAT3 inhibition was also reported to “re-educate” TAMs via reversing their phenotype from M2 to M1 (Fujiwara et al., 2014; Zhang et al., 2013). How Prune-1 influences the expression of these cytokines in tumorigenic cells and the activation of STAT3 in macrophages will be the focus of other studies.

### Prune-1 modulates TAMs in the TME in a genetically engineered mouse model of metastatic TNBC

Sequence homology analyses between human (h) and murine (m) Prune-1 protein sequences show a significant similarity (overall aa: 85.43% identity; DHH and DHHA2 enzymatic domains: 88.24% and 82.39% identity, respectively; NME1-binding domain (Carotenuto et al., 2013): 83.33% identity; see Figure S4A). To determine whether the overexpression of h-Prune-1 has similar immune responses (in terms of cytokines expression) to m-Prune1, we transiently transfected h-Prune-1 or m-Prune-1 cDNA plasmid constructs into Raw264 macrophages, because of their lower Prune-1 endogenous expression levels compared with J774 macrophages (Figure S4B). Using real-time PCR analysis, we showed the same levels of upregulation of inflammatory cytokines (i.e., IL-10, Arg1, MMP9, IL-1β) in both h-Prune-1- and m-Prune-1-overexpressing Raw264 macrophages (Figures S4C and S4D). These data show the potential involvement of the Prune-1 protein in the modulation of cytokines expression also in immune cells (i.e., macrophages), and they also show no significant differences between the functional regulation of the human versus murine Prune-1 proteins. For these reasons and to characterize the effects of Prune-1 overexpression in the TME of BC, we generated a GEMM-overexpressing human Prune-1 in the mammary gland.

This GEMM was generated using a vector construct that contained h-Prune-1 cDNAs under the control of the MMTV promoter (Callahan and Smith, 2000) (Figure S4E). Female mice harboring h-PRUNE-1 cDNA overexpressed in mammary glands (i.e., MMTV–Prune-1) developed mammary hyperplasia early in life (by 80 days of age), compared with the control FVB mice, as shown in Figure S4F. These MMTV–Prune-1 mice were monitored for 12 months. Although benign mammary lesions that were usually hyperplasia were seen in 100% of the female mice, none developed tumors in the mammary glands over 1 year of observation.

**Figure 2:**
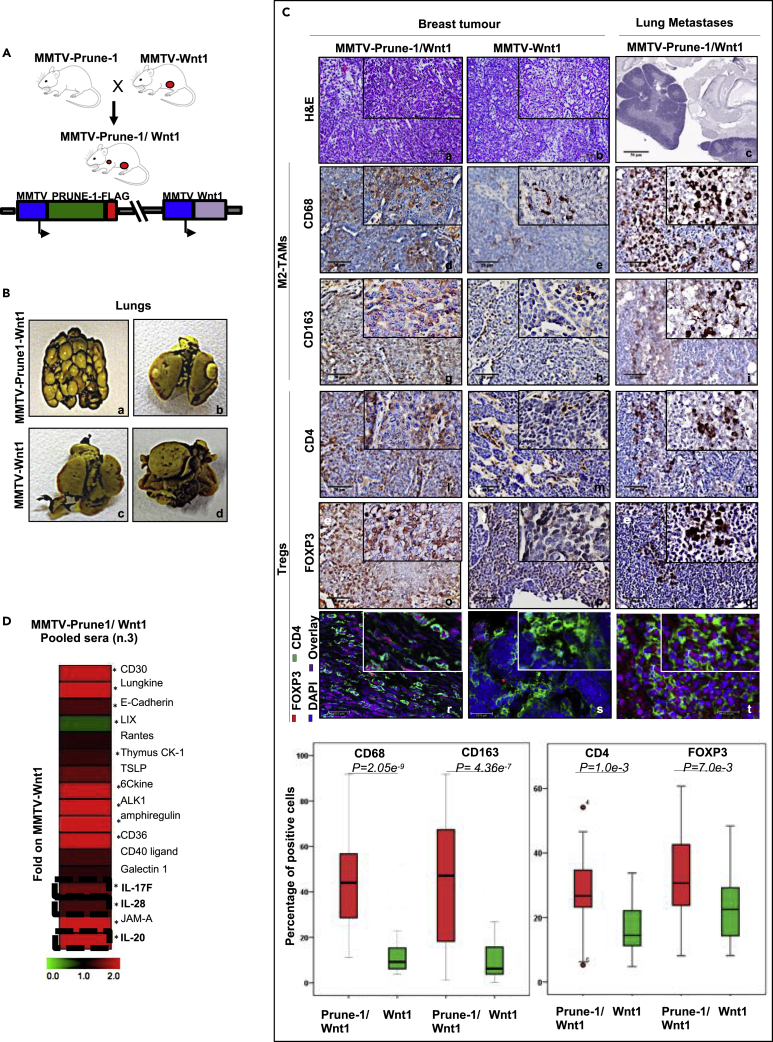
Prune-1 protein overexpressed in metastatic TNBC mouse model (MMTV–Prune-1/Wnt1) promotes M2-TAMs recruitment in both primary tumor and metastatic niche via soluble cytokines (A) Schematic diagram showing the cross between MMTV–Prune-1 and MMTV–Wnt1 mice to obtain the double transgenic MMTV–Prune-1/Wnt1 model. (B) Representative photographs of the lungs from MMTV–Prune-1/Wnt1 **(a–b)** and MMTV–Wnt1 **(c–d)** mice fixed in Bouin's solution. Metastatic foci (i.e., macrometastasis) are visible in the lungs from MMTV–Prune-1/Wnt1 **(a–b)**. (C) Representative hematoxylin-eosin staining **(a–c)**, immunohistochemistry (IHC; **d–q)**, and immunofluorescence (IF; **r–t**) performed on sections of mammary tumors developed from MMTV–Prune-1/Wnt1 and MMTV–Wnt1 mice, and metastatic lungs from MMTV–Prune-1/Wnt1 mice, using antibodies against the following: CD68 **(d–f)** and CD163 **(g–i)**, as markers for M2-TAMs; CD4 **(l–n)**, as a marker for T cells; and FOXP3 **(o–q)**, as a marker for Tregs. Double indirect IF was performed to detect Tregs (i.e., CD4+ FOXP3+, **r–t**). CD4: green; FOXP3: red; DAPI: blue. Quantification was performed using a quantitative pathology workstation (Mantra) with image analysis software (inform). Data were calculated from three independent tumors. Graphs were constructed using the IBM SPSS statistics software. Magnification, 5×, 20×, 40×. Scale bar: 50 μm, hematoxylin-eosin; 20 μm, IHC; 10 μm, IF. (D) Prune-1 induces secretion of soluble cytokines *in vivo*. Densitometer analyses of the cytokines upregulated and downregulated in the sera collected and pooled from three MMTV–Prune-1/Wnt1 mice and MMTV–Wnt1 mice. Among the cytokines modulated by Prune-1, significant upregulation of IL-17F, IL-28, and IL-20 was seen, with an opposite trend compared with the cytokines in the conditioned media from Prune-1-silenced 4T1 cells. Data are means ± standard deviation. Data were represented using MultiExperiment Viewer (http://www.tm4.org/mev.html). ∗, p < 0.05 in Student's t test comparing cytokines levels of pooled sera from n.3 MMTV-Prune-1/Wnt1 mice with those from n.3 MMTV-Wnt1 mice.

For these reasons, female MMTV–Prune-1 mice were then crossed with MMTV–Wnt1 mice (Figures 2A and S4G), a GEMM resembling “basal-like” TNBC (Li et al., 2000; Yu et al., 2016; Herschkowitz et al., 2007), which rarely develops spontaneous metastasis to the lungs (Huang et al., 2008). The resulting double transgenic MMTV–Prune-1/Wnt1 mouse model (n = 31) developed mammary tumors (with 100% penetrance), as did the MMTV–Wnt1 mice (n = 44). Interestingly, the overexpression of both Prune-1 and Wnt1 in the mammary glands did not alter mammary tumor onset, compared with the MMTV–Wnt1 mice (Figure S4H). Furthermore, through IHC analysis, we confirmed that the mammary tumors that were generated in the double transgenic MMTV–Prune-1/Wnt1 mice resemble the TNBC subgroup with undetectable levels of both ERs and PgRs (Figure S5A), thus confirming these models as GEMM of TNBC. Nevertheless, although MMTV–Wnt1 mice did not develop lung metastasis at 2 months from tumor onset, MMTV–Prune-1/Wnt1 mice showed macro-metastasis in the lungs at 97% penetrance (Figure 2B, Table S2).

In addition, we investigated the presence of immune infiltrating cells in the mammary TME. Among the immune infiltrating cells, we focused on M2-TAMs (CD163^+^) and Tregs (FOXP3^+^), which have been reported as markers for the subtype of TNBC with poorest prognosis (Adams et al., 2017). As shown in Figure 2C, our quantitative IHC and immunofluorescence (IF) analyses (using a quantitative pathology approach; see Methods) revealed increased levels of both M2-TAMs (i.e., CD68^+^ CD163^+^ cells) and Tregs (i.e., FOXP3^+^ CD4^+^ cells) in the TME of MMTV–Prune-1/Wnt1 mice compared with MMTV–Wnt1 (CD68: p = 2.05 × 10^−9^; CD162: p = 4.36 × 10^−7^; CD4: p = 1.0 × 10^−3^; FOXP3: p = 7.0 × 10^−3^). Of note, M2-TAMs and Tregs were also found in the metastatic niche into the lungs of the MMTV–Prune-1/Wnt1 mice (Figure 2C). Of interest, we performed the same IHC and IF analyses on the contralateral nontumoral mammary glands of MMTV–Prune-1/Wnt1 and MMTV–Wnt1 mice, and on the lungs from MMTV–Wnt1 mice (Figure S5B), where immune infiltrating cells were not found. Altogether these data indicate a role for Prune-1 in combination with other factors in the recruitment/activation of immunosuppressive cells in the TME of both the primary tumor and lung metastatic microenvironments in TNBC.

To better underpin the role of Prune-1 in metastatic TNBC, we also investigated the function of Prune-1 in the activation of TGF-β signaling and induction of epithelial-mesenchymal transition (EMT) (Ferrucci et al., 2018). Here, higher SMAD2/3 levels were detected in primary mammary tumors derived from the double transgenic mice (i.e., MMTV–Prune-1/Wnt1), compared with those from MMTV–Wnt1 models (Figures S6A and S6B). Furthermore, undetectable E-cadherin and higher N-cadherin levels were found in the tumors from MMTV–Prune-1/Wnt1 mice compared with those from MMTV–Wnt1 mice, which in contrast showed higher E-cadherin and lower N-cadherin levels (Figures S6A and S6B). Of interest, the microenvironment of the nontumoral contralateral mammary gland also shows higher SMAD2/3 and N-cadherin and lower E-cadherin levels in MMTV–Prune-1/Wnt1 compared with those from MMTV–Wnt1 models (Figures S6C and S6D). These data thus confirm that Prune-1 enhances TGF-β and EMT also in this GEMM of metastatic TNBC.

Furthermore, of interest, there were increased levels of IL-17F, IL-20, and IL-28 in the sera from MMTV–Prune-1/Wnt1 mice (as pooled sera from three mice) compared with those from MMTV–Wnt1, as shown in Figures 2D, S7A, and S7B. These cytokines were previously shown to be overexpressed in conditioned media collected from Prune-1-overexpressing 4T1 cells and downregulated in those derived from Prune-1-silenced 4T1 clones (Figure 1F), thus translating the *in-vitro* findings into the *in-vivo* results.

Thus, as overexpressed in this GEMM of metastatic TNBC, Prune-1 can modulate immunosuppressive cells (including M2-TAMs) in the TME also through the secretion of these extracellular soluble mediators (i.e., IL-17F, IL-20, IL-28).

### Prune-1 activates metastatic pathways and enhances the migratory phenotype in murine TNBC primary cells

To dissect out the function of Prune-1 in TNBC, primary murine tumorigenic cells were obtained from the tumors generated from MMTV–Prune-1/Wnt1 and MMTV–Wnt1 mice (at 2 months from the tumor onset; Figures S8A and S8B). Here we show the activation of both canonical TGF-β and Wnt signaling in MMTV–Prune-1/Wnt1 cells compared with MMTV–Wnt1 cells, as shown by increased levels of phospho-Ser467-SMAD2, phospho-Ser9/21-GSK-3β, and Wnt3a (Figure S8C). Importantly, the same analysis also showed increased levels of EMT markers in MMTV–Prune-1/Wnt1 cells (i.e., undetectable E-cadherin, higher N-cadherin levels), increased phosphorylation levels of phospho-(Ser-473)-AKT, and decreased expression of its repressor PTEN, compared with MMTV-Wnt1 cells (Figure S8C). These data also showed increased levels of phosphorylated Ser120-122-125-NME1 (or NDPK-A; sign of complex formation with the Prune-1 protein) in these primary cells (Garzia et al., 2008) (Figure S8C). Altogether, these results indicate the activation of Prune-1–metastatic pathway (as Prune-1 in complex formation with NME1; as previously described for medulloblastoma (Ferrucci et al., 2018)) also in these murine TNBC primary cells (i.e., MMTV–Prune-1/Wnt1 cells), thus overall suggesting increased migratory properties of these primary Prune-1-overexpressing TNBC cells.

These findings were further supported *in vitro* by assays performed using real-time proliferation and migration assays. These data showed that the primary MMTV–Prune-1/Wnt1 cells have higher proliferative index (Figure S8D), shorter doubling time (i.e., MMTV–Prune-1/Wnt1: 28.4 h; MMTV–Wnt1: 35.8 h; p = 4.1 × 10^−5^; Figure S8E) and greater migration rate (using 10% FBS as chemoattractant, Figure S8F) when compared with MMTV–Wnt1 cells.

Overall these data indicate that Prune-1 can enhance the proliferative and migratory properties also in these primary tumorigenic cells derived from mammary tumors generated from our GEMM.

### Macrophage polarization is enhanced by Prune-1 overexpression in TNBC

**Figure 3:**
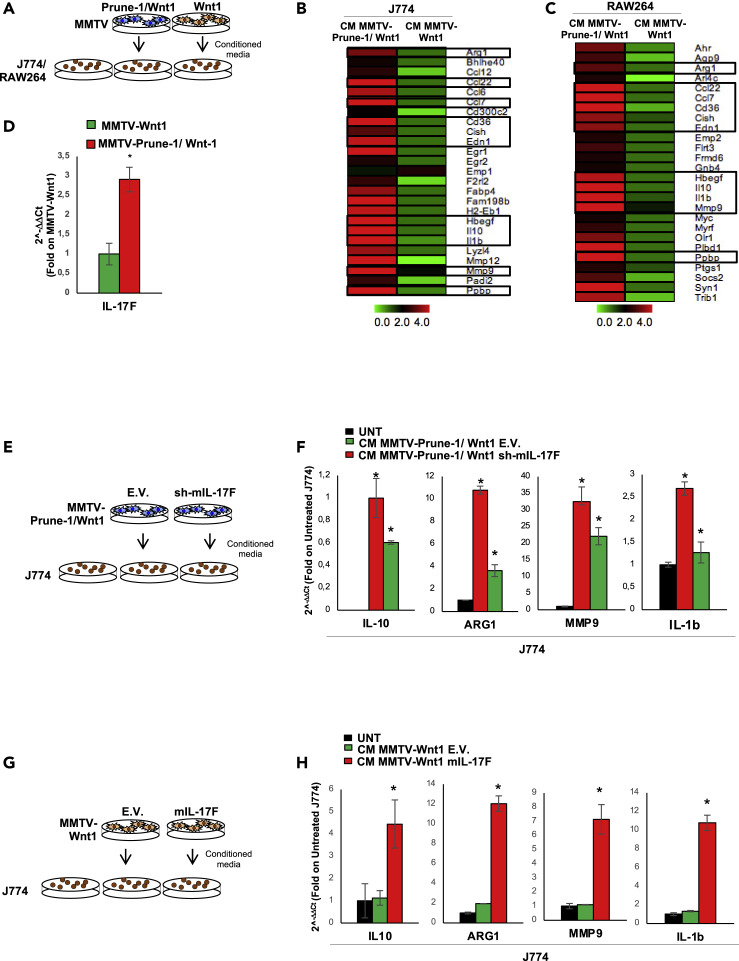
Prune-1 overexpressed in TNBC cells promotes M2-polarization of macrophages *in vitro* through IL-17F (A–C) J774A.1 and Raw264 macrophages were grown for 48 h in conditioned media collected (after 24 h) from MMTV–Prune-1/Wnt1 or MMTV–Wnt1 cells (A). Fold-change heatmap (http://www.tm4.org/mev.html) for upregulated M2-associated genes (fold change on untreated macrophages >2, p < 0.01) in J774A.1 (B) and Raw264 (C) macrophages grown in conditioned media from MMTV–Prune-1/Wnt1 versus MMTV–Wnt1 cells. The black boxes indicate the common genes upregulated in both J774A.1 and Raw264 macrophages. The color scale under the heatmap illustrates the fold-change values shown in the heatmap. (D) Real-time PCR analysis of IL-17F in MMTV–Prune-1/Wnt1 (red) and MMTV–Wnt1 (green) cells. ∗p < 0.05 in Student's t test comparing IL-17F levels of MMTV-Prune-1/Wnt1 with MMTV-Wnt1 cells. (E and F) J774A.1 macrophages were grown for 48 h in conditioned media collected (after 24 h) from MMTV–Prune-1/Wnt1 previously transfected with murine sh-IL17F plasmid or empty vector (EV) as negative control (E). Real-time PCR analysis of some M2-associated genes, including IL-10, Arg-1, MMP-9, and IL-1β, in J774A.1 macrophages grown for 48 h in conditioned media from MMTV–Wnt1 cells transfected with sh-IL-17F (red) or EV control (green) (F). Untreated J774A.1 macrophages were used as the negative controls (UNT, black). ∗p < 0.05 in Student's t test compared with untreated macrophages. (G and H) J774A.1 macrophages were grown for 48 h in conditioned media collected (after 24 h) from MMTV–Wnt1 previously transfected with murine IL-17F or EV as negative control (G). Real-time PCR analysis of some M2-associated genes, including Arg-1, MMP-9, and IL-1β, in J774A.1 macrophages grown for 48 h in conditioned media from MMTV–Wnt1 cells transfected with IL-17F (red) or EV control (green). Untreated J774A.1 macrophages were used as the negative controls (UNT, black) (H). ∗p < 0.05 in Student's t test compared with untreated macrophages.

Whether Prune-1 has a role in the polarization process of macrophages in these primary murine TNBC cells was also investigated. For this purpose, conditioned media from MMTV–Prune-1/Wnt1 and MMTV–Wnt1 primary cells (collected over 24 h) were used to grow J774A.1 and Raw264 macrophages *in vitro* (Figure 3A), to evaluate their “polarization status” by measuring the expression levels of “M2-associated genes” (Orecchioni et al., 2019) through whole-genome RNA sequencing approaches (i.e., RNAseq; Figures 3B and 3C; see Additional Data). Untreated macrophages were used as the negative control. These data showed upregulation (i.e., fold change on untreated macrophages >2, p < 0.01) of 24 genes (Figure 3B, Table S3) and 27 genes (Figure 3C, Table S3) belonging to “M2-associated genes” in the J774A.1 and Raw264 macrophages, respectively, grown in the conditioned media collected from MMTV–Prune-1/Wnt1 cells, as compared with those grown in MMTV–Wnt1-derived media. In contrast, only one gene (i.e., Emp1) was upregulated (i.e., fold change over untreated macrophages >2; p < 0.01) in J774A.1 cells treated with conditioned media from MMTV–Wnt1 cells (Figure 3B). This thus indicated that Prune-1 takes part in the polarization processes. Of interest, among those upregulated genes in the macrophages grown in the conditioned media from MMTV–Prune-1/Wnt1 cells, 11 were common between J774A.1 and Raw264 macrophages (Figures 3B and 3C, black boxes). These included the most representative M2-associated genes (i.e., MMP-9, arginase-1 [Arg1], IL-10 (Mantovani et al., 2002)) and IL-1β, the intracellular accumulation of which was reported during the switch from M1-status toward M2-status of macrophages (Martinez et al., 2006; Pelegrin and Surprenant, 2009). Moreover, our data also showed higher phospho-STAT3 levels in J774A.1 macrophages grown in the conditioned media from the MMTV–Prune-1/Wnt1 cells, compared with those treated with the conditioned media from the MMTV–Wnt1 cells (Figure S9A). These data suggest that in a “Prune-1–overexpression status” in tumorigenic cells, modulation of the M1-M2 switch of macrophages occurs.

Then, to better dissect out how Prune-1 can take part in extracellular communication between TNBC cells and macrophages, we investigated whether Prune-1 can act in the modulation of M2-polarization through the release of soluble mediators. We focused on IL17-F due to its role in leukocyte infiltration and recruitment (i.e., macrophages, lymphocytes) (Yang et al., 2008; Giles et al., 2016; Starnes et al., 2001) (Shahrara et al., 2009; Nonaka et al., 2009). In this regard, the expression level of IL-17F was higher in MMTV–Prune-1/Wnt1, as compared with MMTV–Wnt1 cells (Figure 3D). Then, J774A.1 macrophages were grown in conditioned media derived from MMTV–Prune-1/Wnt1 cells or sh-IL17F-MMTV–Prune-1/Wnt1 (0.2-fold, Figures 3E and S9B). These data showed that J774A.1 macrophages treated with media from MMTV–Prune-1/Wnt1 cells expressed higher levels of Arg1, MMP9, IL-10, and IL-1β (Figure 3F), compared with untreated macrophages, thus confirming the RNAseq data. In contrast, lower levels of these M2-genes (i.e., Arg1, MMP9, IL-10, IL-1β) were found in the J774A.1 macrophages grown in the conditioned media from sh-IL17F-MMTV–Prune-1/Wnt1 cells (Figure 3F).

In contrast, J774A.1 macrophages were grown in the conditioned media derived from MMTV–Wnt1 cells or MMTV–Wnt1 cells overexpressing IL-17F (Figures 3G and S9C). These data showed that J774A.1 macrophages expressed higher levels of M2-associated genes (i.e., Arg1, MMP9, IL-10, IL-1β) only when they were grown in conditioned media from MMTV–Wnt1 cells overexpressing IL17F, as compared with those grown in conditioned media from MMTV–Wnt1 cells or untreated macrophages (Figure 3H). These data indicated a potential role for this inflammatory cytokine (i.e., IL17F) in the polarization process of immune cells.

Altogether, these data suggested that M2-polarization of TAMs (i.e., IL-10^High^, Arg1^High^, MMP9^High^, IL-1β^High^) in metastatic TNBC is evident in a Prune-1-overexpression status. Indeed, as shown here, Prune-1 takes part in the extracellular communication between tumorigenic and immune cells through secretion of IL-17F. Details on how this occurs will be the focus of future studies.

### Prune-1 induces macrophage polarization via extracellular vesicles

Tumor-derived EVs (including exosomes) have begun to emerge as new factors in tumor progression and organotropic metastatic dissemination. These have been reported to act via several mechanisms, including modulation of the antitumor immune response in the TME (Thery et al., 2009).

**Figure 4:**
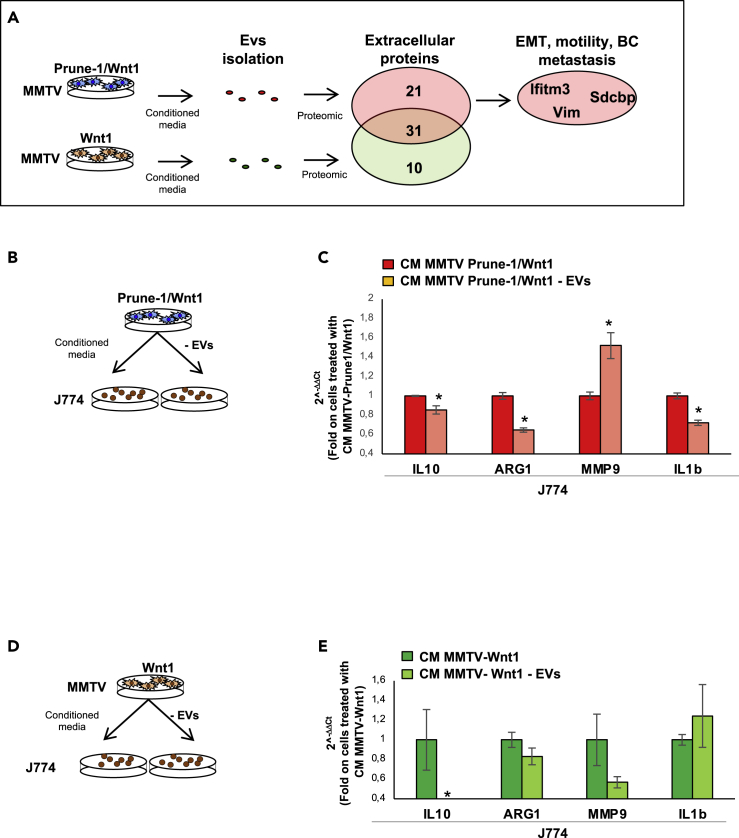
Prune-1 overexpressed in TNBC cells promotes M2-polarization of macrophages *in vitro* through modulation of EV-protein content (A) Representative scheme for proteomic analyses performed on extracellular vesicles (EVs). EVs were isolated from media from murine primary MMTV–Prune-1/Wnt1 and MMTV–Wnt1 cells. Proteomic analyses were performed on isolated EVs using label-free quantitative mass spectrometry. Data show 31 extracellular proteins in common between EVs from media of MMTV–Prune-1/Wnt1 and MMTV–Wnt cells, and 21 and 10 mutually exclusive extracellular proteins from MMTV–Prune-1-Wnt cells (red) and MMTV–Wnt1 cells (green). Data are representative of two independent experiments. (B and C) J774 macrophages were grown for 48 h in conditioned media collected (after 24 h) from MMTV–Prune-1/Wnt1 cells depleted or not in EVs from the culture supernatant (B). Real-time PCR analysis of some M2-associated genes, including IL-10, Arg-1, MMP-9, and IL-1β, in J774 macrophages grown for 48 h in conditioned media from MMTV–Prune-1/Wnt1 depleted (red) or not (light red) in EVs (C). ∗p < 0.05 in Student's t test compared with macrophages treated with conditioned media from MMTV-Prune-1/Wnt1 cells not depleted in EVs. (D and E) J774 macrophages were grown for 48 h in conditioned media collected (after 24 h) from MMTV–Wnt1 cells depleted or not in EVs from the culture supernatant (D). Real-time PCR analysis of some M2-associated genes, including IL-10, Arg-1, MMP-9, and IL-1β, in J774 macrophages grown for 48 h in conditioned media from MMTV–Wnt1 depleted (light green) or not (dark green) in EVs (E). ∗p < 0.05 in Student's t test compared with macrophages treated with conditioned media from MMTV-Wnt1 cells not depleted in EVs.

As Prune-1 induces M2-macrophage recruitment in the TME and promotes lung metastasis in the present GEMM of metastatic TNBC, we investigated whether EVs derived from MMTV–Prune-1/Wnt1 cells also contribute to these processes. To define a global picture of Prune-1–driven EV-proteins, a proteomic analysis was performed for those EVs secreted by both MMTV–Prune-1/Wnt1 and MMTV–Wnt1 cells. For this purpose, EVs were isolated (as previously described (Thery et al., 2006)) from media derived from both MMTV–Prune-1/Wnt1 and MMTV–Wnt1 cells. Subsequently, to define potential changes in EV-protein content in Prune-1-overexpressing cells, the extracellular proteins were analyzed using label-free quantitative mass spectrometry technology (as described in Supplemental Information (Cox et al., 2014)). These analyses showed that the EVs isolated from the media from both of these murine primary TNBC cells shared 31 proteins in common, whereas those derived from the MMTV–Prune-1/Wnt1 cells and the MMTV–Wnt1 cells had 21 and 10 mutually exclusive proteins, respectively (as summarized in Figures 4A and S9D)*.* Among the extracellular proteins secreted by the MMTV–Prune-1/Wnt1 cells, we identified some extracellular proteins linked to EMT, motility, and metastasis in BC, including vimentin (Vim; (Yamashita et al., 2013)), interferon-induced transmembrane protein 3 (Ifitm3; (Yang et al., 2013a)), and syndecan-binding protein (syntenin-1/Sdcbp (Yang et al., 2013b; Qian et al., 2013; Koo et al., 2002)) (Table 1). These data suggested that Prune-1 promotes distant metastasis in TNBC also by modulation of EV-protein content.

**Table 1:** Mutually exclusive proteins found into extracellular vesicles (EVs) derived from MMTV-Prune-1/Wnt1 cells

Gene symbol	Gene name	Accession	Number of distinct peptides	% of total peptides	Protein function (GO)
Htra1	Serine protease HTRA1	Q9R118	3	0.42%	Serine protease HTRA1; serine protease with a variety of targets, including extracellular matrix proteins such as fibronectin. HTRA1-generated fibronectin fragments further induce synovial cells to upregulate MMP1 and MMP3 production. May also degrade proteoglycans, such as aggrecan, decorin, and fibromodulin. Through cleavage of proteoglycans, may release soluble FGF-glycosaminoglycan complexes that promote the range and intensity of FGF signals in the extracellular space. Regulates the availability of insulin-like growth factors (IGFs) by cleaving IGF-binding proteins.
Cd81	CD81 antigen	P35762	2	0.31%	CD81 antigen; may play an important role in the regulation of lymphoma cell growth. May be involved in the acrosome reaction.
Anxa11	Annexin A11	P97384	8	0.86%	Required for midbody formation and completion of the terminal phase of cytokinesis (by similarity). Binds specifically to calcyclin in a calcium-dependent manner.
Emilin2	Elastin microfibril interfacer 2	Q8K482	5	0.87%	May be responsible for anchoring smooth muscle cells to elastic fibers and may be involved not only in the formation of the elastic fiber but also in the processes that regulate vessel assembly.
Ifitm3	Interferon-induced transmembrane protein 3	Q9CQW9	3	0.47%	IFN-induced antiviral protein that inhibits the entry of viruses to the host cell cytoplasm, permitting endocytosis, but preventing subsequent viral fusion and release of viral contents into the cytosol. Active against multiple viruses, including influenza A virus, SARS coronavirus (SARS-CoV), Marburg virus (MARV) and Ebola virus (EBOV), Dengue virus (DNV), West Nile virus (WNV) and human immunodeficiency virus type 1.
Vim	Vimentin	P20152	6	0.73%	Vimentins are class-III intermediate filaments found in various non-epithelial cells, especially mesenchymal cells. Vimentin is attached to the nucleus, endoplasmic reticulum (ER), and mitochondria, either laterally or terminally.
Stom	Stomatin	P54116	6	0.80%	Erythrocyte band 7 integral membrane protein; regulates ion channel activity and transmembrane ion transport. Regulates ASIC2 and ASIC3 channel activity.
Mme	Membrane metalloendopeptidase	Q61391	5	0.57%	Thermolysin-like specificity but is almost confined on acting on polypeptides of up to 30 amino acids. Biologically important in the destruction of opioid peptides such as Met- and Leu-enkephalins by cleavage of a Gly-Phe bond. Able to cleave angiotensin-1, angiotensin-2, and angiotensin 1–9.
Sdcbp	Syndecan-binding protein	O08992	5	0.71%	Multifunctional adapter protein involved in diverse array of functions including trafficking of transmembrane proteins, neuro and immunomodulation, exosome biogenesis, and tumorigenesis. Positively regulates TGFB1-mediated SMAD2/3 activation and TGFB1-induced epithelial-to-mesenchymal transition (EMT) and cell migration in various cell types. May increase TGFB1 signaling by enhancing cell-surface expression of TGFR1 by preventing the interaction between TGFR1 and CAV1 and subsequent CAV1-dependent internalization and degradation of TGFR1.
Vcp	Valosin-containing protein	Q01853	12	2.10%	Necessary for the fragmentation of Golgi stacks during mitosis and for their reassembly after mitosis. Involved in the formation of the transitional ER(tER). The transfer of membranes from the ER to the Golgi apparatus occurs via 50–70 nm transition vesicles that derive from part-rough, part-smooth transitional elements of the ER(tER). Vesicle budding from the tER is an ATP-dependent process.
Gnai2	Guanine nucleotide-binding protein alpha inhibiting 2	P08752	4	0.53%	Guanine nucleotide-binding proteins (G proteins) are involved as modulators or transducers in various transmembrane signaling systems. The G(i) proteins are involved in hormonal regulation of adenylate cyclase—they inhibit the cyclase in response to beta-adrenergic stimuli.
Chmp4b	Charged multivesicular body 4B	Q9D8B3	3	0.35%	Probable core component of the endosomal sorting required for transport complex III (ESCRT-III), which is involved in multivesicular bodies (MVBs) formation and sorting of endosomal cargo proteins into MVBs. MVBs contain intraluminal vesicles (ILVs) that are generated by invagination and scission from the limiting membrane of the endosome and mostly are delivered to lysosomes enabling degradation of membrane proteins, such as stimulated growth factor receptors, lysosomal enzymes, and lipids.
Igsf8	Immunoglobulin superfamily, member 8	Q8R366	6	0.87%	May play a key role in diverse functions ascribed to CD81 and CD9 such as oocytes fertilization or hepatitis C virus function. May regulate proliferation and differentiation of keratinocytes. May be a negative regulator of cell motility—suppresses T cell mobility coordinately with CD81, associates with CD82 to suppress prostate cancer cell migration, regulates epidermoid cell reaggregation and motility on laminin-5 with CD9 and CD81 as key linkers. May also play a role on integrin-dependent morphology and motility functions.
Anxa7	Annexin A7	Q07076	5	0.57%	Calcium-/phospholipid-binding protein that promotes membrane fusion and is involved in exocytosis
Ifitm2	Interferon-induced transmembrane 2	Q99J93	2	0.31%	IFN-induced antiviral protein that inhibits the entry of viruses to the host cell cytoplasm, permitting endocytosis, but preventing subsequent viral fusion and release of viral contents into the cytosol. Active against multiple viruses, including influenza A virus, SARS coronavirus (SARS-CoV), Marburg virus (MARV) and Ebola virus (EBOV), Dengue virus (DNV), and West Nile virus (WNV). Can inhibit influenza-virus-hemagglutinin protein-mediated viral entry, MARV and EBOV GP1,2-mediated viral entry, and SARS-CoV S-protein-mediated viral entry.
Ehd2	EH-domain containing 2	Q8BH64	3	0.40%	ATP- and membrane-binding protein that controls membrane reorganization/tubulation upon ATP hydrolysis. Plays a role in membrane trafficking between the plasma membrane and endosomes. Important for the internalization of GLUT4. Required for fusion of myoblasts to skeletal muscle myotubes. Required for normal translocation of FER1L5 to the plasma membrane.
Hist1h2bf	Histone cluster 1, H2bf	P10853	2	0.31%	Core component of nucleosome.
Ldha	lactate dehydrogenase A	P06151	6	0.80%	Ldha catalyzes the conversion of L-lactate and NAD to pyruvate and NADH in the final step of anaerobic glycolysis. The protein is found predominantly in muscle tissue and belongs to the lactate dehydrogenase family.
Rab11	Ras-related protein Rab-11B	P46638	5	0.53%	The small GTPases Rab are key regulators of intracellular membrane trafficking, from the formation of transport vesicles to their fusion with membranes. Rabs cycle between an inactive GDP-bound form and an active GTP-bound form that is able to recruit to membranes different set of downstream effectors directly responsible for vesicle formation, movement, tethering, and fusion. That Rab regulates endocytic recycling. Acts as a major regulator of membrane delivery during cytokinesis. Together with MYO5B and RAB8A participates in epithelial cell polarization.
Anxa4	Annexin A4	P97429	9	1.29%	Calcium-/phospholipid-binding protein that promotes membrane fusion and is involved in exocytosis.
Actbl2	Actin, beta-like 2	Q8BFZ3	6	0.62%	Actins are highly conserved proteins that are involved in various types of cell motility and are ubiquitously expressed in all eukaryotic cells.

The mutually exclusive extracellular proteins (analyzed using label-free quantitative mass spectrometry technology) secreted within EVs isolated from the media culture supernatant of MMTV–Prune-1/Wnt1 cells are listed.

Then, to investigate the potential role for these extracellular proteins secreted via EVs from MMTV–Prune-1/Wnt1 cells in the modulation of M2-associated genes (i.e., IL-10, MMP9, Arg1, IL-1β), J774A.1 macrophages were grown in conditioned media from MMTV–Prune-1/Wnt1 cells depleted of EVs (Figures 4B and S9E). These data showed reduction of IL-10, IL-1β, and Arg1 expression levels in the macrophages grown in the conditioned media without EVs, compared with those treated with complete supernatant (Figure 4C), whereas the expression levels of MMP9 were increased. This thus suggested different mechanisms of extracellular regulation of MMP9 in our model system. In contrast, the depletion of EVs from the media from MMTV–Wnt1 cells did not change the expression levels of M2-associated genes (i.e., MMP9, Arg1, IL-1β) in J774A.1 macrophages, with the exception of IL-10 (Figures 4D and 4E). The *in-vitro* data further indicated specific actions of those extracellular proteins secreted from Prune-1-overexpressing cells (i.e., MMTV–Prune-1/Wnt1 cells) in the modulation of M2-macrophage polarization.

To confirm these findings, we evaluated the polarization genes in J774A.1 macrophages grown in conditioned media derived from MMTV–Wnt1 cells that had been previously pre-treated with the supernatant of MMTV–Prune-1/Wnt1 cells containing or depleted in EVs (Figure S9F). These data showed increased levels of IL-10, Arg1, and IL-1β in J774A.1 macrophages when the MMTV–Wnt1 cells were grown in EV-containing media derived from MMTV–Prune-1/Wnt1 cells (Figure S9G).

In summary, Prune-1 is involved in extracellular mechanisms of communication between TNBC cells and immune cells (i.e., macrophages) not only through modulation of soluble mediators (e.g., IL-17F) but also through modulation of the EV-protein content.

### Genetics, RNA expression, and mutational rate in the TNBC microenvironment

**Figure 5:**
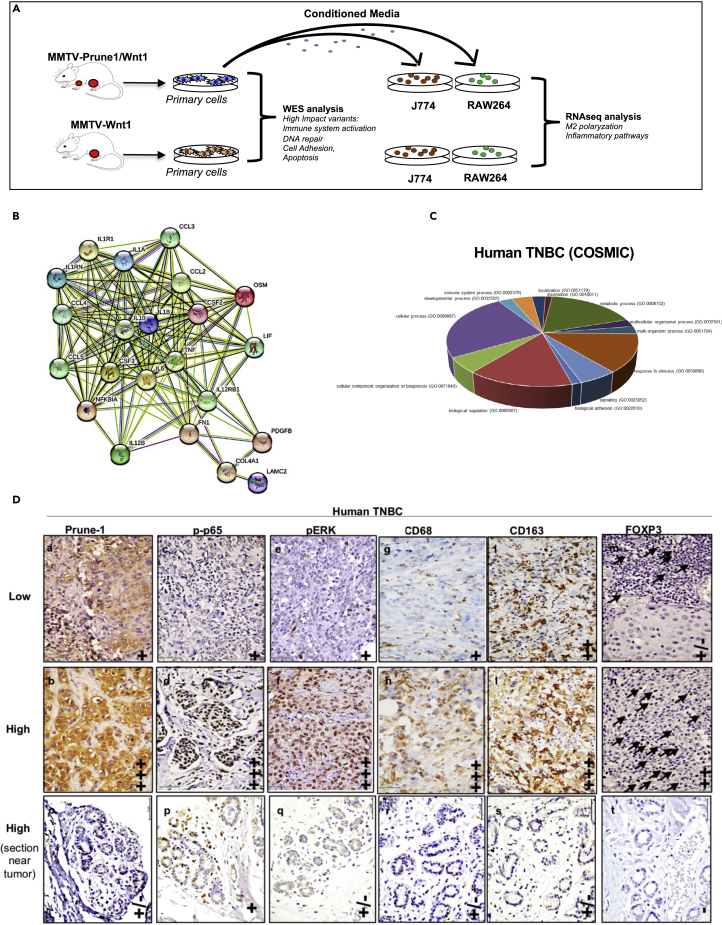
Mutational spectrum in TNBC cells overexpressing Prune-1 regulating M2-macrophages polarization (A) Representative scheme for the experimental design. DNA from MMTV–Prune-1/Wnt1 and MMTV–Wnt1 cells was used for next-generation sequencing analyses through a whole-exome sequencing approach. J774A.1 and Raw264.7 macrophages were grown in conditioned media collected from MMTV–Prune-1/Wnt1 and MMTV–Wnt1 cells for 48 h. Total RNA was extracted from these macrophages, and RNAseq analyses were performed. (B) The inflammatory protein network generated via Search Tool for Retrieval of Interacting Genes/Proteins (STRING) database using the “core genes” defined as the common genes that are overexpressed in both the J774A.1 and RAW264.7 macrophages grown in media obtained from MMTV–Prune-1/Wnt1 cells (as compared with MMTV–Wnt1 cells) shared by at least four of five enriched gene sets from each canonical pathway sub-collection (i.e., Biocarta, Kegg, Pid, Reactome, Naba). The protein interaction network was generated using the STRING database (confidence: 0.4; https://string-db.org/cgi/network.pl?taskId=41jaTsLzWNqT). (C) Pie chart illustrating the Gene Ontology (GO) term analysis of deleterious variants of the 39 genes in MMTV–Prune-1/Wnt1 cells (compared with MMTV–Wnt1 cells) and in the public database of human basal TNBC (COSMIC, v91). (D) Representative immunostaining (IHC) from “tumor” **(a–n)** and “near tumor” **(o–t)** sections of our TNBC tissue cohort (n = 138) derived from patients who underwent mastectomy, quadrantectomy, or metastectomy at the “Giovanni Pascale” National Cancer Institute of Naples (Italy) from 2003 to 2010, with antibodies against the following: Prune-1 **(a-b-o)**, phosphorylated-(Ser311)-p65 **(c-d-p)**, phosphorylated-ERK1/2 (i.e., phospho-[Thr202/Tyr204]-ERK1 and phospho-[Thr185/Tyr187]-ERK2) **(e-f-q)**, CD68 **(g-h-r)** and CD163 **(i-l-s)** (as markers for M2-TAMs), and FOXP3 (**m-n-t**) (as marker for Tregs). Magnification 40×. Scale bar: 50 μm; (−): negativity; (+): low immunopositivity; (++) medium immunopositivity; (+++): high immunopositivity.

To define a more global picture of the mechanism of communication between Prune-1-overexpressing TNBC cells and macrophages, we analyzed the mutational rates (through whole-exome sequencing [WES] analyses) in MMTV–Prune-1/Wnt1 cells and the global trascriptome (via RNAseq) of macrophages that received conditioned media from these tumorigenic cells (Figure 5A).

For this purpose, we took into account all of the common genes there were overexpressed in both J774A.1 and Raw264.7 macrophages that had been grown in the media obtained from MMTV–Prune-1/Wnt1 cells (as compared with MMTV–Wnt1 cells). Then, to identify a subset of “core genes” upregulated in macrophages in response to Prune-1 overexpression in TNBC cells, we compared the lists of genes from the leading edge of enriched gene-sets from each canonical pathway sub-collection (i.e., Biocarta, Kegg, Pid, Reactome, Naba) and selected those shared by at least four out of five of them (Table 2). A protein interaction network was generated using the Search Tool for the Retrieval of Interacting Genes/Proteins (STRING) database (confidence: 0.4; https://string-db.org/cgi/network.pl?taskId=41jaTsLzWNqT). This network identified proteins involved in immune system processes (GO:0002376), cytokine-mediated signaling pathways (GO:0019221), positive regulation of MAPK cascade (GO:0043410), positive regulation of cell migration (GO:0030335), regulation of inflammatory responses (GO:0050727), positive regulation of ERK1 and ERK2 cascade (GO:0070374), positive regulation of chemotaxis (GO:0050921), and positive regulation of tyrosine phosphorylation of STAT protein (GO:0042531) and macrophage chemotaxis (GO:0048246) (Figure 5B). Of interest, among these protein interaction networks derived from the “core genes” identified here, the expression levels of COL4A1, IL-10, IL1R1, and PDGFB were positively correlated with poor prognosis in BC patients in terms of 5-year survival data analyzed from the publicity available dataset of Breast Invasive Carcinoma (n = 1,075) from The Cancer Genome Atlas (TCGA) (Figure S10).

**Table 2:** The “core genes” upregulated in macrophages in response to Prune-1 overexpression in TNBC cells

Gene	Description	Annotation
CCL2	Chemokine (C-C motif) ligand 2	C-C motif chemokine 2; chemotactic factor that attracts monocytes and basophils but not neutrophils or eosinophils. Augments monocyte anti-tumor activity. Has been implicated in the pathogenesis of diseases characterized by monocytic infiltrates, such as psoriasis, rheumatoid arthritis, or atherosclerosis. May be involved in the recruitment of monocytes into the arterial wall during the disease process of atherosclerosis; belongs to the intracrine beta (chemokine CC) family
CCL3	Chemokine (C-C motif) ligand 3	C-C motif chemokine 3; monokine with inflammatory and chemokinetic properties. Binds to CCR1, CCR4, and CCR5. One of the major HIV-suppressive factors produced by CD8+ T cells. Recombinant MIP-1-alpha induces a dose-dependent inhibition of different strains of HIV-1, HIV-2, and simian immunodeficiency virus (SIV); belongs to the intercrine beta (chemokine CC) family
CCL4	Chemokine (C-C motif) ligand 4	C-C motif chemokine 4; monokine with inflammatory and chemokinetic properties. Binds to CCR5. One of the major HIV-suppressive factors produced by CD8+ T cells. Recombinant MIP-1-beta induces a dose-dependent inhibition of different strains of HIV-1, HIV-2, and simian immunodeficiency virus (SIV). The processed form MIP-1-beta(3-69) retains the abilities to induce down-modulation of surface expression of the chemokine receptor CCR5 and inhibit the CCR5-mediated entry of HIV-1 in T cells. MIP-1-beta(3-69) is also a ligand for CCR1 and CCR2 isoform B;
CCL5	Chemokine (C-C motif) ligand 5	C-C motif chemokine 5; chemoattractant for blood monocytes, memory T-helper cells, and eosinophils. Causes the release of histamine from basophils and activates eosinophils. May activate several chemokine receptors including CCR1, CCR3, CCR4, and CCR5. One of the major HIV-suppressive factors produced by CD8+ T-cells. Recombinant RANTES protein induces a dose-dependent inhibition of different strains of HIV-1, HIV-2, and simian immunodeficiency virus (SIV). The processed form RANTES(3-68) acts as a natural chemotaxis inhibitor and is a more potent inhibitor of HIV-1 infection.
COL4A1	Collagen, type IV, alpha 1	Collagen alpha 1(IV) chain; type IV collagen is the major structural component of glomerular basement membranes (GBM), forming a “chicken-wire” meshwork together with laminins, proteoglycans, and entactin/nidogen.
CSF2	Colony-stimulating factor 2 (granulocyte-macrophage)	Granulocyte-macrophage colony-stimulating factor; cytokine that stimulates the growth and differentiation of hematopoietic precursor cells from various lineages, including granulocytes, macrophages, eosinophils, and erythrocytes; belongs to the GM-CSF family.
CSF3	Colony-stimulating factor 3 (granulocyte)	Granulocyte colony-stimulating factor; granulocyte/macrophage colony-stimulating factors are cytokines that act in hematopoiesis by controlling the production, differentiation, and function of 2 related white cell populations of the blood: the granulocytes and the monocytes-macrophages. This CSF induces granulocytes; belongs to the IL-6 superfamily.
FN1	Fibronectin 1	Fibronectin type III domain containing; endogenous ligands
IL-10	Interleukin-10	Interleukin-10; inhibits the synthesis of a number of cytokines, including IFN-gamma, IL-2, IL-3, TNF, and GM-CSF produced by activated macrophages and by helper T cells; belongs to the IL-10 family
IL12B	Interleukin-12b	Interleukin-12 subunit beta; cytokine that can act as a growth factor for activated T and NK cells, enhance the lytic activity of NK/lymphokine-activated killer cells, and stimulate the production of IFN-gamma by resting PBMC; belongs to the type I cytokine receptor family. Type 3 subfamily.
IL12RB1	Interleukin-12 receptor, beta 1	Interleukin-12 receptor subunit beta-1; functions as an interleukin receptor that binds interleukin-12 with low affinity and is involved in IL-12 transduction. Associated with IL12RB2 it forms a functional, high-affinity receptor for IL-12. Associates also with IL23R to form the interleukin-23 receptor, which functions in IL-23 signal transduction probably through activation of the Jak-Stat signaling cascade; CD molecules
IL1A	Interleukin-1 alpha	Interleukin-1 alpha; produced by activated macrophages, IL-1 stimulates thymocyte proliferation by inducing IL-2 release, B-cell maturation and proliferation, and fibroblast growth factor activity. IL-1 proteins are involved in the inflammatory response, being identified as endogenous pyrogens, and are reported to stimulate the release of prostaglandin and collagenase from synovial cells.
IL1B	Interleukin-1 beta	Interleukin-1 beta; potent proinflammatory cytokine. Initially discovered as the major endogenous pyrogen, induces prostaglandin synthesis, neutrophil influx and activation, T cell activation and cytokine production, B-cell activation and antibody production, and fibroblast proliferation and collagen production. Promotes Th17 differentiation of T cells.
IL1R1	Interleukin-1 receptor, type I	Interleukin-1 receptor type 1; receptor for IL1A, IL1B, and IL1RN. After binding to interleukin-1 associates with the coreceptor IL1RAP to form the high-affinity interleukin-1 receptor complex that mediates interleukin-1-dependent activation of NF-κB, MAPK, and other pathways. Signaling involves the recruitment of adapter molecules such as TOLLIP, MYD88, and IRAK1 or IRAK2 via the respective TIR domains of the receptor/coreceptor subunits. Binds ligands with comparable affinity, and binding of antagonist IL1RN prevents association with IL1RAP to form a signaling complex.
IL1RN	Interleukin-1 receptor antagonist	Interleukin-1 receptor antagonist protein; inhibits the activity of interleukin-1 by binding to receptor IL1R1 and preventing its association with the coreceptor IL1RAP for signaling. Has no interleukin-1-like activity. Binds functional interleukin-1 receptor IL1R1 with greater affinity than decoy receptor IL1R2; however, the physiological relevance of the latter association is unsure; endogenous ligands.
IL6	Interleukin-6	Interleukin-6; cytokine with a wide variety of biological functions. It is a potent inducer of the acute phase response. Plays an essential role in the final differentiation of B-cells into Ig-secreting cells involved in lymphocyte and monocyte differentiation. Acts on B cells, T cells, hepatocytes, hematopoietic progenitor cells, and cells of the CNS. Required for the generation of T(H)17 cells. Also acts as a myokine. It is discharged into the bloodstream after muscle contraction and acts to increase the breakdown of fats and to improve insulin resistance. It induces myeloma and plas [ …]
LAMC2	Laminin, gamma-2	Laminin subunit gamma-2; binding to cells via a high-affinity receptor, laminin is thought to mediate the attachment, migration, and organization of cells into tissues during embryonic development by interacting with other extracellular matrix components. Ladsin exerts cell- scattering activity toward a wide variety of cells, including epithelial, endothelial, and fibroblastic cells.
LIF	Leukemia inhibitory factor	Leukemia inhibitory factor; LIF has the capacity to induce terminal differentiation in leukemic cells. Its activities include the induction of hematopoietic differentiation in normal and myeloid leukemia cells, the induction of neuronal cell differentiation, and the stimulation of acute-phase protein synthesis in hepatocytes; endogenous ligands.
NFKBIA	Nuclear factor of kappa light polypeptide gene enhancer in B cells inhibitor, alpha	NF-κB inhibitor alpha; inhibits the activity of dimeric NF-κB/REL complexes by trapping REL dimers in the cytoplasm through masking of their nuclear localization signals. On cellular stimulation by immune and proinflammatory responses, becomes phosphorylated promoting ubiquitination and degradation, enabling the dimeric RELA to translocate to the nucleus and activate transcription.
OSM	Oncostatin M	Oncostatin-M; growth regulator. Inhibits the proliferation of a number of tumor cell lines. Stimulates proliferation of AIDS-KS cells. It regulates cytokine production, including IL-6, G-CSF, and GM-CSF from endothelial cells. Uses both type I OSM receptor (heterodimers composed of LIPR and IL6ST) and type II OSM receptor (heterodimers composed of OSMR and IL6ST). Involved in the maturation of fetal hepatocytes, thereby promoting liver development and regeneration.
PDGFB	Platelet-derived growth factor, B polypeptide	Platelet-derived growth factor subunit B; growth factor that plays an essential role in the regulation of embryonic development, cell proliferation, cell migration, survival, and chemotaxis. Potent mitogen for cells of mesenchymal origin. Required for normal proliferation and recruitment of pericytes and vascular smooth muscle cells in the central nervous system, skin, lung, heart, and placenta. Required for normal blood vessel development and for normal development of kidney glomeruli.
TNF	Tumor necrosis factor	Tumor necrosis factor; cytokine that binds to TNFRSF1A/TNFR1 and TNFRSF1B/TNFBR. It is mainly secreted by macrophages and can induce cell death of certain tumor cell lines. It is potent pyrogen causing fever by direct action or by stimulation of interleukin-1 secretion and is implicated in the induction of cachexia. Under certain conditions it can stimulate cell proliferation and induce cell differentiation. Impairs regulatory T cells (Treg) function in individuals with rheumatoid arthritis via FOXP3 dephosphorylation. Upregulates the expression of protein phosphatase 1 (PP1).

The common genes there are found overexpressed in both J774A.1 and RAW264.7 macrophages grown in the media supernatant from MMTV-Prune-1/Wnt1 cells (as compared with those from MMTV-Wnt-1 cells) shared by at least 4 out of 5 enriched gene-sets coming from canonical pathway sub-collection (i.e., Biocarta, Kegg, Pid, Reactome and Naba) are listed. Gene, common gene name (HGNC); Description, gene description; Annotation, annotated using Sequence Ontology terms.

Then, we profiled the mutation spectra in our primary murine TNBC cells that overexpressed Prune-1. WES analyses were applied to both MMTV–Prune-1/Wnt1 cells and MMTV–Wnt1 cells. We found mutually exclusive coding variants with predicted higher/moderate impact on MMTV–Prune-1-Wnt1 cells (177 variants in 126 genes; see Methods; Table 3; Figure S11A). Among these, 39 gene variants were also found in the public data in the Catalog Of Somatic Mutations In Cancer (COSMIC, v91; released April 7, 2020) of human basal TNBC (426 samples collected) (see Table 3). Gene Ontology analyses performed on these human genes showed some deleterious variants involved in the activation of innate immune responses (leukocyte and macrophage activation; ANKHD1, FER1L5), cell adhesion (NEXN), apoptotic pathways (BID), and DNA repair (ERCC5) (Figure 5C). Then, among these 39 genes that were mutated in the human basal TNBC dataset available in COSMIC, we focused on those with unfavorable prognosis in BC patients in terms of their expression levels and 5-year survival. For this purpose, we took into account the analyses of survival data obtained from the publicly available dataset of breast invasive carcinoma (n = 1,075) from TCGA. Of interest, low expression levels of the PDE9A, ERCC5, Iqca, Sirpb1b, CD244, SP140, and PIP5k1b genes were associated with decreased 5-year survival rate in BC patients, thus suggesting their potential association with unfavorable prognosis (Figure S11B). The Rrs1 and A2M genes were excluded from this analysis because only one patient out of 426 had mutations in this gene in the public data of human basal TNBC in COSMIC (v91).

**Table 3:** Mutually exclusive coding variants with predicted higher/moderate impact in MMTV-Prune-1/Wnt1 cells

Chrom	#Pos	ALT	REF	Zygosity	Annotation	Gene Name	Feature_ID	HGVS.c	HGVS.p	dbSNP142_ID	Number of variants in human TNBC samples (COSMIC)
11	69588736	G	T	HET	stop_gained&splice_region_variant	Trp53	NM_011640.3	c.661G > T	p.Glu221∗	.	>200
11	69588393	C	G	HET	missense_variant	Trp53	NM_011640.3	c.396C > G	p.Cys132Trp	.	>200
17	31443854	GAGCTGAAAGCCGA	G	HET	frameshift_variant	Pde9a	NM_001163748.1	c.337_349delCTGAAAGCCGAAG	p.Leu113fs	.	38
1	82729275	T	C	HET	missense_variant	Mff	NM_029409.2	c.20T > C	p.Ile7Thr	.	18
1	82751668	C	G	HET	missense_variant	Mff	NM_029409.2	c.828C > G	p.Ile276Met	rs580786331	18
1	44167090	T	C	HET	missense_variant	Ercc5	NM_011729.2	c.1162T > C	p.Cys388Arg	rs8253429	16
2	71820255	G	GGA	HET	splice_donor_variant&intron_variant	Itga6	NM_001277970.1	c.643 + 1_643+2insGA	.	.	10
2	71833793	TGGTGAGTCCCTTTTCTGGGCGCTGGCTCAGTTCTCAGCTTGGCGATGAGCTCCTTGACATGATGAGCCACTGAGAAGGGGAAGGGTGCACTGGACAAAGGGACAAGTCGAGACACTGTCCAGCAGCCAGTGGGGACACTGGAGGGCTGGGGACAAGGTGGGGTCTTCCACTGCTACAGTGCATCTTTTAAGAAATATGTGTTTGATTTTATGCA	T	HET	frameshift_variant&splice_acceptor_variant&splice_donor_variant&splice_region_variant&splice_region_variant&splice_region_variant&intron_variant	Itga6	NM_001277970.1	c.1487_1488-2del	p.Cys496fs	.	10
6	118525854	G	A	HET	missense_variant	Ankrd26	NM_001081112.1	c.2354C > T	p.Ser785Leu	rs30264502	10
6	118525855	A	T	HET	missense_variant	Ankrd26	NM_001081112.1	c.2353T > A	p.Ser785Thr	rs30363420	10
6	118535096	C	G	HET	missense_variant	Ankrd26	NM_001081112.1	c.1564G > C	p.Glu522Gln	rs261478249	10
6	120895769	C	T	HET	missense_variant	Bid	NM_007544.3	c.426G > A	p.Met142Ile	.	10
1	90047844	G	T	HET	stop_gained	Iqca	NM_029122.2	c.2079C > A	p.Cys693∗	.	9
3	15548792	G	A	HET	stop_gained	Sirpb1b	NM_001173460.1	c.229C > T	p.Gln77∗	.	9
3	15548740	G	T	HET	missense_variant	Sirpb1b	NM_001173460.1	c.281C > A	p.Thr94Lys	.	9
3	15548774	G	A	HET	missense_variant	Sirpb1b	NM_001173460.1	c.247C > T	p.His83Tyr	.	9
3	15548789	A	G	HET	missense_variant	Sirpb1b	NM_001173460.1	c.232T > C	p.Ser78Pro	.	9
3	15548790	C	G	HET	missense_variant	Sirpb1b	NM_001173460.1	c.231G > C	p.Gln77His	.	9
3	15548854	G	T	HET	missense_variant	Sirpb1b	NM_001173460.1	c.167C > A	p.Ser56Tyr	.	9
12	98213021	T	TATACACTGAGGGCATCTCTCACACACCAGTGGTGATCGCCTATAACACTGATTGTGCTGGAAGCGTATACA	HET	frameshift_variant&stop_gained	Galc	NM_008079.4	c.1627_1628insTGTATACGCTTCCAGCACAATCAGTGTTATAGGCGATCACCACTGGTGTGTGAGAGATGCCCTCAGTGTAT	p.Asp543fs	.	9
1	36412593	C	T	HET	missense_variant	Fer1l5	NM_001277076.1	c.3781C > T	p.Arg1261Cys	rs32520256	8
2	73819145	G	GCAGTATGATAGCCAGACTTCTTCTGCATGGCAGTTACAGGGCAATCTTTATGAGCCAGAAGAAGCTGTTT	HET	frameshift_variant&stop_gained&splice_region_variant	Atf2	NM_001025093.2	c.1240_1241insAAACAGCTTCTTCTGGCTCATAAAGATTGCCCTGTAACTGCCATGCAGAAGAAGTCTGGCTATCATACTG	p.Ala414fs	.	8
2	73829942	C	CATTTTTGCTTCTGACTGGACTGGTTGAGGAGAGGAAGGGCCTGGGATTCCTGGAACACTAGGCACCATGG	HET	splice_acceptor_variant&intron_variant	Atf2	NM_001025093.2	c.775-2_775-1insCCATGGTGCCTAGTGTTCCAGGAATCCCAGGCCCTTCCTCTCCTCAACCAGTCCAGTCAGAAGCAAAAAT	.	.	8
5	3344393	C	CT	HET	frameshift_variant&stop_gained	Cdk6	NM_009873.2	c.27_28insT	p.Asp10fs	.	8
17	49488248	T	C	HET	missense_variant	Daam2	NM_001008231.2	c.916A > G	p.Met306Val	.	7
1	171573958	C	CAGAA	HET	frameshift_variant	Cd244	NM_018729.2	c.340_341insGAAA	p.Thr114fs	.	6
1	171573961	AGGCG	A	HET	frameshift_variant	Cd244	NM_018729.2	c.344_347delGCGG	p.Gly115fs	.	6
18	36648429	C	A	HET	missense_variant	Ankhd1	NM_175375.3	c.6533C > A	p.Ser2178Tyr	.	6
1	85635605	A	G	HET	missense_variant&splice_region_variant	Sp140	NM_001013817.2	c.1019A > G	p.Glu340Gly	.	5
11	3146254	TCG	T	HET	frameshift_variant	Sfi1	NM_030207.2	c.1378_1379delCG	p.Arg460fs	rs386949583	5
13	119450010	G	GAGAT	HET	splice_donor_variant&intron_variant	Paip1	NM_145457.4	c.842_842+1insAGAT	.	.	4
17	45568364	C	CCT	HET	frameshift_variant	Hsp90ab1	NM_008302.3	c.1928_1929dupAG	p.Ala644fs	.	4
17	45568460	G	A	HET	stop_gained	Hsp90ab1	NM_008302.3	c.1834C > T	p.Arg612∗	.	4
17	45568347	T	G	HET	missense_variant	Hsp90ab1	NM_008302.3	c.1947A > C	p.Lys649Asn	.	4
17	45568405	G	A	HET	missense_variant	Hsp90ab1	NM_008302.3	c.1889C > T	p.Pro630Leu	.	4
17	45568409	T	A	HET	missense_variant	Hsp90ab1	NM_008302.3	c.1885A > T	p.Asn629Tyr	.	4
17	45568434	C	T	HET	missense_variant	Hsp90ab1	NM_008302.3	c.1860G > A	p.Met620Ile	.	4
17	45568435	A	G	HET	missense_variant	Hsp90ab1	NM_008302.3	c.1859T > C	p.Met620Thr	.	4
19	24350205	C	A	HET	missense_variant	Pip5k1b	NM_008846.2	c.1286G > T	p.Ser429Ile	.	4
4	119422160	C	T	HET	missense_variant	Ppcs	NM_026494.3	c.194G > A	p.Arg65His	.	3
18	49755396	TC	T	HET	frameshift_variant	Dtwd2	NM_026854.3	c.142delG	p.Asp48fs	.	3
3	152247927	ACTTGCCATGTGCCTTCTCGCCTCCTCAAAGGAACGTCTCTCTTCTTCTAAGCGTCGTCTCGCTTCCTCTTCCGCTTGCTTCTTG	A	HET	frameshift_variant&splice_donor_variant&splice_region_variant&splice_region_variant&intron_variant	Nexn	NM_199465.2	c.579_657+5delCAAGAAGCAAGCGGAAGAGGAAGCGAGACGACGCTTAGAAGAAGAGAGACGTTCCTTTGAGGAGGCGAGAAGGCACATGGCAAG	p.Arg193fs	.	2
4	106585909	G	T	HET	missense_variant	Dhcr24	NM_053272.2	c.1140G > T	p.Gln380His	.	2
6	120958949	G	C	HET	missense_variant	Mical3	NM_001270475.1	c.4615C > G	p.Leu1539Val	.	2
6	121024818	T	A	HET	missense_variant	Mical3	NM_001270475.1	c.1712A > T	p.Asp571Val	.	2
11	98224575	C	CA	HET	frameshift_variant	Cdk12	NM_001109626.1	c.2546dupA	p.Asn850fs	.	2
13	70603400	C	CTATGGTTCCAGATTTCTTTCCTAGGGTTTCTATCTCTAGTGTTGCCTCGT	HET	frameshift_variant	Ice1	NM_144837.3	c.4565_4566insACGAGGCAACACTAGAGATAGAAACCCTAGGAAAGAAATCTGGAACCATA	p.Ile1523fs	.	2
17	78985311	G	T	HET	missense_variant	Prkd3	NM_001171004.1	c.331C > A	p.Leu111Ile	.	2
19	15907067	T	TACATAAAATCCTTGAGAATTATCAATGATCTCATAAATCATTTGGGATTTGATGGAG	HET	splice_acceptor_variant&intron_variant	Psat1	NM_177420.2	c.870-1_870insCTCCATCAAATCCCAAATGATTTATGAGATCATTGATAATTCTCAAGGATTTTATGT	.	.	2
1	9546377	C	T	HET	missense_variant	Rrs1	NM_021511.2	c.854C > T	p.Thr285Met	.	1
2	156267542	G	GCT	HET	splice_donor_variant&intron_variant	Phf20	NM_172674.2	c.808_808+1insCT	.	.	1
6	121671068	A	C	HET	missense_variant	A2m	NM_175628.3	c.3518A > C	p.Glu1173Ala	rs50350755	1
8	24962858	ACTGGCGGTCTGGAGACACCTGGGCCCC	A	HOM	exon_loss_variant&splice_acceptor_variant&splice_donor_variant&splice_region_variant&splice_region_variant&intron_variant&intron_variant	Adam9	NM_007404.2	c.2360-9_2376+1delGGGGCCCAGGTGTCTCCAGACCGCCAG	.	.	1
8	70065135	G	A	HET	missense_variant	Sugp1	NM_027481.2	c.1174G > A	p.Val392Met	.	1
12	76837652	G	GCCAAAGTAGAAGAGAAGATCCAGGAGGTCTTCAGTTCTTACAAGTTTAACCACCTTGTACCAAGGCTCATTT	HET	splice_donor_variant&intron_variant	Fntb	NM_145927.2	c.144 + 1_144+2insCCAAAGTAGAAGAGAAGATCCAGGAGGTCTTCAGTTCTTACAAGTTTAACCACCTTGTACCAAGGCTCATTT	.	.	1
15	77785100	G	T	HET	missense_variant	Myh9	NM_022410.3	c.1721C > A	p.Ala574Asp	.	1
19	60537503	G	GGCATTAGGCTGTAAATGTGCTTCTCTGCAACATGTTCCCAAAGGGTTATTATATTCCACAGTCAGGGATTTGCAACTGTTTGCTAGTGC	HET	splice_acceptor_variant&intron_variant	Cacul1	NM_030197.2	c.821-1_821insGCACTAGCAAACAGTTGCAAATCCCTGACTGTGGAATATAATAACCCTTTGGGAACATGTTGCAGAGAAGCACATTTACAGCCTAATGC	.	.	1
X	74303948	C	T	HET	missense_variant	Atp6ap1	NM_018794.4	c.1334C > T	p.Thr445Ile	.	1
X	74303956	C	T	HET	missense_variant	Atp6ap1	NM_018794.4	c.1342C > T	p.Arg448Cys	.	1
X	74303965	G	A	HET	missense_variant	Atp6ap1	NM_018794.4	c.1351G > A	p.Asp451Asn	.	1
X	74303977	C	T	HET	missense_variant	Atp6ap1	NM_018794.4	c.1363C > T	p.Pro455Ser	.	1
1	161150967	AT	A	HOM	frameshift_variant	Ankrd45	NM_028664.1	c.95delT	p.Leu32fs	.	
1	34536493	C	A	HET	missense_variant	Cfc1	NM_007685.2	c.247C > A	p.Pro83Thr	rs31623365	
1	46066957	C	T	HET	missense_variant	Dnah7b	NM_001160386.1	c.100C > T	p.Pro34Ser	rs32596568	
1	46066960	A	G	HET	missense_variant	Dnah7b	NM_001160386.1	c.103A > G	p.Ile35Val	rs31532497	
1	174027191	A	ACCTGGGCTGTAGAAGTCCCCGCCTGAGCTGTGGAAGTCCCCG	HET	splice_donor_variant&intron_variant	Ifi205	NM_172648.3	c.399 + 1_399+2insCGGGGACTTCCACAGCTCAGGCGGGGACTTCTACAGCCCAGG	.	.	
1	37897787	C	T	HET	missense_variant	Mrpl30	NM_027098.2	c.232C > T	p.Arg78Cys	rs30993281	
1	34560871	C	T	HET	missense_variant	Prss40	NM_009356.2	c.34G > A	p.Gly12Ser	rs48246656	
1	34560895	T	C	HET	missense_variant	Prss40	NM_009356.2	c.10A > G	p.Ile4Val	rs46691245	
2	90917709	G	GA	HOM	frameshift_variant	Ptpmt1	NM_025576.2	c.277dupT	p.Ser93fs	rs231270167	
2	156142243	C	CAGAGGAGTGGTGGCCTGTACGTCCATGTAGAGAGGTCGCAGCACTGCCTCTGCCTCTGAG	HET	splice_acceptor_variant&intron_variant	Nfs1	NM_010911.2	c.214-2_214-1insCTCAGAGGCAGAGGCAGTGCTGCGACCTCTCTACATGGACGTACAGGCCACCACTCCTCT	.	.	
2	86085529	C	A	HET	stop_gained	Olfr1037	NM_001011532.2	c.247G > T	p.Glu83∗	.	
2	86785928	G	T	HET	missense_variant	Olfr1093	NM_146366.1	c.197G > T	p.Arg66Leu	.	
2	90149325	G	T	HET	missense_variant	Olfr1270	NM_146985.2	c.680C > A	p.Ser227Tyr	.	
2	87108039	G	T	HET	missense_variant	Olfr259	NM_146770.2	c.347C > A	p.Ser116Tyr	.	
3	138445390	A	AGATCCTTGCCACTGCTGTTTGCCACACCGATGCCTATACCCTGAGCGGAGCTGACCCCGAGG	HET	splice_donor_variant&intron_variant	Adh5	NM_007410.3	c.114_114+1insATCCTTGCCACTGCTGTTTGCCACACCGATGCCTATACCCTGAGCGGAGCTGACCCCGAGGG	.	.	
3	20242108	C	A	HET	missense_variant	Cpa3	NM_007753.2	c.64G > T	p.Asp22Tyr	.	
3	95255225	ATACC	A	HET	frameshift_variant	Prune	NM_173347.2	c.1132_1135delGGTA	p.Gly378fs	.	
3	95255231	A	AT	HET	frameshift_variant	Prune	NM_173347.2	c.1129_1130insA	p.Val377fs	.	
3	95255263	C	CAT	HET	frameshift_variant	Prune	NM_173347.2	c.1097_1098insAT	p.Lys367fs	.	
3	95255264	GCT	G	HET	frameshift_variant	Prune	NM_173347.2	c.1095_1096delAG	p.Ser365fs	.	
4	138171107	C	A	HET	missense_variant	Eif4g3	NM_172703.3	c.2693C > A	p.Ala898Asp	.	
4	118726336	C	CT	HET	frameshift_variant	Olfr1340	NM_146304.2	c.96dupT	p.Leu33fs	rs243765225	
4	44994005	G	GTCTTTCTAATTGTTACCAAACATCTATTCTTGTCTTTCTACTTAGGACTATTGAATTTTAGCTGGGT	HET	frameshift_variant&stop_gained	Zbtb5	NM_001163283.1	c.1377_1378insACCCAGCTAAAATTCAATAGTCCTAAGTAGAAAGACAAGAATAGATGTTTGGTAACAATTAGAAAGA	p.His460fs	.	
4	120947371	GATCCT	G	HET	frameshift_variant	Zfp69	NM_001005788.3	c.297_301delAGGAT	p.Ala99fs	.	
5	52538271	G	A	HET	missense_variant	Lgi2	NM_144945.2	c.1345C > T	p.Arg449Trp	.	
5	36508431	C	CTTGCTGGAAAATGCAGAGATTAGGAAATGTTCTAGAAATGTCCAGTTTAATAAGCTCCAGACTGGCCTCT	HET	splice_acceptor_variant&intron_variant	Tbc1d14	NM_001113362.1	c.1510-1_1510insAGAGGCCAGTCTGGAGCTTATTAAACTGGACATTTCTAGAACATTTCCTAATCTCTGCATTTTCCAGCAA	.	.	
5	36507706	C	CATAACCCACATCCGGCCGGTAACAAGTATAAGCGCCCAAAATACTGTGCAACATGTCATGATACGGACCACCTTG	HET	splice_acceptor_variant&intron_variant	Tbc1d14	NM_001113362.1	c.1582-1_1582insCAAGGTGGTCCGTATCATGACATGTTGCACAGTATTTTGGGCGCTTATACTTGTTACCGGCCGGATGTGGGTTAT	.	.	
5	31200672	T	TTAGTACTACCATGTCCTGTGATTAAA	HET	frameshift_variant&stop_gained	Zfp513	NM_175311.4	c.361_362insTTTAATCACAGGACATGGTAGTACTA	p.Gln121fs	.	
6	130118998	C	CT	HOM	frameshift_variant	Klra21	NM_053151.1	c.617dupA	p.Glu207fs	.	
6	124230362	G	GAC	HOM	frameshift_variant	Vmn2r27	NM_001104642.1	c.318_319insGT	p.Leu107fs	.	
6	37536041	G	T	HET	stop_gained	Akr1d1	NM_145364.2	c.271G > T	p.Glu91∗	.	
6	116410808	C	T	HET	missense_variant	Alox5	NM_009662.2	c.1936G > A	p.Val646Ile	rs30121304	
6	124540928	G	T	HET	missense_variant	C1s1	NM_001097617.1	c.92C > A	p.Ser31Tyr	.	
6	115849644	C	T	HET	missense_variant	Mbd4	NM_010774.2	c.385G > A	p.Asp129Asn	rs30840549	
6	122881551	G	T	HET	missense_variant	Necap1	NM_026267.2	c.352G > T	p.Asp118Tyr	.	
6	121190114	G	T	HET	stop_gained	Pex26	NM_001304774.1	c.373G > T	p.Glu125∗	rs242534869	
6	115560065	C	CAGG	HET	disruptive_inframe_insertion	Tsen2	NM_199033.1	c.791_793dupAGG	p.Glu264dup	rs229576944	
6	121255361	G	T	HET	missense_variant	Usp18	NM_011909.2	c.343G > T	p.Val115Leu	rs30018341	
6	123309819	G	T	HET	missense_variant	Vmn2r19	NM_001104632.1	c.410G > T	p.Cys137Phe	.	
6	123315926	C	A	HET	missense_variant	Vmn2r19	NM_001104632.1	c.926C > A	p.Thr309Asn	rs243552529	
6	123336175	C	A	HET	missense_variant	Vmn2r19	NM_001104632.1	c.2203C > A	p.His735Asn	rs586785967	
6	123336359	G	C	HET	missense_variant	Vmn2r19	NM_001104632.1	c.2387G > C	p.Ser796Thr	rs234282974	
6	123386109	TTA	T	HET	frameshift_variant	Vmn2r20	NM_001104634.1	c.1713_1714delTA	p.Asn571fs	.	
6	123637517	A	ATAC	HET	disruptive_inframe_insertion	Vmn2r22	NM_001104637.1	c.1112_1113insGTA	p.His371delinsGlnTyr	.	
6	123704492	A	G	HET	missense_variant	Vmn2r23	NM_001104638.1	c.358A > G	p.Ser120Gly	.	
6	124062011	T	G	HET	missense_variant	Vmn2r26	NM_019917.2	c.2544T > G	p.His848Gln	.	
7	7002941	GC	G	HOM	frameshift_variant	Aurkc	NM_001080965.1	c.945delC	p.Cys315fs	.	
7	7002943	TA	T	HOM	frameshift_variant&stop_lost	Aurkc	NM_001080965.1	c.947delA	p.Ter316fs	.	
7	10050179	G	GT	HOM	frameshift_variant	Vmn2r50	NM_001105178.1	c.366dupA	p.Gln123fs	.	
7	47464285	G	GT	HET	frameshift_variant	Mrgpra2b	NM_153101.3	c.697dupA	p.Thr233fs	.	
7	47589308	C	T	HET	missense_variant	Mrgpra3	NM_153067.2	c.869G > A	p.Gly290Asp	rs264586948	
7	18521280	C	T	HET	missense_variant	Psg25	NM_054060.1	c.1310G > A	p.Cys437Tyr	.	
7	102020492	G	GGGATTGCTGAACATCCTCTTCAGGTCCACCTTCTCCTTGCAGTAGGGACATGTCTGCTTCTTTCCCACGATGCA	HET	splice_acceptor_variant&intron_variant	Rnf121	NM_029211.2	c.864-1_864insTGCATCGTGGGAAAGAAGCAGACATGTCCCTACTGCAAGGAGAAGGTGGACCTGAAGAGGATGTTCAGCAATCC	.	.	
7	79677445	G	GAATTTAAAACCGAGGAGGAGCTTCTGGCTTACATACATGACAACTACCAAAAGGCT	HET	splice_donor_variant&intron_variant	Ticrr	NM_029835.1	c.1900_1900+1insAATTTAAAACCGAGGAGGAGCTTCTGGCTTACATACATGACAACTACCAAAAGGCT	.	.	
7	11756824	G	GTCTT	HET	frameshift_variant	Vmn1r73	NM_134203.1	c.568_569insTCTT	p.Gly190fs	.	
7	11756825	GACTA	G	HET	frameshift_variant	Vmn1r73	NM_134203.1	c.570_573delACTA	p.Gly190fs	.	
7	11756747	T	TGGA	HET	disruptive_inframe_insertion	Vmn1r73	NM_134203.1	c.491_492insGGA	p.Ile164delinsMetAsp	.	
7	12152883	GTA	G	HET	frameshift_variant	Vmn1r78	NM_134208.2	c.421_422delTA	p.Tyr141fs	.	
7	12152888	T	TC	HET	frameshift_variant	Vmn1r78	NM_134208.2	c.425_426insC	p.Met142fs	.	
7	12615903	C	A	HET	missense_variant	Vmn2r54	NM_001081449.2	c.1751G > T	p.Arg584Leu	.	
7	41872539	GA	G	HET	frameshift_variant	Vmn2r58	NM_001105055.1	c.131delT	p.Phe44fs	.	
7	43189515	TCTTCG	T	HET	frameshift_variant	Zfp936	NM_001034893.1	c.403_407delCTTCG	p.Leu135fs	.	
8	27345850	T	A	HET	missense_variant	Tex24	NM_001013609.2	c.988T > A	p.Leu330Met	.	
8	26162481	C	CT	HET	frameshift_variant	Thap1	NM_199042.2	c.315_316insT	p.Ala106fs	rs236454042	
9	48945576	T	G	HET	missense_variant	Htr3b	NM_020274.4	c.601A > C	p.Ser201Arg	.	
9	120018055	AG	A	HET	frameshift_variant	Xirp1	NM_011724.3	c.1760delC	p.Pro587fs	.	
10	128269082	C	G	HET	missense_variant	Apof	NM_133997.2	c.104C > G	p.Ala35Gly	.	
10	43582730	G	GCT	HET	frameshift_variant	Cd24a	NM_009846.2	c.211_212dupCT	p.Leu72fs	.	
10	128408217	GCACCTGCAGGCACAGATGAGATATACTCAGACCCTCCTCAACATCGTGCTGCCAGGCCTGGTCTGCCATCTTCTCTAACGCCAGGCCCAGAGACT	G	HET	splice_acceptor_variant&inframe_deletion&splice_region_variant&splice_region_variant&splice_region_variant&intron_variant	Nabp2	NM_027257.1	c.372 + 4_375delAGTCTCTGGGCCTGGCGTTAGAGAAGATGGCAGACCAGGCCTGGCAGCACGATGTTGAGGAGGGTCTGAGTATATCTCATCTGTGCCTGCAGGTG	p.Val125_Gln126del	.	
10	128409348	G	GCCAGCCAGCAGGCCGGACTGTGCACACCGCCGAGCCACCCGCAGGGGGAGCCAGGGGGCTGGCTCAAATCCTCTCTTT	HET	splice_acceptor_variant&intron_variant	Nabp2	NM_027257.1	c.-23-1_-23insAAAGAGAGGATTTGAGCCAGCCCCCTGGCTCCCCCTGCGGGTGGCTCGGCGGTGTGCACAGTCCGGCCTGCTGGCTGG	.	.	
11	101157518	GAGGTGAGGATCTTCAACAGTGAAGCATGCTGGGGGGAAAGATCAGGCATGATTCAATCACGTGAAACATTCAACTCCGCATTCATTTGGGGACATCATATAGAGCGCATTA	G	HOM	frameshift_variant&splice_donor_variant&splice_region_variant&splice_region_variant&intron_variant	Tubg2	NM_134028.2	c.398_399 + 109del	p.Glu133fs	rs386970477	
11	5105856	C	A	HET	missense_variant	Rhbdd3	NM_001290492.1	c.1037C > A	p.Ala346Glu	.	
11	98042620	C	A	HET	splice_acceptor_variant&intron_variant	Stac2	NM_146028.4	c.496-1G > T	.	.	
13	26769616	TC	T	HET	frameshift_variant	Hdgfl1	NM_008232.3	c.472delG	p.Glu158fs	.	
14	79213479	A	AC	HOM	frameshift_variant	Zfp957	NM_001033215.3	c.878dupG	p.Gly294fs	rs249051530	
14	47311113	C	CTCTTACCATTAA	HET	splice_donor_variant&intron_variant	Mapk1ip1l	NM_178684.5	c.717_717+1insTCTTACCATTAA	.	.	
14	50424969	G	T	HET	missense_variant	Olfr739	NM_146668.2	c.449G > T	p.Cys150Phe	.	
15	80248399	G	T	HET	missense_variant	Mief1	NM_178719.5	c.481G > T	p.Ala161Ser	.	
17	34508444	C	A	HOM	stop_gained	Btnl6	NM_030747.1	c.1111G > T	p.Glu371∗	.	
17	36167489	C	CAT	HOM	frameshift_variant	Gm8909	NM_001081032.2	c.565_566insAT	p.Cys189fs	.	
17	36167491	CGG	C	HOM	frameshift_variant	Gm8909	NM_001081032.2	c.562_563delCC	p.Pro188fs	.	
17	36168196	CGGGCCGGGACACTGCGGTGGTGAAA	C	HOM	frameshift_variant&splice_acceptor_variant&splice_region_variant&splice_region_variant&intron_variant	Gm8909	NM_001081032.2	c.65-14_75delTTTCACCACCGCAGTGTCCCGGCCC	p.Val22fs	.	
17	48145527	AGACT	A	HET	frameshift_variant	9830107B12Rik	NM_001177896.1	c.236_239delAGTC	p.Gln79fs	.	
17	48128531	G	C	HET	missense_variant	9830107B12Rik	NM_001177896.1	c.505C > G	p.Leu169Val	.	
17	48145662	C	A	HET	missense_variant	9830107B12Rik	NM_001177896.1	c.105G > T	p.Lys35Asn	.	
17	34469182	TC	T	HET	frameshift_variant	Btnl4	NM_030746.1	c.1620delG	p.Gly540fs	.	
17	34469188	TCTTTGTCC	T	HET	frameshift_variant	Btnl4	NM_030746.1	c.1607_1614delGGACAAAG	p.Arg536fs	.	
17	34508222	A	AC	HET	frameshift_variant	Btnl6	NM_030747.1	c.1332_1333insG	p.Leu445fs	.	
17	34508224	G	T	HET	stop_gained	Btnl6	NM_030747.1	c.1331C > A	p.Ser444∗	.	
17	34508226	AG	A	HET	frameshift_variant	Btnl6	NM_030747.1	c.1328delC	p.Thr443fs	.	
17	34508231	T	A	HET	missense_variant	Btnl6	NM_030747.1	c.1324A > T	p.Arg442Trp	.	
17	35118059	G	A	HET	stop_gained	Csnk2b	NM_001303476.1	c.304C > T	p.Gln102∗	.	
17	23882480	A	AATG	HET	disruptive_inframe_insertion	Dcpp1	NM_019910.2	c.203_204insTGA	p.Lys68delinsAsnGlu	.	
17	36081275	C	T	HET	missense_variant	H2-Bl	NM_008199.2	c.877G > A	p.Glu293Lys	.	
17	35266687	GGC	G	HET	frameshift_variant&splice_region_variant	H2-D1	NM_010380.3	c.1046_1047delGC	p.Gly349fs	.	
17	35266693	C	CAGA	HET	disruptive_inframe_insertion	H2-D1	NM_010380.3	c.1052_1054dupAGA	p.Gln351_Ser352insLys	.	
17	33999478	G	A	HET	stop_gained	H2-K1	NM_001001892.2	c.403C > T	p.Gln135∗	.	
17	33997529	G	T	HET	missense_variant	H2-K1	NM_001001892.2	c.642C > A	p.Ser214Arg	.	
17	33997531	T	G	HET	missense_variant	H2-K1	NM_001001892.2	c.640A > C	p.Ser214Arg	.	
17	36119450	G	A	HET	missense_variant	H2-T10	NM_010395.7	c.598C > T	p.His200Tyr	.	
17	36119452	G	A	HET	missense_variant	H2-T10	NM_010395.7	c.596C > T	p.Ala199Val	.	
17	36030989	A	T	HET	stop_gained	H2-T23	NM_010398.3	c.716T > A	p.Leu239∗	.	
17	25311561	G	C	HET	missense_variant	Prss28	NM_053259.2	c.714G > C	p.Lys238Asn	.	
17	19811919	C	A	HET	missense_variant	Vmn2r103	NM_001104565.1	c.1954C > A	p.Gln652Lys	.	
17	20540922	T	G	HET	missense_variant	Vmn2r109	NM_001104571.1	c.2172A > C	p.Gln724His	.	
17	23308117	T	G	HET	missense_variant	Vmn2r114	NM_001102584.1	c.1440A > C	p.Lys480Asn	.	
17	33962099	C	CACACCAG	HET	frameshift_variant	Vps52	NM_172620.3	c.1314_1315insACACCAG	p.Asp439fs	.	
17	33961703	G	A	HET	missense_variant	Vps52	NM_172620.3	c.1216G > A	p.Val406Met	.	
17	33961725	A	G	HET	missense_variant	Vps52	NM_172620.3	c.1238A > G	p.Asp413Gly	.	
17	33962097	T	TCTG	HET	disruptive_inframe_insertion	Vps52	NM_172620.3	c.1312_1313insCTG	p.Tyr438delinsSerAsp	.	
17	33963235	C	T	HET	missense_variant	Vps52	NM_172620.3	c.1786C > T	p.Arg596Trp	.	
18	20086680	C	T	HET	stop_gained	Dsc1	NM_001291804.1	c.2432G > A	p.Trp811∗	.	
19	7215485	C	CAGGACCCCGAGCTGCTCCCGCGCCGGCT	HET	splice_acceptor_variant&intron_variant	Cox8a	NM_007750.2	c.115-2_115-1insAGCCGGCGCGGGAGCAGCTCGGGGTCCT	.	.	
19	11511735	C	CGT	HET	frameshift_variant	Gm8369	NM_001164202.1	c.433_434dupTG	p.Lys146fs	.	
19	6054981	GCCTGGGGAAAGATAAACTCCAGTCACCAGTTTCCAATCACAGGACCCCATGCCTTCATCCCTGGAAGATTCGGGTACACACCCAAGCCACTTA	G	HET	frameshift_variant&splice_acceptor_variant&splice_donor_variant&splice_region_variant&splice_region_variant&splice_region_variant&intron_variant	Mrpl49	NM_026246.3	c.354 + 2_355delTAAGTGGCTTGGGTGTGTACCCGAATCTTCCAGGGATGAAGGCATGGGGTCCTGTGATTGGAAACTGGTGACTGGAGTTTATCTTTCCCCAGG	p.Ala119fs	.	
X	74303936	T	C	HET	missense_variant	Atp6ap1	NM_018794.4	c.1322T > C	p.Leu441Pro	.	

The mutually exclusive coding variants with predicted higher/moderate impact (n.177 variants in n.126 genes) in MMTV-Prune-1/Wnt1 cells are listed. Among these, n.39 genes were also found mutated in the public data of human basal TNBC available on Catalog Of Somatic Mutations In Cancer (COSMIC, v91, released 07-APR-20). Chrom, chromosome; Pos, position (with the first base having position 1); ALT, alternate non-reference alleles; REF, reference base(s); Zygosity, Homo/Hetero; Annotation, annotated using Sequence Ontology terms; Multiple effects can be concatenated using “&”; Gene Name, common gene name (HGNC); Feature_ID, transcript ID (version number); HGVS.c, Variant using HGVS notation (DNA level); HGVS.p, HGVS notation (Protein level); dbSNP142_ID, dbSNP rsNo.

Of interest, all of the genes listed earlier (with the exception for ERCC5) were also mutated in the publicly available dataset of metastatic BC (n = 216 (Lefebvre et al., 2016)), with a total frequency of 3.7% (Cbioportal for cancer genomics; https://www.cbioportal.org) (Figure S11C), thus indicating a potential role in metastatic processes in BC.

### Dataset validation in a human cohort of TNBC patients positively correlated with M2-TAMs and distant metastasis

To further underpin these data, Prune-1 protein expression was analyzed in human TNBC by IHC on tissue micro-arrays using a collection cohort of primary TNBC specimens. In this cohort analysis, 138 TNBC samples were included (113 ductal, 25 not ductal). The patients age ranged from 24 to 93 years, with mean age 57 years. At surgery, tumors >2 cm were seen for 53% of these patients (72/136; for two patients this information was not available), and metastatic lymph nodes were seen for 40.2% of patients (55/137; for one patient this information was not available). The tumor gradings were as follows: grade 3, 87.7% (121/138) and grade 1 or 2, 12.3% (17/138). The expression of the proliferation marker Ki67 was high (>20%) in 80.6% of patients (108/134) and low (≤20%) in 19.4% of patients (26/134; for four patients this information was not available; see Table 4).

**Table 4:** Relation between Prune-1 expression and clinicopathological features of TNBC patients in the TNBC tissue cohort

	Prune expression^a^ (n = 138)		p value	R pearson
	Low	High		
Age				
<40	8 (44.4%)	10 (55.6%)	0.550	0.017
>40 ≤ 60	32 (56.2%)	25 (43.8%)		
>60	30 (47.6%)	33 (52.4%)		
Histotype				
Ductal	59 (52.2%)	54 (47.8%)	0.457	0.063
Not ductal	11 (44%)	14 (56%)		
Size				
≤2 cm	31 (48.4%)	33 (51.6%)	0.860	−0.012
>2 ≤ 5	32 (52.4%)	29 (47.6%)		
>5	5 (45.4%)	6 (54.6%)		
NA	2 (100%)	0 (0%)		
LNM				
Negative	45 (54.9%)	37 (45.1%)	0.197	0.110
Positive	24 (43.6%)	31 (54.4%)		
NA	1 (100%)	0 (0%)		
Lung metastasis				
Negative	38 (57.6%)	28 (42.4%)	**0.027**^b^	0.254
Positive	2 (20%)	8 (80%)		
NA	30 (48.4%)	32 (51.6%)		
Grading				
G1-2	14 (82.3%)	3 (17.7%)	**0.005**^b^	0.237
G3	56 (46.3%)	65 (53.7%)		
Ki67				
<20%	19 (73.1%)	7 (26.9%)	**0.011**^b^	0.219
≥20%	49 (54.6%)	59 (45.4%)		
NA	2 (50%)	2 (50%)		
Disease progression				
No	37 (59.7%)	25 (40.3%)	**0.031**^b^	0.212
Yes	16 (38.1%)	26 (61.9%)		
NA	17 (50%)	17 (50%)		

The clinicopathological parameters of the patients from our TNBC cohort grouped according to their Prune-1 protein expression levels are shown (n. 138 patients). Prune-1 overexpression was found positively correlated with Ki67 proliferative index (p = 0.011; R = 0.219), high grade (p = 0.005; R = 0.237), disease progression (p = 0.031; R = 0.212), and the presence of lung metastasis (p = 0.027; R = 0.254). Any statistically correlations were found between Prune-1 levels and patients age, lymph node metastases, and tumor histotype and size. In bold the statistically significant p-values.

LMN, lymph node metastases; G1, Grade 1 or low grade (sometimes also called well differentiated); G2, Grade 2 or intermediate/moderate grade (moderately differentiated); G3, Grade 3 or high grade (poorly differentiated); NA, not available.

a Prune-1 protein expression is based on the intensity and percentage of expression.

b Statistical significant association (i.e., p < 0.05).

Prune-1 protein expression was detected in 89.9% (124/138) of the samples from this tissue cohort. In 50.7% of the samples (68/138) there was low Prune-1 expression, and in 49.3% of the samples (70/138) there was high Prune-1 expression (Figure 5D, a, b). These findings indicated that about 50% (i.e., 49.3%) of the TNBC samples in this tissue cohort showed overexpression of Prune-1 protein.

Statistical analysis of Prune-1 protein expression (i.e., based on intensity, percent expression) in terms of the other clinicopathological parameters in this TNBC tissue cohort indicated that Prune-1 was positively correlated with Ki67 proliferative index (p = 0.011; R = 0.219), high-grade (p = 0.005; R = 0.237), disease progression (p = 0.031; R = 0.212) and presence of lung metastasis (p = 0.027; R = 0.254), as shown in Table 4. No significant correlations were found between Prune-1 levels and patients age, lymph node metastases, and tumor histotype and size (Table 4). Altogether, these data further supported the concept that overexpression of Prune-1 can be used to predict lung metastases in TNBC.

Furthermore, we investigated the potential relationships between Prune-1 and the tumorigenic signaling pathways that are responsible for the aggressive behavior of TNBC. For this purpose, we took into account the nuclear localization of the MAPK and NF-κB effectors, due to their previously reported expression and correlations with poor prognosis in TNBC (Kim et al., 2016; Bartholomeusz et al., 2012). Our studies showed positive significant relationships between Prune-1 and phosphorylated-p65 (phospho-[Ser311]-p65; p < 0.00001; R = 0.421) and phosphorylated-ERK1/2 (phospho-[Thr202/Tyr204]-ERK1, phospho-[Thr185/Tyr187]-ERK2; p = 0.003; R = 0.277), as shown in Table 5 and Figure 5D, c–f. Overall, these data highlighted the involvement of Prune-1 in TNBC progression through the MAPK signaling cascade (i.e., phospho-ERK1/2) and through the NF-κB inflammatory pathway (i.e., phospho-p65), as identified in this TNBC dataset by IHC analysis.

**Table 5:** Correlation analyses between Prune-1 and tumorigenic signaling pathways (phospho-p65 and phospho-ERK ½ in TNBC patient cohort)

	Prune expression^a^ (n = 113)		p value	R pearson
	Low	High		
Phospho-p65^b^				
Low	36 (78.3%)	10 (21.7%)	<0.00001∗∗	0.421
High	29 (44.3%)	56 (65.7%)		
NA	5 (71.4%)	2 (28.6%)		
Phospho-ERK ½^c^				
Low	40 (60.6%)	26 (39.4%)	0.003∗∗	0.277
High	17 (32.7%)	35 (67.3%)		
NA	13 (65%)	7 (35%)		

The nuclear localization of MAPK and NF-kB effectors (i.e., phosphorylated-ERK1/2 and phosphorylated-p65, respectively) were evaluated in TNBC patients (n. 113) in our tissue cohort grouped according to their Prune-1 protein expression levels. Prune-1 overexpression was found positively correlated with phosphorylated-p65 (phospho-[Ser311]-p65; p < 0.00001; R = 0.421) and phosphorylated-ERK1/2 (phospho-[Thr202/Tyr204]-ERK1, phospho-[Thr185/Tyr187]-ERK2; p = 0.003; R = 0.277).

MAPK, mitogen-activated protein kinase; NF-kB, nuclear factor kappa-light-chain-enhancer of activated B cells.

A statistically significant association: i.e., ∗∗p < 0.005.

a Prune-1 protein expression is based on the intensity of expression.

b Phosphorylated-(Ser311)-p65.

c Phosphorylated-(Thr202/Tyr204)-ERK1 and phosphorylated-(Thr185/Tyr187)-ERK2.

Following these statistical associations, we investigated additional correlations between Prune-1 expression and immune-cell infiltration. We used immunosuppressive pro-tumorigenic M2-TAMs (i.e., CD68^+^ CD163^+^ cells) due to their already identified prognostic role in TNBC in the tumors with higher risk of metastatic dissemination (Sousa et al., 2015; Yuan et al., 2014). These correlation analyses of our dataset showed that patients with TNBC (n = 32) overexpressing Prune-1 were also characterized by higher numbers of tumor-infiltrating TAMs (i.e., CD68^+^ cells; Figure 5D, g–h; p = 0.014, R = 0.433; in Table 6) and with a trend to significant association with CD163+ cells (p = 0.07, R = 0.32; see Figure 5D, i–l), a marker of pro-tumorigenic M2-polarized TAMs (Spano and Zollo, 2012), and with FOXP3, a marker of Tregs (p = 0.08; see Figure 5D, m–n). Of interest, we did not find any strong positivity of inflammatory pathways (i.e., p65-NF-κB), p-ERK, or the presence of immune infiltrating cells (i.e., CD68, CD163, FOXP3) in the sections near tumors with high expression of Prune-1 (Figure 5D, o–t). Altogether, these results indicate that Prune-1 is a potential biomarker related to TNBC subtypes with the poorest outcomes, as characterized by higher infiltrating M2-TAMs (CD163^+^) and Tregs (FOXP3^+^) (Adams et al., 2017). However, to define an immunologically “cold” or “hot” TME, this analysis is also supported by CD4, CD8, and PDL1 to evaluate the TILs environment and the levels of immunosuppressive markers (Adams et al., 2017). Here, we focused mainly on TAMs within the TME of TNBC due to their prognostic value (Sousa et al., 2015; Yuan et al., 2014).

**Table 6:** Correlation analyses between Prune-1 and M2-polarized tumor-associated macrophages (M2-TAMs) within the TME of TNBC patients

	Prune-1 (n = 32)^a^		p value	R pearson
	Low	High		
CD68				
Low	7 (87.5%)	1 (22.5%)	0.014^b^	0.433
High	9 (37.5%)	15 (62.5%)		
CD163				
Low	5 (83.3%)	1 (16.7%)	0.070^a^	0.320
High	11 (62.3%)	15 (57.7%)		

The expression of CD68+ and CD163+ cells (i.e., M2-TAMs) in the tumor from the patients belonging to our TNBC cohort (n.32) grouped according to their Prune-1 protein expression levels are shown. The overexpression of Prune-1 was found correlated with the presence of CD68+ cells, marker of TAMs (p = 0.014, R = 0.433). A positive statistical trend between the overexpression of Prune-1 and CD163+ cells (M2-TAMs marker) was also shown (p = 0.07, R = 0.32).

a Prune-1 protein expression is based on the intensity of expression. Statistical trend toward significance.

b A statistically significant association (i.e., p < 0.05).

In conclusion, the clinico-immunopathological correlation studies performed on our TNBC patient cohort suggest that overexpression of Prune-1 is significantly associated with the activation of proliferative and inflammatory pathways (i.e., MAPK, NF-κB), and recruitment of immunosuppressive cells with pro-tumorigenic functions (i.e., M2-TAMs) in the TME, in those patients with TNBC with high-grade tumorigenesis progression and lung metastases. At this time, we focused on these immune cell populations (i.e., TAMs) because of their negative prognostic significance, as previously reported in TNBC patients (Adams et al., 2017).

### Pharmacological inhibition of Prune-1 through AA7.1 reduces metastatic foci *in vivo* via inhibition of M2 polarization of macrophages

To further address the role of Prune-1 in M2 polarization of macrophages, we used an anti-Prune-1 molecule (AA7.1) that has been previously shown to enhance Prune-1 degradation and to impair TGF-β signaling in metastatic medulloblastoma (Ferrucci et al., 2018).

**Figure 6:**
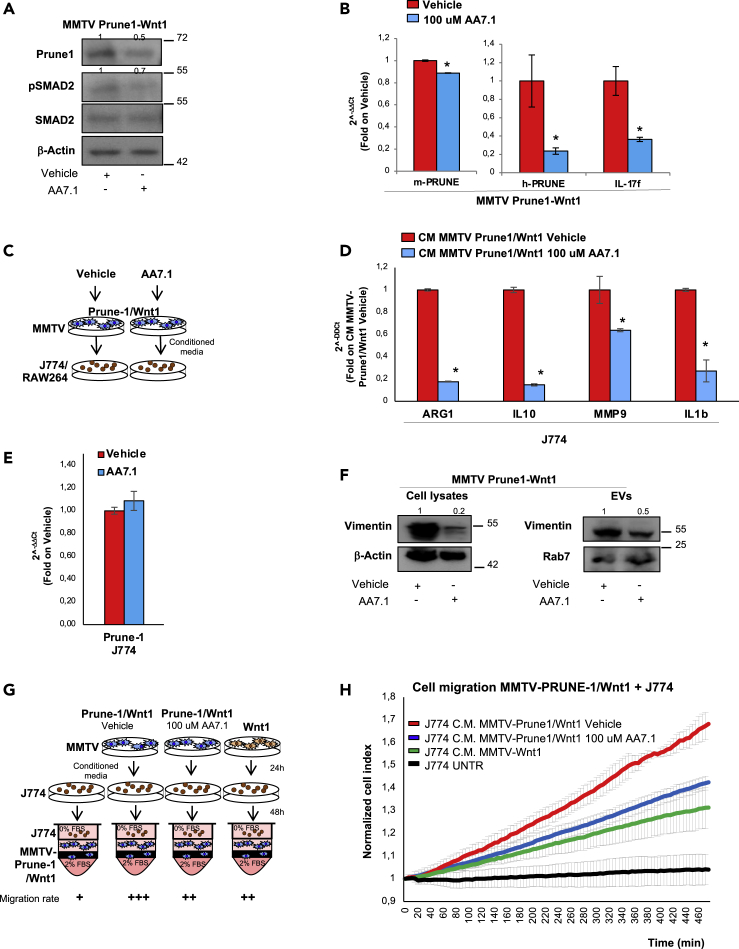
The AA7.1 anti-Prune-1 agent affects macrophages polarization *in vitro* (A) Immunoblotting on total protein lysates of MMTV–Prune-1/Wnt1 cells treated with AA7.1 (100 μM, for 12 h) or PBS as the vehicle control, with the antibodies as indicated. (B) Real-time RT-PCR of total RNA extracted from MMTV–Prune-1/Wnt1 cells treated with AA7.1 (100 μM, for 12 h) or PBS as the vehicle control, to detect human and murine Prune-1 and murine IL-17F. ∗, p < 0.05 in Student's t test compared with vehicle-treated MMTV–Prune-1/Wnt1 cells. (C and D) Schematic representation of experimental design. J774A.1 macrophages were grown for 48 h in conditioned media collected from MMTV–Prune-1/Wnt1 cells treated with AA7.1 (100 μM, for 12 h) or PBS as the vehicle control (C). Real-time PCR analysis of some M2-associated genes, including IL-10, Arg-1, MMP-9, and IL-1β, in J774A.1 macrophages grown for 48 h in conditioned media collected from MMTV–Prune-1/Wnt1 cells treated with AA7.1 (blue) or PBS, as the vehicle control (red) (D). ∗p < 0.05 in Student's t test compared with macrophages grown in conditioned media collected from vehicle-treated MMTV–Prune-1/Wnt1 cells. (E) Real-time RT-PCR of total RNA extracted from J774A.1 macrophages treated with AA7.1 (100 μM, for 48 h). (F) Immunoblotting of total protein lysates or EVs of MMTV–Prune-1/Wnt1 cells treated with AA7.1 (100 μM) or PBS as the vehicle control, with the antibodies as indicated. (G and H) Co-culture experiments to measure migration rates of MMTV–Prune-1/Wnt1 cells (using 2% FBS as chemoattractant) in the presence of J774A.1 macrophages previously grown in conditioned media collected from MMTV–Prune-1/Wnt1 cells (untreated or treated with AA7.1) and MMTV–Wnt1 cells. Untreated macrophages were used as negative control (G). Normalized Cell Index as a measure of cell migration of MMTV–Prune-1/Wnt1 cells, as generated by the xCELLigence RTCA software. Migration kinetics were monitored (every 5 min) in response to the presence of untreated macrophages (black) or J774A.1 macrophages previously grown in conditioned media collected from vehicle-treated or AA7.1-treated MMTV–Prune-1/Wnt1 cells (red and blue, respectively) and MMTV–Wnt1 cells (green). The grading on migration rate is evaluated as the difference of Cell Index values observed at the end of the experiment. +++, 1.6; ++, 1.35; +/−, 1.04.

Here, the potential immunomodulatory activity exerted by AA7.1 was tested in primary murine TNBC cells. AA7.1-treated MMTV–Prune-1/Wnt1 cells showed decreased levels of Prune-1 protein (Figure 6A, 50% reduction) and mRNA (Figure 6B) levels, with a major action on degradation of h-Prune-1. Reduced levels of phospho-SMAD2 (Figure 6A) and IL-17F (Figure 6B) were also shown in the same AA7.1-treated cells. These data indicate that AA7.1 can reduce the activation of TGF-β signaling and the secretion of this inflammatory cytokine that has previously been shown to be positively modulated by Prune-1.

This immunomodulatory action of AA7.1 was then studied by evaluating changes in the expression levels of M2-associated genes in macrophages (J774A.1) grown in conditioned media from AA7.1-treated MMTV–Prune-1/Wnt1 cells (Figure 6C). Of importance, there were decreased levels of IL-10, Arg1, IL-1β, and MMP-9 in these J774A.1 macrophages grown in culture media derived from the AA7.1-treated cells compared with the vehicle control (Figure 6D). To exclude any potential carry-over of AA7.1 in the tumor-cell-conditioned medium to the macrophages, we tested for AA7.1 downregulation of Prune-1 mRNA expression in J774A.1 macrophages. These macrophages treated with AA7.1 do not show downregulation of endogenous murine Prune-1 (Figure 6E), thus indicating that the changes in the cytokine levels in the recipient J774A.1 macrophages was not due to any direct effects of AA7.1 on these immune cells, thus excluding potential carry-over effects (at least at the AA7.1 concentration used in the assay).

Of interest, there was also modulation of the extracellular protein content released from AA7.1-treated MMTV–Prune-1/Wnt1 cells. Here, there were reduced Vimentin protein levels for both the AA7.1-treated MMTV–Prune-1/Wnt1 cells and in the extracellular vesicles secreted from these treated cells (Figure 6F).

Overall here, we have shown that AA7.1 can negatively modulate the cross-talk between TNBC and macrophages, thus affecting the activation of M2-associated genes via downregulation of Prune-1, inhibition of the TGF-β pathway, reduction of IL-17F in TNBC cells, and modulation of the extracellular protein content.

Next, to investigate whether M2-polarized macrophages can affect the migration rates of TNBC cells, we performed co-culture transwell migration assays using (1) J774A.1 macrophages previously polarized using conditioned media from MMTV–Prune-1/Wnt1 cells untreated or treated with AA7.1 and (2) MMTV-Wnt1 cells (Figure 6G). Untreated macrophages were used as the negative control. This system allowed the migration rates of MMTV–Prune-1/Wnt1 cells (using only 2% FBS as chemoattractant) to be monitored in the presence of M2-polarized macrophages (Figure 6H). These data showed increased migration rates only for MMTV–Prune-1/Wnt1 cells co-cultured with J774A.1 macrophages previously grown in conditioned media from untreated MMTV–Prune-1/Wnt1 cells (Figure 6H). To this end, we show that the M2-polarized macrophages can increase the motility of the TNBC cells, thus indicating that a bidirectional communication is in place between macrophages and tumorigenic cells.

Furthermore, we also confirmed *in vivo* that both silencing and pharmacological inhibition (via AA7.1) of Prune-1 inhibited TNBC growth and M2-TAM polarization. For this, we used syngeneic orthotopic models with murine metastatic 4T1 TNBC cells. In the first trials, immunocompetent BALB/c mice were implanted in the mammary gland with Prune-1-silenced or EV control 4T1 clones that stably expressed the firefly luciferase gene (4T1-LUC) (Figure S12A). These syngeneic orthotopic mice were imaged weekly for tumor growth, as evaluated using bioluminescence acquisition with an imaging system (IVIS 3D Illumina; Xenogen/Caliper). At 14 and 21 days from tumor implantation, there was significant decrease in tumor growth (Figure S12B). Of importance, in the tumor tissues derived from the Sh-Prune-1-4T1 implanted mice there was a reduction of the number of infiltrating M2-TAMs (i.e., CD68+. CD163+ cells) in the TME (Figures S12C and S12D).

Similar results were obtained *in vivo* through pharmacological inhibition of Prune-1 using AA7.1. Female immunocompetent BALB/c mice were injected with 4T1-LUC cells. At 14 days from tumor implantation (i.e., once the tumors were established), the mice were grouped according to their bioluminescence values and injected intraperitoneally with 60 mg/kg AA7.1 daily or with phosphate-buffered saline (PBS) as the vehicle control (Figure S12E). Tumor growth was monitored weekly by bioluminescence acquisition. These *in-vivo* data showed significant reduction of tumor growth in the AA7.1-treated mice at 35 and 42 days from tumor implantation (Figure S12F). At the end of the experiments (i.e., 42 days from tumor implantation), the primary tumors were dissected out and embedded in paraffin for IHC analysis. These data showed significant reduction of CD163+ cells, but not CD68+ cells (Figures S12G and S12H), thus suggesting inhibition of the M2-polarization switch of macrophages in the TME.

**Figure 7:**
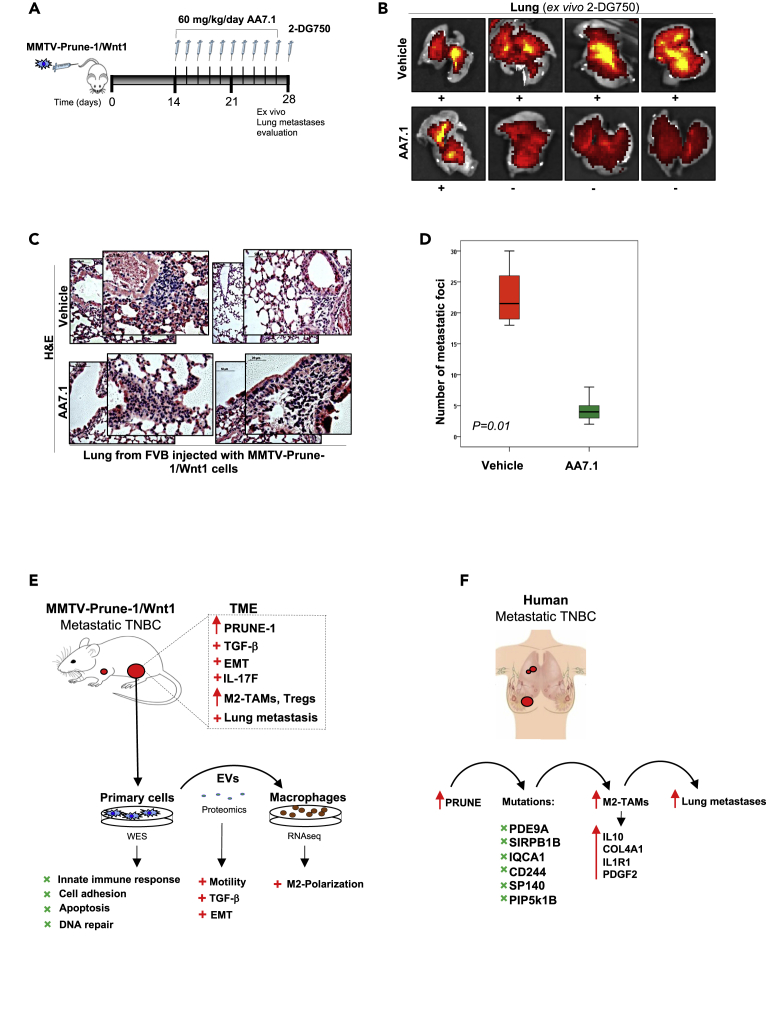
Hypothesized mechanisms of action of Prune-1 in the tumor microenvironment in the cross-talk between TNBC cells and macrophages (A–D) Schematic representation of the *in-vivo* trial. MMTV–Prune-1/Wnt1 cells (1 ×10^5^) were injected via the tail vein in eight immunocompetent syngeneic mice (strain FVB). After 14 days from cell injection, the mice were grouped according to their weight and AA7.1 (60 mg/kg/day, IP) or PBS (as vehicle control) was administered daily. At 14 days from treatment start (i.e., 28 days from cell injection), the mice were injected with a fluorescent imaging probe (XenoLight RediJect 2-DG-750; Perkin Elmer) for *ex-vivo* targeting of the tumorigenic cells (A). Positive fluorescence signals were detected in the lungs derived from all of the control mice (treated with PBS as vehicle) and in one of four AA7.1-treated mice (i.e., 25%) (B). Hematoxylin/eosin staining performed on sections of lung tissue from AA7.1-treated mice compared with controls. Magnification: 4x, 20x, 40x; Scale bars: 200 μm, 50 μm, 20 μm (C). Box plot (generated with the SPSS software) showing differences in the number of metastatic foci detected in the lungs from AA7.1-treated mice (green), compared with the control group (red) (D). (E and F) The proposed mechanism of action of Prune-1 at the interplay between tumorigenic cells and TAMs in TME is shown for the murine model of metastatic TNBC (i.e., MMTV–Prune-1/Wnt1). Prune-1 acts in metastatic TNBC by promoting activation of intracellular pathways (i.e., TGF-β) and in a paracrine manner through the release of extracellular inflammatory molecules (i.e., IL-17F) and modulation of extracellular vesicle protein content (i.e., Sdcbp, Vim, Iftm3) involved in EMT and metastasis. Furthermore, mutational analyses in murine primary TNBC cells overexpressing Prune-1 (i.e., MMTV–Prune-1/Wnt1 cells) showed predicted deleterious variants in genes involved in activation of the innate immune response, apoptotic pathways, DNA repair, and cell adhesion. These gene variants were also found in patients with TNBC. In detail, in human, we identified deleterious variants for six genes that are mainly involved in the activation of the innate immune response. We also found upregulation of IL-10, COL4A1, ILR1, and PDGFB, the expression levels of which are negatively correlated with prognosis in patients with TNBC. Altogether, these actions induce recruitment of TAMs in the TNBC microenvironment and promote their polarization toward anti-inflammatory/pro-tumorigenic M2-status, thus preparing the system for lung metastasis within the premetastatic niche.

Finally, we also investigated the AA7.1 to reduction of metastatic foci *in vivo*. For this purpose, MMTV–Prune-1/Wnt1 cells were injected (via the tail vein) into immunocompetent syngeneic (strain FVB) mice. At 14 days from cell injection, the mice were grouped according to their weight and AA7.1 (60 mg/kg/day, intraperitoneally) or PBS (as the vehicle negative control) were administered daily (Figure 7A). At 14 days from the start of the treatment (i.e., 28 days from cell injection), for *ex-vivo* targeting of tumorigenic cells, the mice were injected with a fluorescent imaging probe (XenoLight RediJect 2-DG-750; PerkinElmer) that targets cells with high metabolic activity in terms of glucose uptake (Figure 7A). Here, these *ex-vivo* data showed positive fluorescence signals in the lungs derived from all of the control mice (i.e., treated with PBS as vehicle). In contrast, tumorigenic cells were detected in the lungs using the *ex-vivo* imaging analysis following the AA7.1 treatment in only one of four mice (i.e., 25%), thus showing that AA7.1 can inhibit tumor cell homing to the lungs *in vivo* (Figure 7B). Hematoxylin/eosin staining of sections of lung tissue also confirmed the reductions in the metastatic foci for the AA7.1-treated mice compared with the controls (Figures 7C and 7D).

Altogether, these data indicate that pharmacological inhibition of Prune-1 through AA7.1 can reduce M2-polarization of macrophages and inhibit *in vivo* the TNBC cell homing to the lungs.

## Discussion

Despite optimal systemic chemotherapy, metastatic TNBC remains with unmet medical needs due to a lack of targeted therapies (Rakha and Chan, 2011). Metastatic progression is the result of a complex network of communication between tumorigenic and immune cells in the TME (Spano and Zollo, 2012). TAMs, dendritic cells, T and B lymphocytes, and partially differentiated myeloid progenitors (i.e., myeloid-derived suppressor cells [MDSCs]) represent the major components of the TME in TNBC (Yu and Di, 2017), mainly due to the higher propensity of TNBC cells to generate “neoantigens” due to their genomic instability and mutational burden (Bianchini et al., 2016). Once recruited into the TME by tumor-secreted soluble mediators and exosomes, the immune cells contribute to promotion and maintenance of an immunosuppressive environment, which allows immune escape and as a consequence, enhances tumor and metastatic progression (Spano and Zollo, 2012). Due to the variegated nature of the TME in TNBC, new TNBC subtypes with prognostic significance have been defined (Adams et al., 2017). Among these newly identified subtypes of TNBC stratified on the basis of the immune and metabolic markers in the TME, the subgroup of patients characterized by high levels of immunosuppressive markers/cells (M2-polarized-TAMs^High^, Tregs^High^, PDL1^High^) in a context of high glycolytic stroma (MCT4^High^) and low TILs environment are associated with poorest prognosis (Adams et al., 2017). Here, we focused on M2-TAMs; of importance, these cells were found in tumors that overexpressed Prune-1 (Figure 5D).

Genetically engineered mouse models are a useful resource in the study of the metastatic behavior of TNBC cells under immunocompetent conditions. However, GEMMs that resemble the features of metastatic TNBC have not been described to date (Pfefferle et al., 2013). Here, we developed a GEMM of metastatic TNBC driven by Prune-1 (MMTV–Prune-1/Wnt1) as a useful resource for preclinical studies to determine the efficacy of immunotherapeutic agents for treatment of established metastatic disease. We show that Prune-1 contributes to the generation of an immunosuppressive environment in both the primary tumor and the lung metastatic niches, through recruitment and polarization of M2-TAMs (Figure 2C).

Recently, in metastatic MB_group3_, we identified Prune-1-driven intracellular signaling that is involved in the metastatic behavior linked to poor prognosis (Ferrucci et al., 2018). This “metastatic axis” is guided by a protein complex formed by Prune-1 and NDPK-A (NME-1). In metastatic medulloblastoma, both of these genes were overexpressed. Here, in specific TNBC-based gene expression analyses we show higher expression levels of Prune-1 and SMAD-2/4 (downstream effectors of the TGF-β cascade). For these reasons, we confirmed the activation of TGF-β *in vivo* in mammary tumors generated in the GEMM of metastatic Prune-1-driven TNBC (i.e., MMTV–Prune-1/Wnt1 cells; Figure S6A).

Furthermore, we showed increased levels of NDPK-A (NME-1) protein phosphorylated on serine residues (i.e., Ser120, Ser122, Ser125) in primary murine Prune-1-overexpressing TNBC cells (i.e., MMTV–Prune-1/Wnt1 cells; Figure S8C). Taken altogether, these data suggest that the Prune-1/NDPK-A protein complex might act as “driver” to promote metastatic dissemination also in TNBC, through enhancement of the TGF-β cascade and downregulation of PTEN. Literature data based on NDPK-A supports the concept of its role as a suppressor of metastasis in BC (Yokdang et al., 2015; Steeg, 2003). Of interest, overexpression of Prune-1 was also shown in these tumors, which were also characterized by higher NDPK-A expression levels (Forus et al., 2001). We hypothesized that Prune-1 acts through the “metastatic axis” in TNBC, as already described for MB_group3_. An additional observation comes from the detection of extracellular NDPK-A in sera from patients with BC with metastases, and positive correlation was shown between the extracellular levels of NDPK-A and tumor growth in xenograft mice models (Yokdang et al., 2015). Of importance, extracellular Prune-1 was also detected in sera from patients with early stages of non-small-cell lung cancer (Carotenuto et al., 2014). Future efforts will be aimed at investigations into the mechanisms of actions of both of these extracellular proteins (i.e., Prune-1, NDPK-A) in metastatic TNBC, with a view to their potential prognostic value.

One additional feature is linked to a common PTEN loss observed in TNBC. This was described as its overexpression in MMTV–Wnt1 mice was reported to occur prior to tumor onset but not before metastatic behavior (Li et al., 2001). However, these findings indicated that PTEN loss and activation of Wnt signaling were not sufficient to induce metastatic spread in TNBC. These data are in agreement with PTEN downregulation in tumors that overexpress Prune-1 that was recently described for MB_group3_. This is also of importance due to the role of PI3K/AKT activation and PTEN loss in TNBC, as previously reported (Bianchini et al., 2016) and as observed here using gene-expression data from different public BC datasets, which showed that PTEN expression levels were lower in the TNBC dataset (i.e., Brown (Burstein et al., 2015)) than in other BC datasets (n = 1779, p = 7.3 × 10^−116^; data not shown). Our findings show that the Prune-1-induced metastatic axis is maintained also in TNBC, and they also suggest that Prune-1 overexpression together with PTEN loss has prognostic value to identify those patients with metastatic potential in TNBC.

Transforming growth factor β has a crucial role in the TME through modulation of the polarization status of different immune cells, including TAMs (Pickup et al., 2013). Our *in-vitro* and *in-vivo* data show that Prune-1 enhances TAM recruitment and polarization toward a pro-tumorigenic M2 status, which in turn, and together with other factors, promotes STAT3 activation and increased expression levels of Arg1, MMP-9, IL-10, and IL-1β in recipient macrophages. As summarized in Figure 7E, we hypothesize here that Prune-1 acts in metastatic TNBC through activation of intracellular tumorigenic pathways (e.g., TGF-β), release of extracellular inflammatory soluble molecules (with a preferential action of IL-17F), and changes in EV protein content, from which of the importance of Vim and syntenin-1 arise, with a role in EMT and in the homing of tumorigenic cells to lung tissues (Das et al., 2016).

However, TGF-β has also been reported to modulate the activation and status of the other components of the TME (including MDSCs, lymphocytes, neutrophils, dendritic cells, cancer-associated fibroblasts) (Pickup et al., 2013). For the above reasons, we cannot exclude the possibility that Prune-1 also affects other immune-infiltrating cells and stromal components in the TME of TNBC. This issue will be addressed in future studies.

In the present study, we also provide evidence that Prune-1 in TNBC activates ERK1/2-MAPK, through increased phosphorylation of ERK1/2 and NF-κB and through increased phosphorylation of p65 and its increased nuclear localization (Figure 5D). Of importance, the question on how Prune-1 activates the ERK1/2-MAPK and NF-κB signaling pathways should be further addressed in the future. A possible mechanism might be related to Prune-1 regulation of the Wnt signaling pathway, through its interaction with GSK-3β, and to the complex network of interactions between these signaling pathways. Cross-talk with a positive-feedback loop between the Wnt and ERK pathways has been described in tumor cells (Kim et al., 2016). Moreover, the Wnt signaling pathway induces expression of S100A4, an important player in tumor progression and metastasis, which in turn positively activates the NF-κB signaling pathway (Stein et al., 2009; Boye et al., 2008). Therefore, it might act in an autocrine manner to promote NF-κB pathway activation in Prune-1-overexpressing TNBC cells.

Studies to impair TNBC have been recently reported. We have here demonstrated *in vitro* the pharmacological inhibition of Prune-1 in BC cells using a small molecule (AA7.1) (Ferrucci et al., 2018), which affects the crosstalk between tumorigenic cells and macrophages, thus reducing the expression of M2-associated genes (Figure 6D). We also show the activity of AA7.1 *in vivo*, with the demonstration of its potential to reduce M2-TAM recruitment in the TME and to inhibit lung metastatic processes (Figures 7B and 7C) via modulation of the TGF-β pathway (Figure 6A), IL17F expression levels (Figure 6B), and exosomal protein content (i.e., Vim; Figure 6F). How the expression of Prune1 in macrophages influences their direct modulation and its inhibition by AA7.1 in time-dependent and dose-dependent manners will be an issue for future studies.

Altogether we show that AA7.1 has an important immunomodulatory property. Of note, we have previously shown that AA7.1 did not induce toxicity, through evaluation of the hematological, hepatic, and renal parameters in treated mice (Ferrucci et al., 2018). Thus, future studies will be aimed at the testing of the pharmacological inhibition of Prune-1 using AA7.1, alone or in combination with current chemotherapy regimens and/or immunotherapeutics (e.g., checkpoint inhibitors) in TNBC.

Furthermore, we have shown increased levels of IL-1β in macrophages treated with conditioned media collected from Prune-1-overexpressing TNBC cells (Figures 3B and 3C). Indeed, high levels of intracellular IL-1β have been reported for macrophages polarized toward, but not at, the M2 phenotype (Pelegrin and Surprenant, 2009). Of interest, the intracellular accumulation of IL-1β in M2-polarizing macrophages is reported to be caused by extracellular ATP-derived pyrophosphates (Pelegrin and Surprenant, 2009). For the above reasons, Prune-1 enzymatic exopolyphosphatase/pyrophosphatase activities might also be involved in the mechanisms of macrophage polarization. By taking advantage of the use of specific inhibitors of Prune-1 enzymatic activities, future studies will address these hypotheses.

Moreover, and most importantly, our *in-vitro* and *in-vivo* data here also show that Prune-1 induces an immunosuppressive TME in TNBC by releasing extracellular soluble mediators (i.e., cytokines) and vesicle-containing proteins. Among the representative cytokines, IL-17F, IL-20, and IL-28 are targets of the Prune-1-activated NF-κB signaling pathways (Otkjaer et al., 2007) (Shen et al., 2006; Osterlund et al., 2007). Once secreted, these Prune-1-induced cytokines might act on the tumor cells in an autocrine fashion or on immune cells (e.g., macrophages) in a paracrine manner within the TME. Indeed, similar to IL-17A, IL-17F activates the NF-κB and ERK1/2-MAPK signaling pathways, to promote angiogenesis and lead to upregulation of several chemokines and cytokines, thus exacerbating the inflammatory TME (Lai et al., 2011).

In the context of EV-containing proteins, among those secreted by Prune-1-overexpressing cells, we found Vim, Ifitm3, and syntenin-1 that have been reported to have roles in TNBC. Of note, Vim contributes to the aggressive phenotype and poor prognosis in TNBC (Yamashita et al., 2013). Instead, Ifitm3 was shown to be overexpressed in invasive BC, with a function related to progression and motility of TNBC cells (i.e., MDA-MB-231 cells) (Yang et al., 2013a). Most importantly, syntenin-1 is an adaptor molecule that is involved in a variety of cellular processes, including metastasis. Indeed, high expression of syntenin-1 in BC primary tumors has been significantly related to patient overall survival and progression-free survival (Yang et al., 2013b), and it is known to be negatively correlated to ER expression (Qian et al., 2013). Furthermore, syntenin-1 was shown to be overexpressed in TNBC cells with an invasive/metastatic phenotype (i.e., MDA-MB-231 cells) (Koo et al., 2002) and to have a role in promoting cell migration and invasion both *in vitro* and *in vivo*, through activation of AKT (Hwangbo et al., 2011), integrin-α1 (Yang et al., 2013a, 2013b), MAPK (Yang et al., 2013a, 2013b), and TGF-β signaling and EMT (Menezes et al., 2016), definitively promoting tumor growth and lung metastasis. Interestingly, syntenin-1 enhances the canonical TGF-β pathway (i.e., mediated by SMAD activation) and EMT, which thus leads to metastatic spread (Hwangbo et al., 2016). Lastly, the expression of syntenin-1 was shown to promote lung metastasis by influencing the inflammatory network, with the induction of inflammatory cytokines (i.e., IL-17A, IL-6) and both inflammatory and immunosuppressive cells (i.e., Th17 cells and MDSCs, respectively) in the TME of metastatic melanoma (Das et al., 2016). Overall, these EV-containing proteins represent an important finding that confirms the mechanism of communication between these TNBC cells and the immune cells within the TME.

Through WES analyses, we found mutually exclusive coding variants in the TNBC cells overexpressing Prune-1, with predicted higher/moderate impact. Among these variants, 39 gene variants were also found in the public database of human basal TNBC (COSMIC, v91) (Figure 5C). These deleterious variants are involved in activation of the innate immune response (leukocyte and macrophage activation; ANKHD1, FER1L5), cell adhesion (NEXN), apoptotic pathways (BID), and DNA repair (ERCC5) (Figure 5C). Of importance, lower expression levels of the PDE9A, Iqca1, Sirpb1b, CD244, SP140, and PIP5k1b genes was found in the BC patients with unfavorable prognosis in terms of 5-year survival analysis (dataset of Breast Invasive Carcinoma [n = 1075] from TCGA) (Figure S11). The same genes are also mutated in metastatic BC patients (dataset of metastatic BC (n = 216 (Lefebvre et al., 2016)) (Figure S11). Among these genes, CD244, Sirpb1b, and SV140 are involved in immune cell processes. CD244 has a crucial role in the activation of natural killer T cells (Mathew et al., 2009). It was defined as one of the markers of cytolytic activity in the TME of TNBC, together with the mutation status of TP53^mut^ and PIK3CA^wt^ (Cheng et al., 2020), thus acting as a marker for response to immunotherapy. On the other hand, Sirpb1b (or CD172B) shows strong correlations with immune system pathways that positively modulate the production of pro-inflammatory cytokines, including interleukin family cytokines, TNF-α, and GM-CSF (Huang et al., 2013), and participates in neutrophil trans-epithelial migration (Ribeiro et al., 2019). Of interest, the transcriptional factor SV140 is a master regulator that acts as a modulator of the adaptive immune response in BC. It was shown to be downregulated in BC cells, related to a lower infiltration of immune cells into tumor tissues and inversely correlated to relapse-free survival in BC of the basal subtype (Da Silveira et al., 2017). Importantly, SP140 is inversely correlated to NF-κB and regulates genes involved in cytokine production, inflammatory response, and cell-cell adhesion (Karaky et al., 2018). Then, Iqca1 was shown to be among the significantly downregulated genes in TNBC as compared with normal ductal cells, by genome-wide gene expression profiling analysis (Komatsu et al., 2013). Interestingly, a higher number of mutations in the PIP5K1B gene was found in metastatic BC as compared with both invasive and noninvasive BC (Durand et al., 2018). Regarding PDE9A, which is a regulator of cGMP signaling also in BC biology, it is less expressed in BC cells (including TNBC) compared with normal human mammary epithelial cells (Tinsley et al., 2009). Of importance, PDE9A was identified as a germline-related prognostic gene for ER-negative BC, involved in controlling cell growth and angiogenesis (Escala-Garcia et al., 2020).

Altogether, deleterious mutations in these genes through alterations to the innate immune response, enhancement of migratory properties, inhibition of apoptotic pathways, and impairment of the DNA repair system are potential routes to immune evasion mechanisms and metastasis formation in TNBC. Overall, these data indicate that clonal selection occurred in our animal model identify mutated genes (CD 244, Sirpb1b, SV140, Iqca1, and PIP5K1B) also found in BC human cohorts.

In conclusion, these data (as summarized in Figures 7E and 7F) show that Prune-1 drives metastatic spread in TNBC through two mechanisms of action. The first is related to its induction of migration and EMT in TNBC through activation of different intracellular signaling pathways, including TGF-β. The second mechanism of action is related to its contribution to the generation of an immunosuppressive TME that is permissive to tumor growth and metastatic progression, by taking part to the communication with TAMs and inducing their recruitment and polarization toward a tumor-promoting M2 phenotype. The interplay between Prune-1 and TAMs within the TME is mediated by modulation of the release of inflammatory cytokines and extracellular vesicles driven by Prune-1. Among the cytokines, Prune-1 enhances secretion of IL-17F, IL-28, and IL-20 from TNBC cells. We also showed that EVs derived from Prune-1-overexpressing primary metastatic TNBC cells contain proteins that have roles in EMT and metastasis (e.g., syntenin-1, Vim, Ifitm3). In human, we identified deleterious variants in six genes that are mainly involved in the activation of innate immune responses. We also used RNAseq analyses to show upregulation of IL-10, COL4A1, ILR1, and PDGFB, the expression levels of which are negatively correlated with prognosis in patients with TNBC (Figure S10). Altogether, these findings support the notion of using these genes, in the near future, as potential indicators of prognosis poor outcome.

Finally, and most importantly, these data provide hope for the use of the Prune-1-targeting drug AA7.1 and any further new small molecule derivatives as immunomodulatory agents to ameliorate metastatic dissemination also via inhibition of M2-TAM polarization. These proof-of-concept studies presented here are now ready for the definition of a route for therapeutic application in TNBC.

### Limitations of the study

The study provided preclinical data in a GEMM of metastatic TNBC, demonstrating the anti-tumorigenic action of AA7.1 small molecule. The predictive value of AA7.1 for therapeutic application in human TNBC patients deserves further investigations. At this time, the mutations here reported in the animal model generating lung metastasis with homologous mutated genes in human BC specimens need to be validated for prognostic use in human BC.

### Resource availability

The conditions of submission and the BioMed Central Copyright and License Agreement are accepted. All the information essential to interpreting the data presented are available in the Figure legends and Supplemental Information. All resources used (antibodies, cell lines, animals, and software tools) are included in the Supplemental Information.

#### Lead contact

Further information and requests for resources and reagents should be directed to and will be fulfilled by the Lead Contact, Prof. Massimo Zollo (massimo.zollo@unina.it).

#### Materials availability

All the data and materials will be available from the Lead Contact on request with a completed Materials Transfer Agreement. Mouse lines generated in this study (transgenic mouse model MMTV–Prune-1) have been deposited to European Mutant Mouse Archive (EMMA) [code: FVB-Tg(MMTV–PRUNE)/Cnrm].

#### Data and code availability

##### The accession number for the RNAseq and WES data reported in this paper are listed below. Accession to RNAseq data:

RNAseq data: Embl/EBI (Array Express): *E-MTAB-9231,* https://www.ebi.ac.uk/arrayexpress/experiments/E-MTAB-9231/

##### A ccession to WES data

Embl/EBI (ENA): S PRJEB41714 (Study ID), https://www.ebi.ac.uk/ena/browser/view/PRJEB41714

##### MMTV-Prune-1/Wnt1:

Embl/EBI (ENA): SAMEA7851609 (Sample ID), https://www.ebi.ac.uk/ena/browser/view/ERS5598758.

##### MMTV-Wnt1:

Embl/EBI (ENA): SAMEA7851608 (Sample ID), https://www.ebi.ac.uk/ena/browser/view/ERS5598759

**Mendeley deposition data for High Resolution:** Figures 2, 5, 7, S5, S6, and S12. *Mendeley Data, V1:* (URL: https://data.mendeley.com/datasets/zdmthbkcgj/draft?a=40a706bf-5521-43d3-aa45-b322921ad243) https://doi.org/10.17632/zdmthbkcgj.1, https://doi.org/10.17632/zdmthbkcgj.1.

## Methods

All methods can be found in the accompanying Transparent methods supplemental file.

## Supplemental information

Document S1. Transparent methods, Figures S1–S12, and Tables S1–S3

Data S1. RNA sequencing of macrophages, related to Figures 3A–3C RNAseq was performed on J774A.1 and Raw264 macrophages grown for 48 hr in conditioned media (collected over 24 hr) from MMTV–Prune-1/Wnt1 and MMTV–Wnt1 primary cells. Untreated macrophages were used as the negative control. Gene Symbol, Gene Name, Description of the gene, and G-FOLD values are shown.

Data S2. Proteins found into Extracellular Vesicles (EVs) derived from MMTV-Prune-1/Wnt1 cells, related to Figure 4A The extracellular proteins (analyzed using label-free quantitative mass spectrometry technology) secreted within EVs isolated from the media culture supernatant of MMTV–Prune-1/Wnt1 cells are listed. Accession number, number of distinct peptides and description of the protein are shown.

Data S3. Proteins found into Extracellular Vesicles (EVs) derived from MMTV-Wnt1 cells, related to Figure 4A The extracellular proteins (analyzed using label-free quantitative mass spectrometry technology) secreted within EVs isolated from the media culture supernatant of MMTV–Prune-1/Wnt1 cells are listed. Accession number, number of distinct peptides and description of the protein are shown.

## Footnotes

Supplemental Information can be found online at https://doi.org/10.1016/j.isci.2020.101938.
