# Supplementary material for: Prune-1 drives polarization of tumor-associated macrophages (TAMs) within the lung metastatic niche in triple-negative breast cancer
Source: iScience. 2020 Dec 13;24(1):101938. doi: 10.1016/j.isci.2020.101938 (PMC7779777; doi:10.1016/j.isci.2020.101938)
Supplement: Document S1. Transparent methods, Figures S1–S12, and Tables S1–S3 [file mmc1.pdf]

iScience, Volume *24*

Supplemental Information

Prune-1 drives polarization of tumor-associated macrophages (TAMs) within the lung metastatic niche in triple-negative breast cancer

Veronica Ferrucci, Fatemeh Asadzadeh, Francesca Collina, Roberto Siciliano, Angelo Boccia, Laura Marrone, Daniela Spano, Marianeve Carotenuto, Cristina Maria Chiarolla, Daniela De Martino, Gennaro De Vita, Alessandra Macrì, Luisa Dassi, Jonathan Vandenbussche, Natascia Marino, Monica Cantile, Giovanni Paolella, Francesco D'Andrea, Maurizio di Bonito, Kris Gevaert, and Massimo Zollo

Supplemental Information

Prune-1 drives polarization of tumor-associated macrophages (TAMs) within the lung metastatic niche in triple-negative breast cancer

Veronica Ferrucci,[1,2,3] Fatemeh Asadzadeh,[1,2,#] Francesca Collina,[4,#] Roberto Siciliano,[1] Angelo Boccia,[1] Laura Marrone,[1,2] Daniela Spano,[1] Marianeve Carotenuto,[2] Cristina Maria Chiarolla,[1] Daniela De Martino, [2] Gennaro De Vita, [2] Alessandra Macrì,[1] Luisa Dassi,[1] Jonathan Vandenbussche,[5] Natascia Marino,[1,6] Cantile Monica,[4] Giovanni Paolella,[1] Francesco D'Andrea,[7] Maurizio di Bonito,[4] Kris Gevaert,[5] and Massimo Zollo[1,2,3,8,9 *]

[1] CEINGE, Biotecnologie Avanzate, Naples, 80145, Italy

[2] Dipartimento di Medicina Molecolare e Biotecnologie Mediche (DMMBM), 'Federico II' University of Naples, Naples, 80134, Italy

[3] European School of Molecular Medicine (SEMM), University of Milan, Milan, Italy

[4] Pathology Unit, Istituto Nazionale Tumori-IRCS- Fondazione G.Pascale, Naples, 80131, Italy

[5] VIB-UGent Centre for Medical Biotechnology, Ghent, 9052, Belgium

[6] Department of Medicine, Indiana University-Purdue University Indianapolis, Indianapolis, 46202, USA

[7] Dipartimento di Sanità pubblica – AOU, Università degli Studi di Napoli Federico II, Naples, 80131, Italy

[8] DAI Medicina di Laboratorio e Trasfusionale, AOU Federico II, Naples, 80131, Italy

[9] Lead Contact

[#] These authors contributed equally to this study

[*] Correspondence: massimo.zollo@unina.it (M.Z.)

Supplemental Information

Supplemental Figures and legends

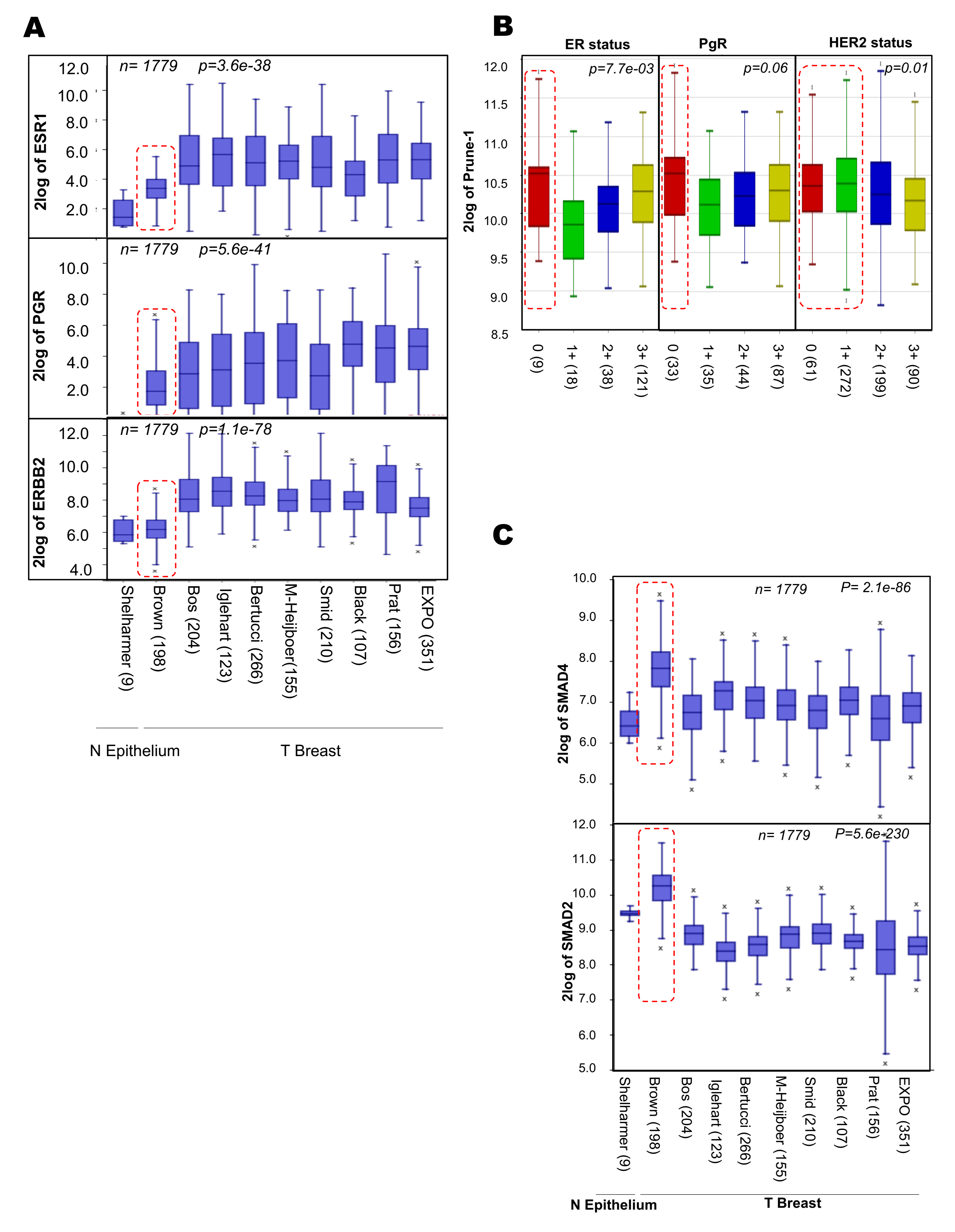

Supplementary Figure 1

Figure S1. Related to Figure 1A. Prune-1 mRNA levels are negatively correlated to ER, PgR and HER2 status.

(A) RNA log2 expression analysis of ER (*i.e.,* ESR1), PgR, and HER2 (*i.e.,* ERBB2) levels of primary BC samples across different publically available datasets, compared with normal epithelium (N Epithelium; Shelharmer dataset only). Data from 10 independent public-domain breast cancer gene-expression datasets confirm lower ER, PgR, and HER2 expression levels in TNBC samples (*i.e.,* Brown; red dashed line) (n = 1779; P = $3.0e^{-169}$). ER, estrogen receptor; PgR, progesterone receptor; HERBB2 or HER2, human epidermal growth factor receptor 2. **(B)** RNA log2 expression of Prune-1 across primary BC samples grouped according to their ER, PgR, and HER2 status (stratified from 0 to 3+ score, as evaluated by IHC) in the publically available dataset of the Tumor Breast Invasive Carcinoma, with gene expression data acquired from The Cancer Genome Atlas (n = 1097). Higher Prune-1 expression levels are seen for BC samples with negative scores for both ER and PgR, and in those with scores ranging from 0 to 1+ for HER2 status (red dashed lines). ER, estrogen receptor; PgR, progesterone receptor; HER2, human epidermal growth factor receptor 2. **(C)** Overexpression of SMAD2 and SMAD4 in TNBC. RNA log2 expression analysis of SMAD2 (top) and SMAD4 (bottom) levels for primary BC samples across the different publically available datasets (T Breast), compared with normal epithelium (N Epithelium). Data from 10 independent public-domain breast cancer gene-expression datasets are shown. There was overexpression of both SMAD2 and SMAD4 in TNBC samples (*i.e.,* Brown [Burstein et al., 2015] n = 1779; SMAD2: P = 5.6e-230; SMAD4: P = 2.1e-86.

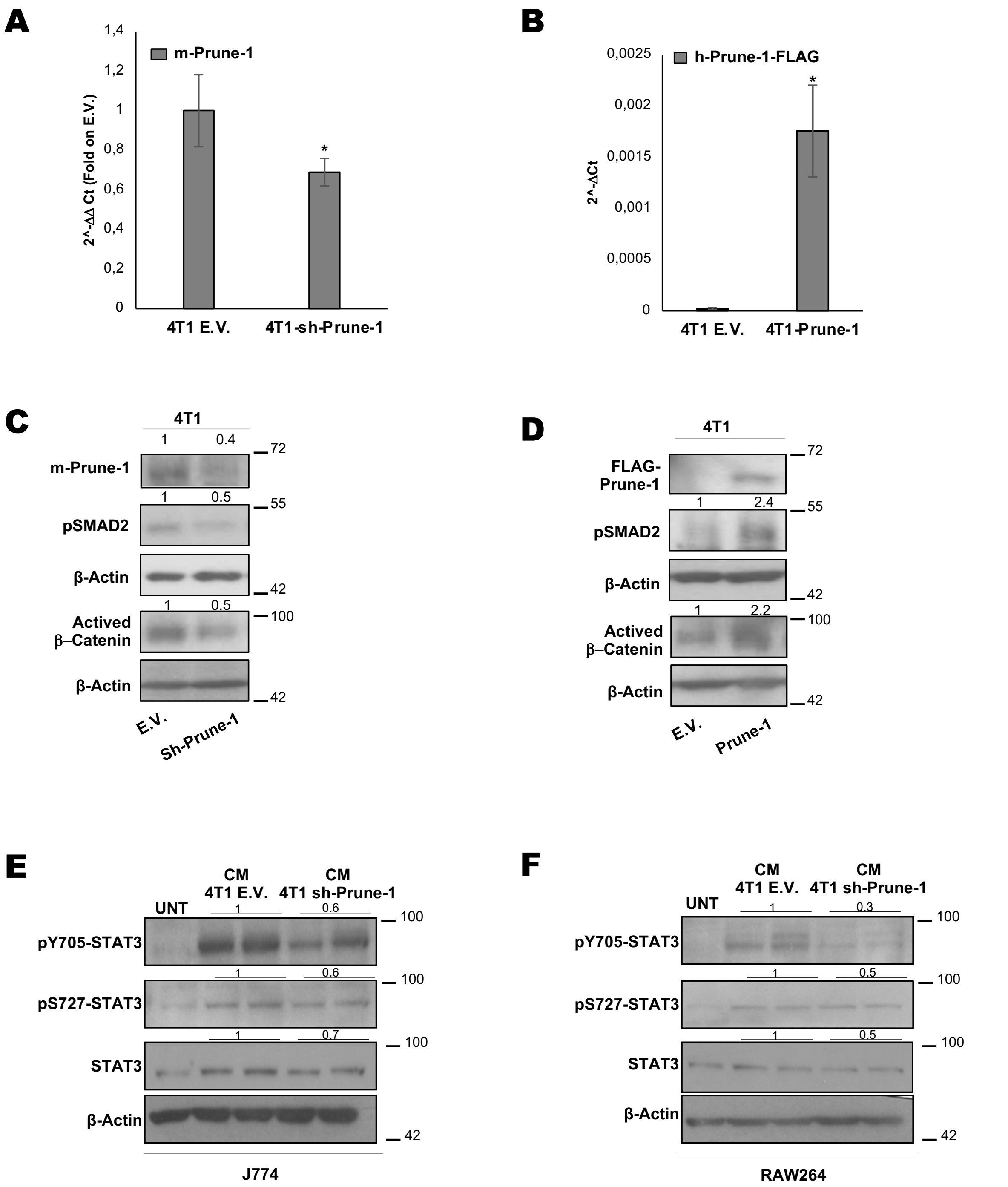

Supplementary Figure 2

Supplementary Figure S2. Related to Figure 1B-E. Prune-1 at the interplay of communication between murine TNBC cells (4T1) and macrophages.

(A) Real-time PCR analysis of murine Prune-1 (m-Prune-1) in 4T1 down-regulated clones. The down-regulation levels of m-Prune-1 is shown as fold (0.6) on E.V. 4T1 clones. Error bars indicate standard deviation (SD) of the mean of three independent cell clones. *, $P<0.05$ in Student's t-test compared to Empty Vector control clones. **(B)** Mean relative expression (ΔCt) of human Prune-1 (h-Prune-1) from three independent stable 4T1 overexpressing cell clones measured using real-time PCR analysis; Empty vector (E.V.) 4T1 clones were used as negative controls. Data are represented as mean +/- SD. *, $P<0.05$ in Student's t-test compared to Empty Vector control clones. **(C, D)** Immunoblotting for Prune-1–silenced **(C)** and Prune-1–overexpressing **(D)** 4T1 stable clones. Empty vector (E.V.) 4T1 clones were used as the negative control. Down-regulation and up-regulation of phospho-Ser467-SMAD2 and activated β-catenin, respectively, are seen in Prune-1–silenced cells (0.5-fold) **(C)** and Prune-1–overexpressing cells (phospho-Ser467-SMAD2: 2.4-fold; activated β-catenin: 2.2-fold) **(D)**, respectively. Densitometer analyses are also shown. β-Actin levels were used as the loading control. **(E, F)** Immunoblotting for the indicated proteins in J774 **(E)** and Raw264 **(F)** macrophages grown for 30 min in conditioned media from Prune-1–silenced and control 4T1 clones are shown. Empty vector (E.V.) 4T1 clones and untreated (UNT) macrophages were used as the negative controls. β-Actin levels were used as the loading control.

All experiments were performed in triplicate. All data are expressed as the mean ± standard deviation. *, $P < 0.05$ in Student's t-test.

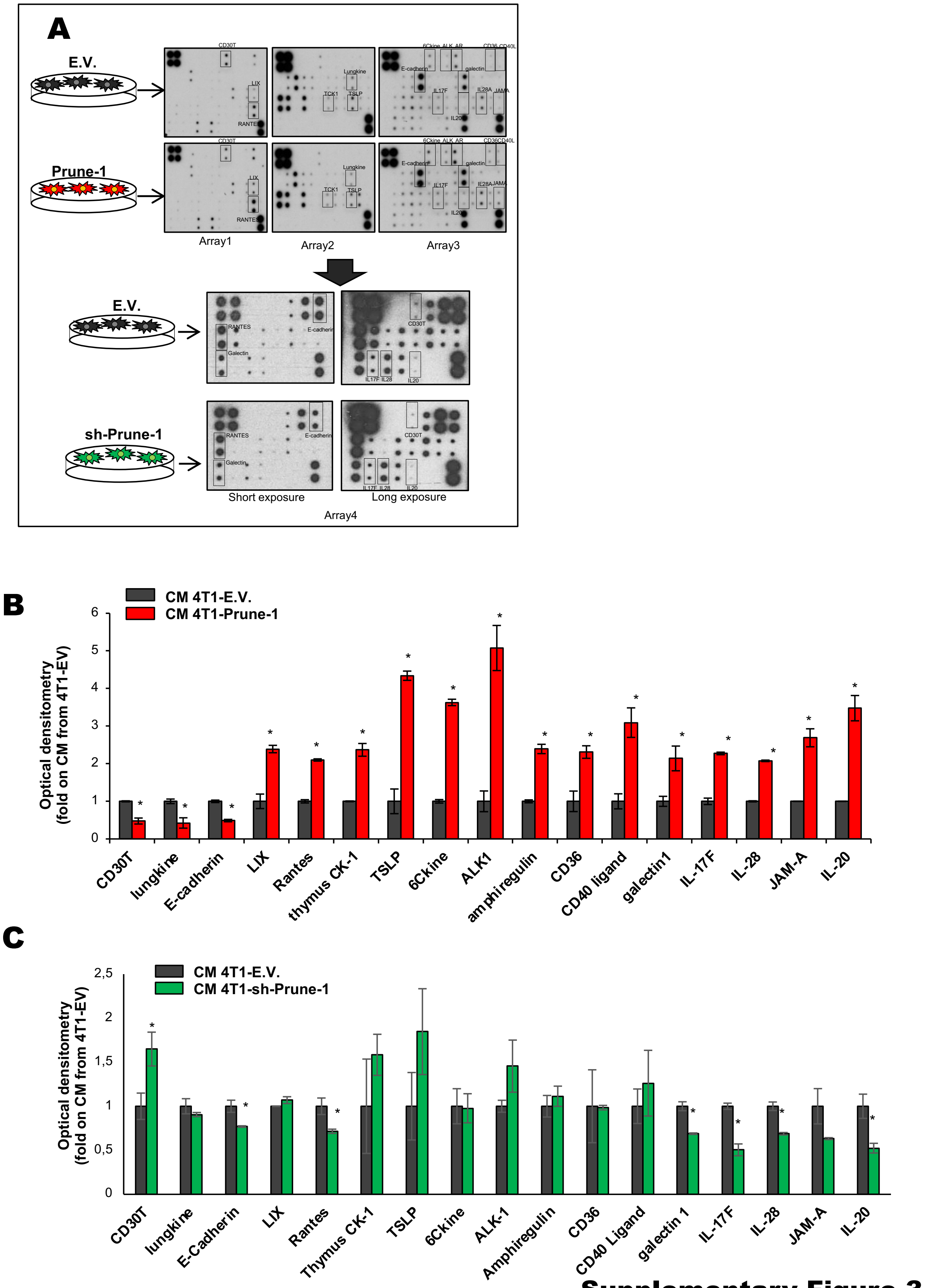

Supplementary Figure 3

Supplementary Figure S3. Related to Figure 1F. Secretion of inflammatory cytokines from TNBC cells (4T1) are modulated by Prune-1.

(A) Upper panel: Mouse cytokine antibody arrays incubated with pooled conditioned media (CM) from Prune-1–overexpressing and empty vector (E.V.) 4T1 cell clones. **Bottom panel:** Mouse cytokine antibody array was performed to determine expression levels of 17 cytokines (as previously found significantly up-regulated and down-regulated by Prune-1) in conditioned media (CM) from Prune-1–silenced 4T1 cell clones. **(B, C)** Fold-induction of cytokines in the conditioned media from 4T1-overexpressing **(B)** or 4T1-silenced **(C)** cell clones on empty vector clones. All experiments were performed in triplicate. All data are expressed as the mean ± standard deviation. *, $P<0.05$ in Student's t-test compared to cytokines levels in conditioned media of 4T1 Empty Vector control clones.

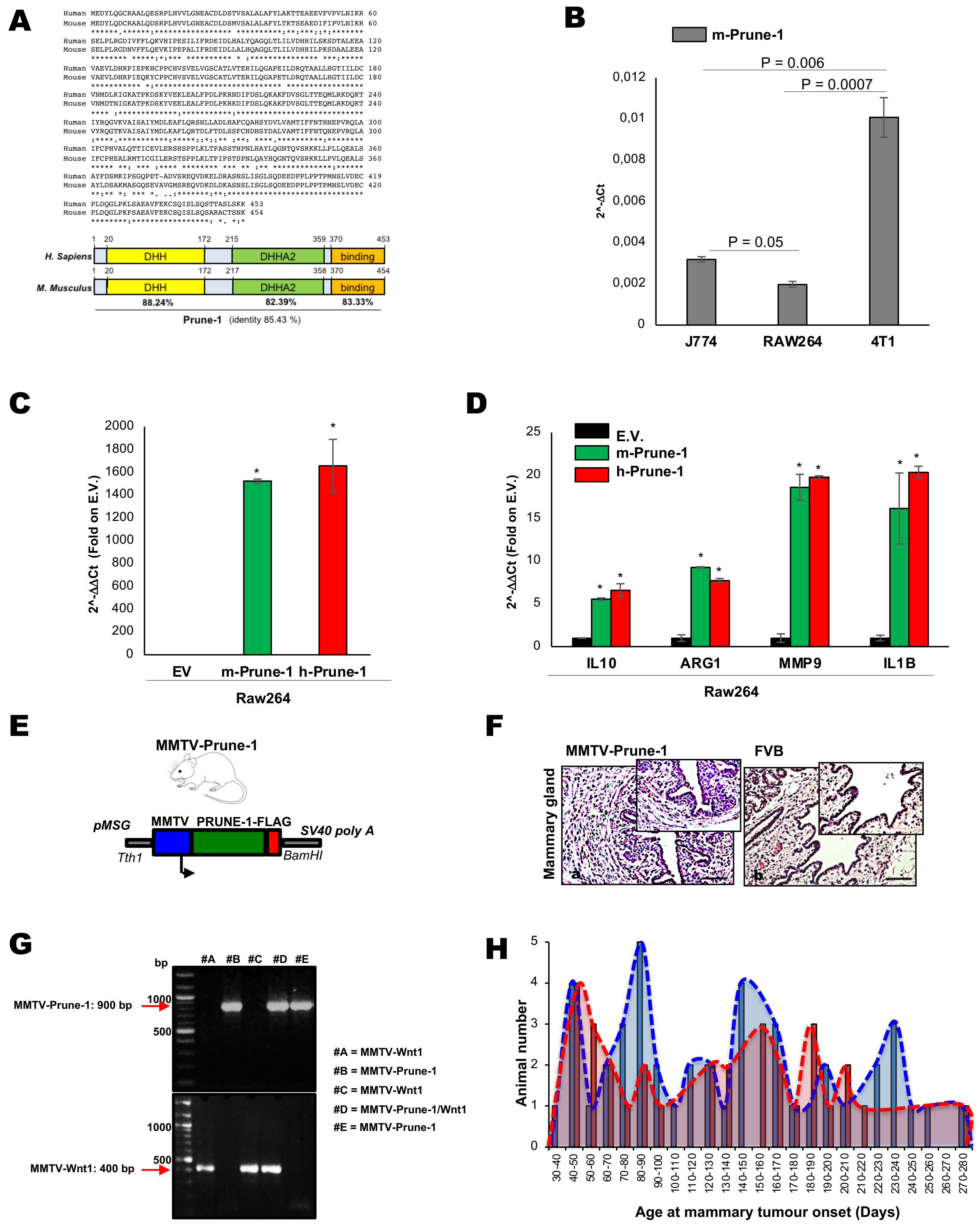

Supplementary Figure 4

Supplementary Figure S4. Related to Figure 2A-B. A genetically engineered mouse model overexpressing Prune-1 in mammary gland.

(A) Sequence homology analyses between human (h) and murine (m) Prune-1 protein sequences show significant similarity (overall aa: 85.43% identity; DHH and DHHA2 enzymatic domains: 88.24% and 82.39% identity, respectively; NME1 binding domain: 83.33% identity. These sequence alignments were realized using the ClustalX 2.1 software. **(B)** Real-time PCR analysis showing relative expression (ΔCt) of endogenous murine Prune-1 (m-Prune-1) in J774A.1, Raw264 and 4T1 cells. **(C, D)** Real-time PCR analysis of Raw264 transiently transfected for 48 h with human (h) or murine (m) Prune-1 cDNA plasmid constructs, showing the fold-expression of m-Prune and h-Prune-1 **(C)**, and the inflammatory cytokines **(D)**, as compared to the empty-vector-transfected cells. Data are means ± standard deviation. *, $P<0.05$ in Student's t-test compared to empty-vector-transfected cells. **(E, F)** Schematic diagram of plasmid pMSG-MMTV-LTR-Prune-1-FLAG used for the generation of the MMTV–Prune-1 transgenic mouse model. The pMSG vector (Pharmacia Biotech Sevrage, Uppsala, Sweden) contains the Mouse mammary tumor virus (MMTV) long terminal repeat upstream of a polylinker. The human Prune-1 cDNA, containing the complete protein coding region with the FLAG tag fused in-frame at the carboxyl-terminus terminal was cloned into polylinker sites of pMSG in sense orientation relative to the MMTV long terminal repeat and the downstream SV-40 early promoter. The resultant construct was designated pMSG-MMTV-Prune-1-FLAG **(E)**. Representative hematoxylin-eosin sections of normal mammary gland from wild-type female FVB mice (80 days) **(a)** compared with mammary hyperplasia developed from female MMTV–Prune-1 transgenic mice (80 days). **(b)** **(F)** Magnification, 40×. Scale bar: 20 μm. **(G)** PCR-based analysis for genotyping of the mice. Top: genomic tail DNA samples from mice were amplified using specific primers against transgenes MMTV–Prune-1 (900 bp) or MMTV–Wnt1 (500 bp). **(H)** Age-standardised mammary tumor incidence rate in female virgin MMTV–Prune-1/Wnt1 and MMTV–Wnt1 mice. Prune-1 expression in mammary glands of MMTV–Prune-1/Wnt1 mice (n = 31) does not alter breast tumor onset compared to MMTV–Wnt1 mice (n = 44). Mice were monitored from birth up to breast tumor onset, at up to 280 days.

All experiments were performed in triplicate. All data are expressed as the mean ± standard deviation. *, $P < 0.05$ in Student's t-test.

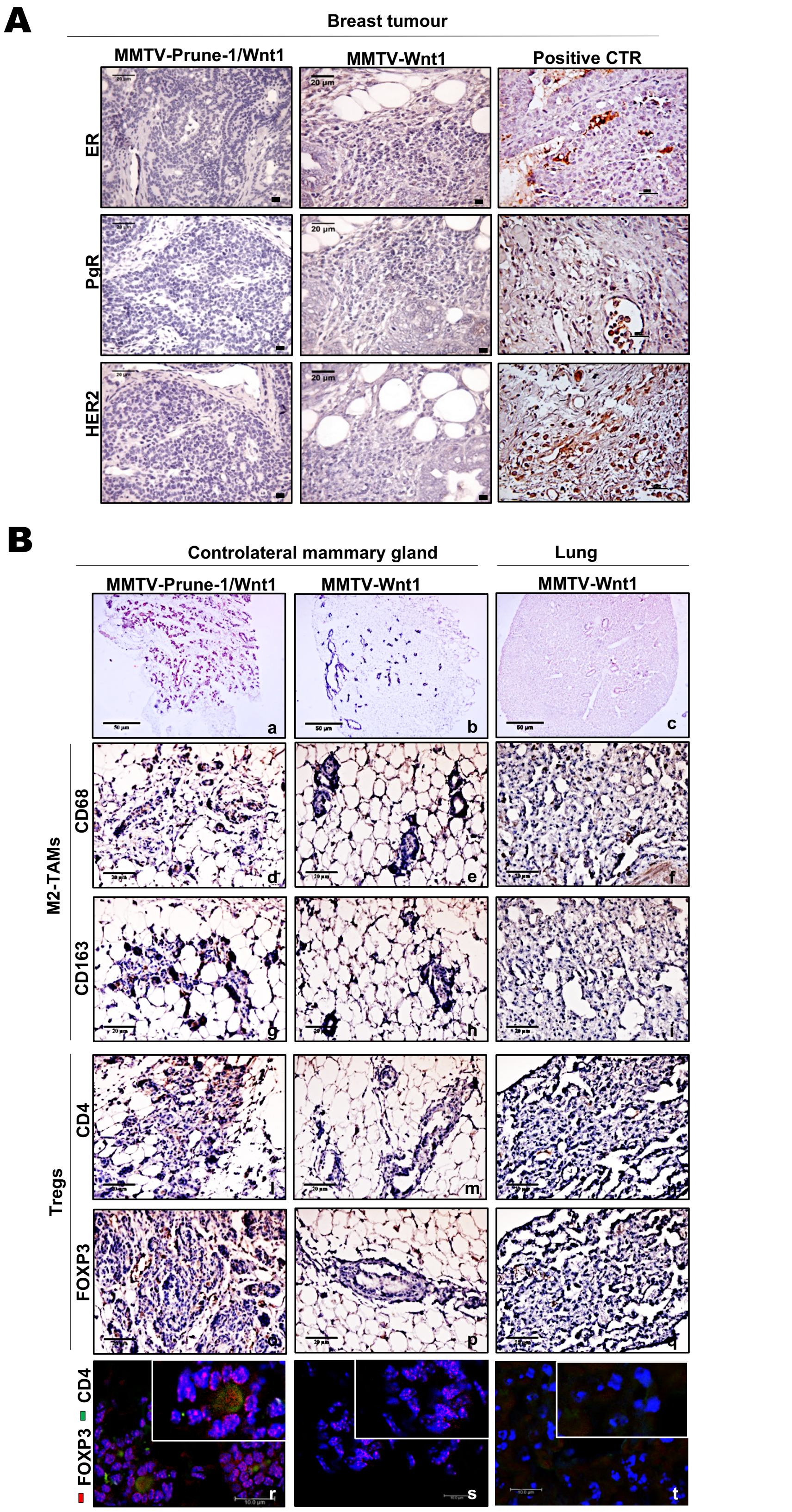

Supplementary Figure 5

Supplementary Figure S5. Related to Figure 2C. A genetically engineered mouse model of metastatic TNBC overexpressing Prune-1 in mammary gland.

(A) Representative immunohistochemistry (IHC) staining on sections of mammary tumors developed from double transgenic MMTV–Prune-1/Wnt1 and MMTV–Wnt1 mice for antibodies as indicated (*i.e.,* ER, PgR, HER2). Undetectable levels of both ER and PgR (*i.e.,* ER^{-}, PgR^{-}, $HER2^{-}$) are shown, compared to the positive control on the right. Magnification, 40×; Scale bar: 20 μm. **(B)** Representative hematoxylin-eosin staining **(a-c)**, IHC **(d-q)** and immunofluorescence (IF; **r-t**) performed on sections of contralateral mammary gland of MMTV–Prune-1/Wnt1 and MMTV–Wnt1 mice and lung from MMTV–Wnt1 mice using antibodies against the following: CD68 **(d-f)** and CD163 **(g-i)**, as markers for M2-TAMs; CD4 **(l-n)**, as a marker for T cells; and FOXP3 **(o-q)**, as a marker for Tregs. Double indirect IF was performed to detect Tregs (*i.e.,* CD4+ FOXP3+, **r-t**). CD4: green; FOXP3: red; DAPI: blue. Acquisitions were performed using a quantitative pathology workstation (Mantra) with image analysis software (Inform). Magnification, 5x, 40×. Scale bar: 50 μm, hematoxylin-eosin; 20 μm, IHC; 10 μm, IF.

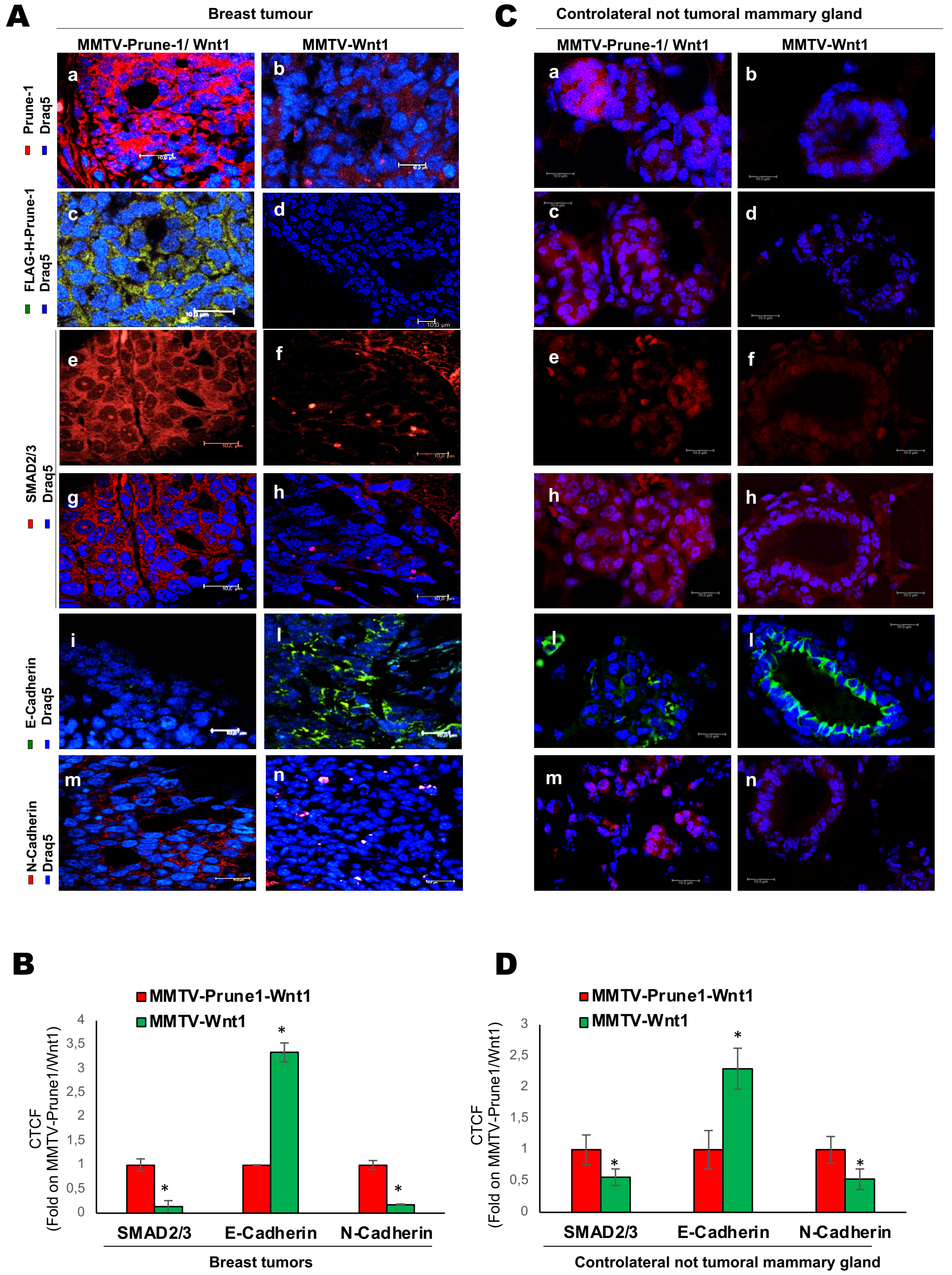

Supplementary Figure 6

Supplementary Figure S6. Related to Figure 2C. Prune-1 enhances TGF-β pathway and EMT process in mammary tumorigenesis of a genetically engineered mouse model of metastatic TNBC.

(A-D) Representative immunofluorescence staining for antibodies, as indicated in mammary tumors **(A)** and contralateral nontumoral mammary gland **(C)** from MMTV–Prune-1/Wnt1 and MMTV–Wnt1 mice. Endogenous Prune-1 (**a-b**), FLAG-tagged-human-Prune-1 (**c-d**), SMAD2-3 (**e-h**), E-cadherin (**i-l**) and N-cadherin (**m-n**). Draq5 was used for nuclear staining (blue). Scale bar: 10 μm. The intensity of the staining for each antibody was measured in each cell, normalized compared to MMTV–Prune-1/Wnt1, and expressed as fold-increase/ decrease **(B, D)**. More than 100 cells were counted. CTCF, corrected total cell fluorescence. Data are means ± standard deviation. *, $P<0.05$ in Student's t-test compared to MMTV-Prune-1/Wnt1.

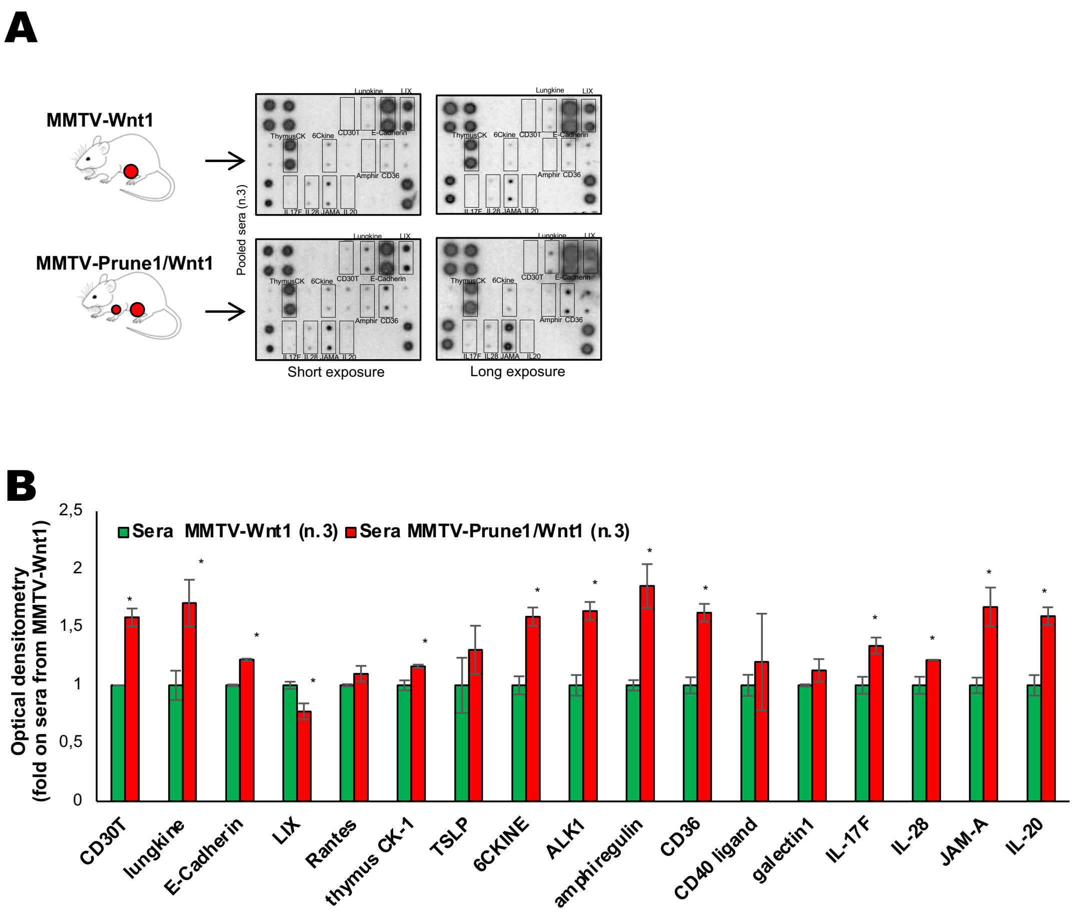

Supplementary Figure 7

Supplementary Figure S7. Related to Figure 2D. Secretion of inflammatory cytokines in sera of a GEMM of metastatic TNBC overexpressing Prune-1.

(A) Mouse cytokine antibody array was performed to determine expression levels of 17 cytokines (shown previously significantly up-regulated and down-regulated by Prune-1) in sera collected and pooled from MMTV–Prune-1/Wnt1 (n=3) and MMTV–Wnt1 (n=3) mice. **(B)** Fold-induction of cytokines in the conditioned media from sera collected and pooled from MMTV–Prune-1/Wnt1 (n=3) and MMTV–Wnt1 (n=3) mice. Data are means ± standard deviation. *, P<0.05 in Student's t-test compared to sera from MMTV-Wnt1 mice.

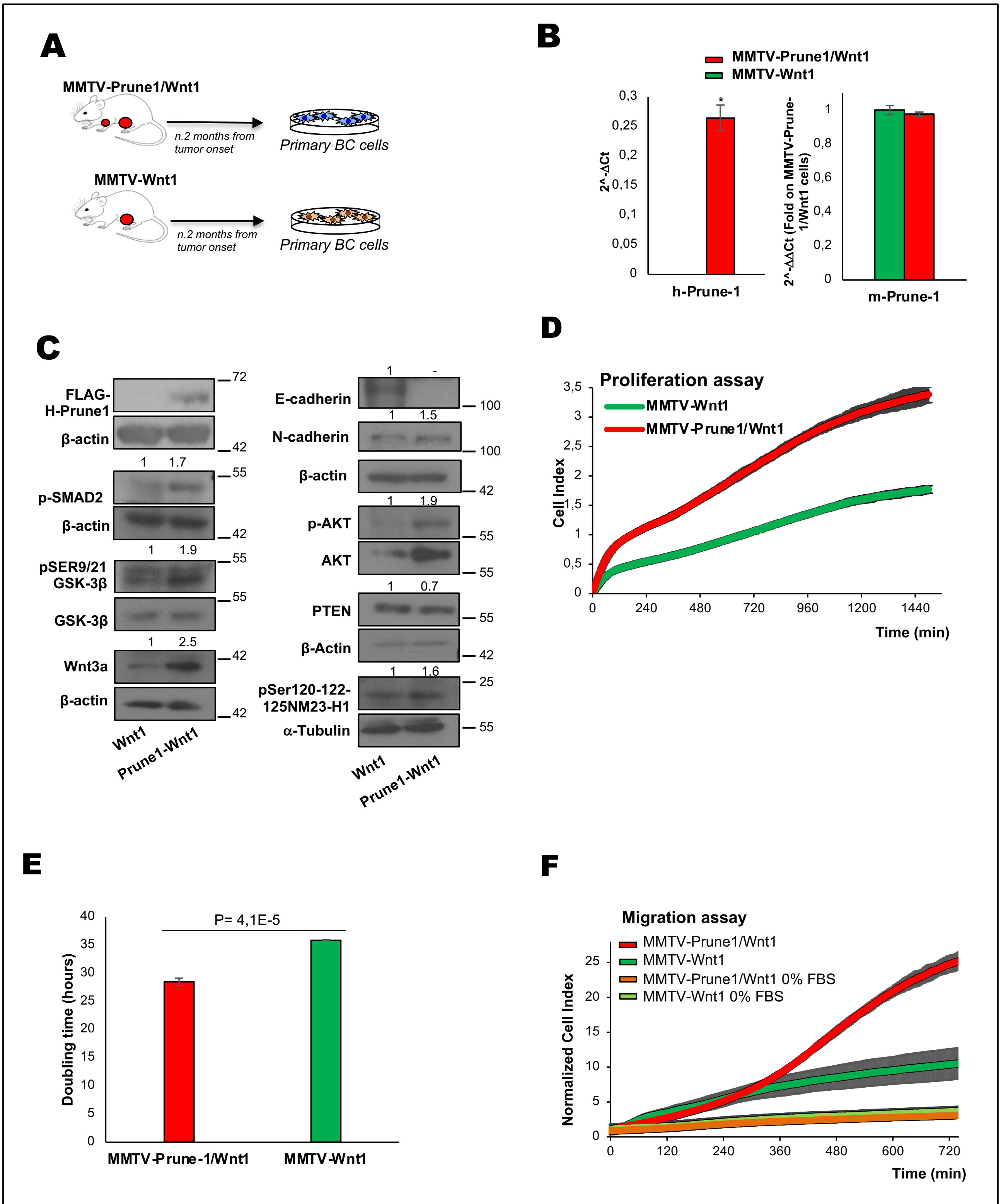

Supplementary Figure 8

Supplementary Figure S8. Related to Figure 3A. Prune-1 enhances migratory properties in primary cells obtained from GEMM of metastatic TNBC.

(A) Schematic diagram showing primary cells obtained from the tumors generated from MMTV–Prune-1/Wnt1 and MMTV–Wnt1 mice at 2 months from tumor onset. **(B)** Real-time PCR analysis showing the expression levels of transgene human and endogenous murine Prune-1 (*i.e.,* h-Prune-1, m-Prune-1) in primary cells obtained from MMTV–Prune-1/Wnt1 and MMTV–Wnt1 tumors. *, $P<0.05$ in Student's t-test compared to MMTV–Wnt1. **(C)** Representative immunoblotting for antibodies as indicated from MMTV–Prune-1/Wnt1 and MMTV–Wnt1 cells. **(D)** Cell Index as a measure of cell proliferation of MMTV–Prune-1/Wnt1 (red) and MMTV–Wnt1 (green) cells (xCELLigence RTCA software). Data are means ±standard deviation of triplicate samples. **(E)** Doubling time of MMTV–Prune-1/Wnt1 (28.4 h, red) and MMTV–Wnt1 (35.8 h, green) cells grown up to 120 h (xCELLigence RTCA software). Data are means ±standard deviation of triplicate samples. **(F)** Cell Index as a measure of cell migration of MMTV–Prune-1/Wnt1 (red) and MMTV–Wnt1 (green) cells (xCELLigence RTCA software). Migration kinetics were monitored in response to 10% fetal bovine serum (FBS; red, green) and to 0% FBS (brown, light green) as negative controls. MMTV–Prune-1/Wnt1 cells (red) show increased migration in response to the 10% FBS gradient, compared with MMTV–Wnt1 cells (green). Cell migration driven by 0% FBS gradient was used as the negative control (MMTV–Prune-1/Wnt1 cells, orange; MMTV–Wnt1 cells, light green). Data are means ±standard deviation of triplicate samples.

All experiments were performed in triplicate. All data are expressed as the mean ± standard deviation. *, $P < 0.05$.

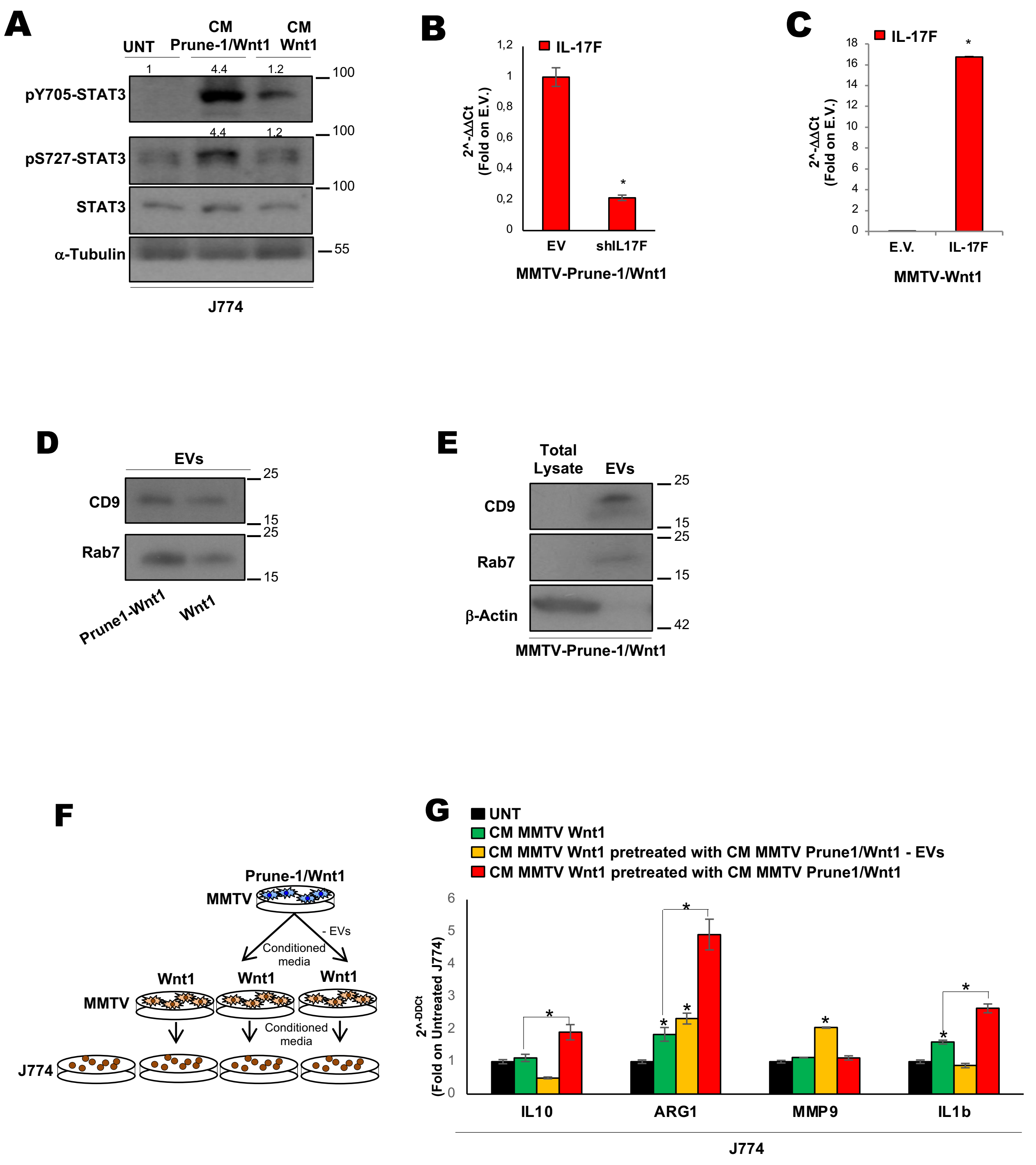

Supplementary Figure 9

Supplementary Figure S9. Related to Figures 3-4. Prune-1 induces macrophage polarization toward M2 phenotype via IL-17F and extracellular vesicles.

(A) Immunoblotting for the indicated proteins in J774 macrophages starved for 6 h and then stimulated for 30 min in conditioned media collected from MMTV–Prune-1/Wnt1 and MMTV–Wnt1 cells. Densitometer analyses for the proteins are shown. α-Tubulin was used as the loading control. **(B)** Real-time PCR analysis of murine IL-17F in MMTV–Prune-1/Wnt1 cells transfected with sh-IL-17F or empty vector (E.V.) control. *, $P<0.05$ in Student's t-test compared to empty vector transfected cells. **(C)** Real-time PCR analysis of murine IL-17F in MMTV–Wnt1 cells transfected with murine IL-17F or EV control. *, $P<0.05$ in Student's t-test compared to empty vector transfected cells. **(D)** Immunoblotting for the indicated proteins in extracellular vesicles (EVs) isolated from media culture supernatant of MMTV–Prune-1/Wnt1 or MMTV–Wnt1 cells. **(E)** Immunoblotting for the indicated proteins in total lysate or in EVs isolated from the conditioned media collected from MMTV–Prune-1/Wnt1 cells. **(F, G)** Schematic representation of experimental design. J774 macrophages were grown for 48 h in conditioned media collected (after 24 h) from MMTV–Wnt1 cells that had been previously grown for 24 h in the conditioned media collected from MMTV–Prune-1-Wnt cells depleted or not in EVs. J774 macrophages were untreated or grown in conditioned media collected (after 24 h) from MMTV–Wnt1 cells, and used as negative and positive controls, respectively **(F)**. Real-time PCR analysis of some M2-associated genes, including IL-10, Arg-1, MMP-9, and IL-1β, in J774 macrophages grown for 48 h in conditioned media collected (after 24 h) from MMTV–Wnt1 cells that had been previously grown for 24 h in conditioned media collected from MMTV–Prune-1-Wnt cells depleted (yellow) or not (red) in EVs. **(G)**. J774 macrophages untreated (black) or grown in conditioned media from MMTV–Wnt1 cells (green) were used as negative and positive controls, respectively. *, $P<0.05$ in Student's t-test compared to untreated macrophages, or, where indicated, compared to macrophages grown in conditioned media from MMTV-Wnt1 cells.

All experiments were performed in triplicate. All data are expressed as the mean ± standard deviation. *, $P < 0.05$, in Student's t-test

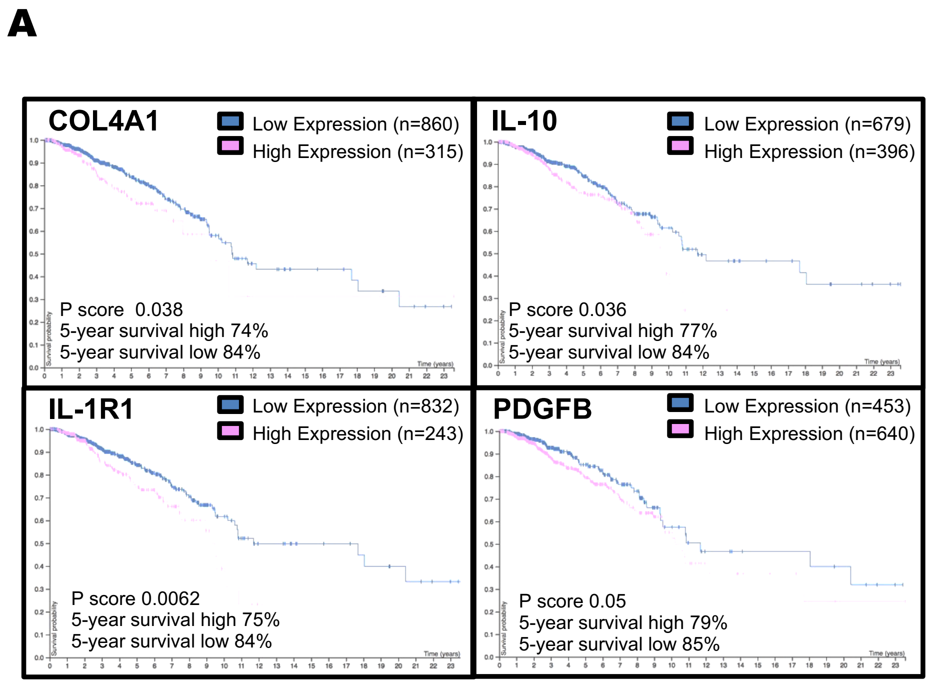

Supplemental Figure 10

Supplementary Figure S10. Related to Figure 5B. “Core genes” identified in macrophages positively correlated with poor prognosis in BC.

(A) Analyses of survival data obtained from the publically available dataset of Breast Invasive Carcinoma (n=1075) from The Cancer Genome Atlas (TCGA). Of interest, we found that high expression of the IL-10, COL4A1, ILR1, and PDGFB genes was associated with decreased 5-year survival in patients with TNBC.

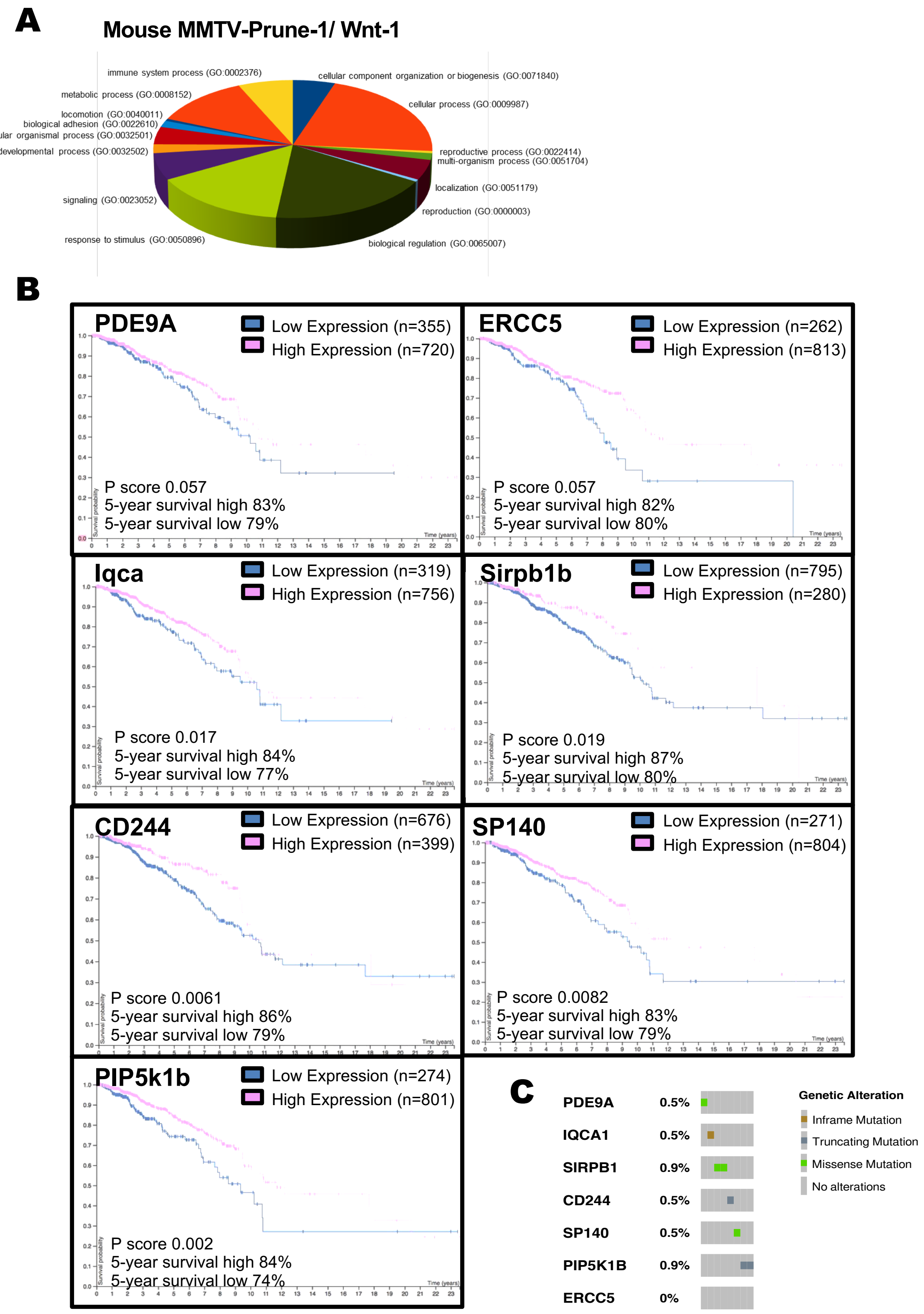

Supplementary Figure 11

Supplementary Figure S11. Related to Figure 5C. Mutation spectra in primary murine TNBC cells that overexpressed Prune-1.

(A) Pie chart illustrating Gene Ontology (GO) analysis of deleterious variants mutually exclusively found in MMTV–Prune-1/Wnt1 cells (as compared to MMTV–Wnt1 cells). **(B)** Analyses of survival data obtained from the publically available dataset of Breast Invasive Carcinoma (n=1075) from The Cancer Genome Atlas (TCGA). Of interest, we found that low expression of the PDE9A, ERCC5, Iqca, Sirpb1b, CD244, SP140, and PIP5k1b genes was associated with decreased 5-year survival in patients with TNBC. **(C)** Frequency of genetic alterations in the PDE9A, ERCC5, Iqca, Sirpb1b, CD244, SP140, and PIP5k1b genes in the publically available dataset of metastatic BC (n=216). Altogether, these show genetic alterations with a frequency of 3.7% (Cbioportal for cancer genomics; https://www.cbioportal.org)

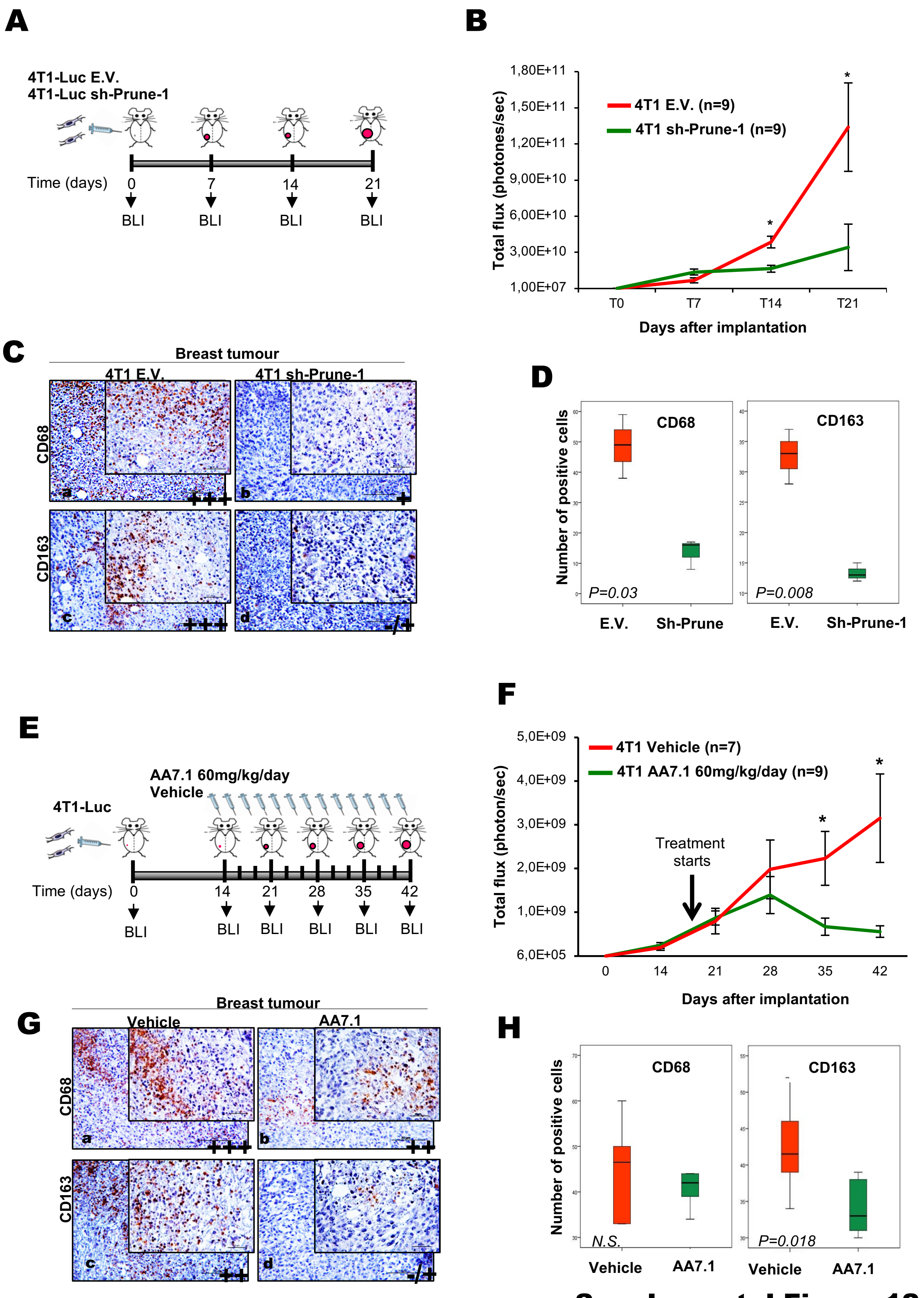

Supplemental Figure 12

Supplementary Figure S12. Related to Figures 6-7. Silencing or pharmacological inhibition of Prune-1 reduces TNBC growth *in vivo*.

(A-D) Prune-1 down-regulation impairs *in-vivo* primary tumor growth of TNBC by inhibition of TAM recruitment and polarization toward an M2-phenotype. **(A)** Representative orthotopic xenograft procedure using Prune-1–silenced 4T1 cells stably expressing the firefly luciferase gene (4T1-LUC cells) and the empty vector (E.V.) as the control, and then implanted into the mammary fat pads of 18 immunocompetent BalbC mice (nine mice per group). The mice were imaged every 7 days using *in-vivo* bioluminescent imaging (IVIS Spectrum) to monitor tumor growth from time of implantation (T0) to 21 days (T21) after tumor implantation. **(B)** Tumor growth (quantified photon emission; photon/s) from the region of interest of mice orthotopically injected with 4T1-LUC stable clones. Differences in total fluxes from the two groups of mice at 14 and 21 days from tumor implantation show impairment of tumor growth *in vivo* in mice implanted with Prune-1–silenced 4T1-LUC cell clones. *, $P<0.05$ in Student's t-test compared to mice orthotopically injected with 4T1-LUC stable clones**.** **(C)** Representative immunohistochemistry of paraffin-embedded tumors generated by implanting 4T1-LUC cells into mammary fat pads of BalbC mice, using the indicated antibodies. Magnification, 20×, 40×. Scale bars: 20 μm, 10 μm. **(D)** Box plot (SPSS software) showing cells positive for CD68 and CD163 expression within primary tumors from mice implanted with Prune-1–silenced (4T1–Sh-Prune-1) and empty vector (E.V.) 4T1 cell clones. Infiltrating M2-TAMs ($CD68^+$ $CD163^+$ cells) were evaluated using three different tumors from each group of mice. CD68: $P = 0.03$; CD163: $P = 0.008$. **(E-H)** Pharmacological inhibition of Prune-1 affects *in-vivo* primary tumor growth in TNBC through impairment of M2-TAM polarization in the TME. **(E)** Representative orthotopic xenografts using 4T1 cells stably expressing the firefly luciferase gene (4T1-LUC cells), and then implanted into the mammary fat pads of 16 immunocompetent BalbC mice. After tumors were established (14 days after implantation), the mice received intraperitoneal administration of AA7.1 60 mg/kg daily or PBS (vehicle control). Tumorigenesis was followed *in vivo* using bioluminescent imaging (IVIS Spectrum), from time of implantation (T0) to 42 days (T42) after implantation. **(F)** Tumor growth according to quantified photon emission (photon/s) from the region of interest of mice orthotopically injected with 4T1-LUC stable clones. The differences in total flux from the two groups of mice after 21 and 28 days from treatment (T35, T42 from tumor implantation, respectively) showed significant impairment of tumor growth *in vivo* in mice treated with AA7.1. *, $P<0.05$ in Student's t-test compared to vehicle-treated mice as controls. **(G)** Representative immunohistochemistry of paraffin-embedded tumors generated by implantation of 4T1-LUC cells into mammary fat pads of BalbC mice, using indicated antibodies. Magnification, 20×, 40×. Scale bars: 20 μm, 10 μm. **(H)** Box plot (SPSS software) showing cells positive for CD68 and CD163 expression within primary tumors

from mice implanted with 4T1-LUC cells treated with AA7.1 or PBS, as vehicle control. Infiltrating M2-polarized TAMs ($CD68^{+}$ $CD163^{+}$ cells) evaluated using three different tumors from each group of mice. CD68: $P = 0.9$; CD163: $P = 0.018$.

Table S1. Related to Figure 1A and Figure S1C. Gene expression correlation analyses between Prune-1, SMAD2 and SMAD4 in Breast Cancer samples stratified according to their Estrogens/Progesteron receptor status. PgR, Progesteron receptor; ER, Estrogen receptor; HER2, Human epidermal growth factor 2. The data were obtained from "R2-Genomic and Visualization Platform" using "Tumor Breast Invasive Carcinoma" dataset containing gene expression data acquired from publicly accessible Cancer Genome Atlas (TCGA) breast cancer cohort (n=1097 breast cancer samples logged in TCGA).

PgR, ER, HER2 status	Variable 1	Variable 2	r-value	p-value
**PgR negative (n=344)**	**PRUNE-1**	**SMAD2**	0.35	$2.3e^{-11}$
		**SMAD4**	0.328	$4.6e^{-10}$
**ER negative (n=238)**	**PRUNE-1**	**SMAD2**	0.35	$2.8e^{-08}$
		**SMAD4**	0.308	$1.3e^{-06}$
**HER2 negative (n=563)**	**PRUNE-1**	**SMAD2**	0.209	$5.3e^{-07}$
		**SMAD4**	0.193	$4.1e^{-06}$

Table S2: Related to Figure 2B. Incidence of lung metastases in MMTV-Prune1/Wnt1 and MMTV-Wnt1 mice. MMTV–Wnt1 mice did not develop lung metastasis at 2 months from tumor onset, while MMTV–Prune-1/Wnt1 mice showed macro-metastasis in the lungs at 97% penetrance.

Genotype	Genetic background	ID	Tumour growth timing	Lung metastases
**MMTV-Wnt1**	FVB	432	2 months	NO
**MMTV-Wnt1**	FVB	446	2 months	NO
**MMTV-Wnt1**	FVB	Z1-C21	2 months	NO
**MMTV-Wnt1**	FVB	573	2 months	NO
**MMTV-Wnt1**	FVB	502	2 months	NO
**MMTV-Wnt1**	FVB	Z1-C226	2 months	NO
**MMTV-Wnt1**	FVB	43	2 months	NO
**MMTV-Wnt1**	FVB	19	2 months	NO
**MMTV-Prune1/Wnt1**	FVB	409	2 months	**YES**
**MMTV-Prune1/Wnt1**	FVB	389	2 months	NO
**MMTV-Prune1/Wnt1**	FVB	444	2 months	**YES**
**MMTV-Prune1/Wnt1**	FVB	428	1 month 20 days	**YES**
**MMTV-Prune1/Wnt1**	FVB	521	2 months	NO
**MMTV-Prune1/Wnt1**	FVB	476	2 months	**YES**
**MMTV-Prune1/Wnt1**	FVB	493	2 months	**YES**
**MMTV-Prune1/Wnt1**	FVB	Z1-C231	3 months	**YES**
**MMTV-Prune1/Wnt1**	FVB	772	2 months	**YES**
**MMTV-Prune1/Wnt1**	FVB	821	2 months	**YES**
**MMTV-Prune1/Wnt1**	FVB	Z1-802	2 months	**YES**

Table S3: Related to Figure 3B-C. M2-associated genes found up-regulated in J774A.1 and/ or Raw264 grown in conditioned media collected from MMTV-Prune-1/Wnt1 cells, as compared to untreated macrophages.

Gene symbol	Protein function (GO)
**AF251705**	CMRF35-like molecule; Acts as an activating receptor in mast cells and macrophages
**Abtb2**	Ankyrin repeat and BTB/POZ domain-containing protein 2; May be involved in the initiation of hepatocyte growth
**Ahr**	Aryl hydrocarbon receptor; Ligand-activated transcriptional activator. Binds to the XRE promoter region of genes it activates. Activates the expression of multiple phase I and II xenobiotic chemical metabolizing enzyme genes (such as the CYP1A1 gene). Mediates biochemical and toxic effects of halogenated aromatic hydrocarbons. Involved in cell-cycle regulation. Likely to play an important role in the development and maturation of many tissues. Regulates the circadian clock by inhibiting the basal and circadian expression of the core circadian component PER1.
**Aqp9**	Aquaporin-9; Forms a channel with a broad specificity. Mediates passage of a wide variety of non-charged solutes including carbamides, polyols, purines, and pyrimidines in a phloretin- and mercury-sensitive manner, whereas amino acids, cyclic sugars, Na(+), K(+), Cl(-), and deprotonated monocarboxylates are excluded.
**Arg1**	Arginase-1; Key element of the urea cycle converting L-arginine to urea and L-ornithine, which is further metabolized into metabolites proline and polyamides that drive collagen synthesis and bioenergetic pathways critical for cell proliferation, respectively; the urea cycle takes place primarily in the liver and, to a lesser extent, in the kidneys.
**Arl4c**	ADP-ribosylation factor-like protein 4C; Small GTP-binding protein which cycles between an inactive GDP-bound and an active GTP-bound form, and the rate of cycling is regulated by guanine nucleotide exchange factors (GEF) and GTPase-activating proteins (GAP). GTP-binding protein that does not act as an allosteric activator of the cholera toxin catalytic subunit. May be involved in transport between a perinuclear compartment and the plasma membrane, apparently linked to the ABCA1-mediated cholesterol secretion pathway.
**Bhlhe40**	Class E basic helix-loop-helix protein 40; Transcriptional repressor involved in the regulation of the circadian rhythm by negatively regulating the activity of the clock genes and clock-controlled genes. Acts as the negative limb of a novel autoregulatory feedback loop (DEC loop) which differs from the one formed by the PER and CRY transcriptional repressors (PER/CRY loop). Both these loops are interlocked as it represses the expression of PER1/2 and in turn is repressed by PER1/2 and CRY1/2.
**Ccl12**	C-C motif chemokine 12; Chemotactic factor that attracts eosinophils, monocytes, and lymphocytes but not neutrophils. Potent monocyte active chemokine that signals through CCR2. Involved in allergic inflammation and the host response to pathogens and may play a pivotal role during early stages of allergic lung inflammation; Belongs to the intercrine beta (chemokine CC) family
**Ccl22**	C-C motif chemokine 22; Chemotactic for activated T-lymphocytes. May play an important role in the collaboration of dendritic cells and B- lymphocytes with T-cells in immune responses
**Ccl6**	C-C motif chemokine 6; CCL6(22-95) and CCL6(23-95) are potent chemoattractants; Belongs to the intercrine beta (chemokine CC) family
**Ccl7**	C-C motif chemokine 7; Chemotactic factor that attracts monocytes and eosinophils, but not neutrophils. Augments monocyte anti-tumor activity
**Cd36**	Platelet glycoprotein 4; Multifunctional glycoprotein that acts as receptor for a broad range of ligands. Ligands can be of proteinaceous nature like thrombospondin, fibronectin, collagen or amyloid-beta as well as of lipidic nature such as oxidized low-density lipoprotein (oxLDL), anionic phospholipids, long-chain fatty acids and bacterial diacylated lipopeptides. They are generally multivalent and can therefore engage multiple receptors simultaneously, the resulting formation of CD36 clusters initiates signal transduction and internalization of receptor-ligand complexes.
**Cish**	Cytokine-inducible SH2-containing protein; SOCS family proteins form part of a classical negative feedback system that regulates cytokine signal transduction. CIS is involved in the negative regulation of cytokines that signal through the JAK-STAT5 pathway such as erythropoietin, prolactin and interleukin 3 (IL3) receptor. Inhibits STAT5 trans-activation by suppressing its tyrosine phosphorylation.
**Edn1**	Endothelin-1; Endothelins are endothelium-derived vasoconstrictor peptides; Belongs to the endothelin/sarafotoxin family.
**Egr1**	Early growth response protein 1; Transcriptional regulator. Recognizes and binds to the DNA sequence 5'-GCG(T/G)GGGCG-3'(EGR-site) in the promoter region of target genes. Binds double-stranded target DNA, irrespective of the cytosine methylation status (By similarity). Regulates the transcription of numerous target genes, and thereby plays an important role in regulating the response to growth factors, DNA damage, and ischemia. Plays a role in the regulation of cell survival, proliferation and cell death. Activates expression of p53/TP53 and TGFB1.
**Egr2**	E3 SUMO-protein ligase EGR2; Sequence-specific DNA-binding transcription factor. Binds to two specific DNA sites located in the promoter region of HOXA4. Binds to the promoter region of ERBB2. May play a role in the regulation of hindbrain segmentation, might act in combination with the Hox network to specify odd and even rhombomeres, and might participate in the control of the expression of some of the homeobox containing genes; Belongs to the EGR C2H2-type zinc-finger protein family.
**Emp1**	Epithelial membrane protein 1
**Emp2**	Epithelial membrane protein 2; Functions as a key regulator of cell membrane composition by regulating proteins surface expression. Also, plays a role in regulation of processes including cell migration, cell proliferation, cell contraction and cell adhesion. Negatively regulates caveolae formation by reducing CAV1 expression and CAV1 amount by increasing lysosomal degradation. Facilitates surface trafficking and the formation of lipid rafts bearing GPI-anchor proteins. Regulates surface expression of MHC1 and ICAM1 proteins increasing susceptibility to T-cell mediated cytotoxicity.
**F2rl2**	Proteinase-activated receptor 3; High affinity receptor for activated thrombin coupled to G proteins that stimulate phosphoinositide hydrolysis. May play a role in platelets activation; Belongs to the G-protein coupled receptor 1 family.

**Fabp4**	Fatty acid-binding protein, adipocyte; Lipid transport protein in adipocytes. Binds both long chain fatty acids and retinoic acid. Delivers long-chain fatty acids and retinoic acid to their cognate receptors in the nucleus; Belongs to the calycin superfamily. Fatty-acid binding protein (FABP) family.
**Fam198b**	Protein FAM198B; Family with sequence similarity 198, member B; Belongs to the FAM198 family.
**Flrt3**	Leucine-rich repeat transmembrane protein FLRT3; Functions in cell-cell adhesion, cell migration and axon guidance, exerting an attractive or repulsive role depending on its interaction partners. Plays a role in the spatial organization of brain neurons. Plays a role in vascular development in the retina. Plays a role in cell-cell adhesion via its interaction with ADGRL3 and probably also other latrophilins that are expressed at the surface of adjacent cells. Interaction with the intracellular domain of ROBO1 mediates axon attraction towards cells expressing NTN1.
**Frmd6**	FERM domain containing 6.
**Gnb4**	Guanine nucleotide-binding protein subunit beta-4; Guanine nucleotide-binding proteins (G proteins) are involved as a modulator or transducer in various transmembrane signaling systems. The beta and gamma chains are required for the GTPase activity, for replacement of GDP by GTP, and for G protein- effector interaction
**H2-Eb1**	Histocompatibility 2, class II antigen E beta; Belongs to the MHC class II family.
**Hbegf**	Proheparin-binding EGF-like growth factor; Growth factor that mediates its effects via EGFR, ERBB2 and ERBB4. Required for normal cardiac valve formation and normal heart function. Promotes smooth muscle cell proliferation. May be involved in macrophage-mediated cellular proliferation. It is mitogenic for fibroblasts, but not endothelial cells. It is able to bind EGF receptor/EGFR with higher affinity than EGF itself and is a far more potent mitogen for smooth muscle cells than EGF. Also acts as a diphtheria toxin receptor
**Il10**	Interleukin-10; Inhibits the synthesis of a number of cytokines, including IFN-gamma, IL-2, IL-3, TNF and GM-CSF produced by activated macrophages and by helper T-cells.
**Il1b**	Interleukin-1 beta; Potent proinflammatory cytokine. Initially discovered as the major endogenous pyrogen, induces prostaglandin synthesis, neutrophil influx and activation, T-cell activation and cytokine production, B-cell activation and antibody production, and fibroblast proliferation and collagen production; Belongs to the IL-1 family.
**Lyzl4**	Lysozyme-like protein 4; May be involved in fertilization. Has no detectable bacteriolytic in vitro. Has no lysozyme activity in vitro.
**Mmp12**	Macrophage metalloelastase; May be involved in tissue injury and remodeling. Has significant elastolytic activity. Can accept large and small amino acids at the P1' site, but has a preference for leucine. Aromatic or hydrophobic residues are preferred at the P1 site, with small hydrophobic residues (preferably alanine) occupying P3.
**Mmp9**	Matrix metalloproteinase-9; Could play a role in bone osteoclastic resorption. Cleaves type IV and type V collagen into large C-terminal three quarter fragments and shorter N-terminal one quarter fragments.
**Myc**	Myc proto-oncogene protein; Transcription factor that binds DNA in a non-specific manner, yet also specifically recognizes the core sequence 5'- CAC[GA]TG-3'. Activates the transcription of growth-related genes. Binds to the VEGFA promoter, promoting VEGFA production and subsequent sprouting angiogenesis.
**Myrf**	Myelin regulatory factor; Myelin regulatory factor: Constitutes a precursor of the transcription factor. Mediates the autocatalytic cleavage that releases the Myelin regulatory factor, N-terminal component that specifically activates transcription of central nervous system (CNS) myelin genes; Belongs to the MRF family.
**Olr1**	Oxidized low-density lipoprotein receptor 1; Receptor that mediates the recognition, internalization and degradation of oxidatively modified low density lipoprotein (oxLDL) by vascular endothelial cells. OxLDL is a marker of atherosclerosis that induces vascular endothelial cell activation and dysfunction, resulting in pro-inflammatory responses, pro- oxidative conditions and apoptosis. Its association with oxLDL induces the activation of NF-kappa-B through an increased production of intracellular reactive oxygen and a variety of pro- atherogenic cellular responses.
**Padi2**	Protein-arginine deiminase type-2; Catalyzes the deimination of arginine residues of proteins; Belongs to the protein arginine deiminase family.
**Plbd1**	Phospholipase B-like 1; Exhibits weak phospholipase activity, acting on various phospholipids, including phosphatidylcholine, phosphatidylinositol, phosphatidylethanolamine and lysophospholipids. However, in view of the small size of the putative binding pocket, it has been proposed that it may act rather as an amidase or a peptidase (By similarity)
**Ppbp**	Chemokine (C-X-C motif) ligand 7, isoform CRA_b; Pro-platelet basic protein.
**Ptgs1**	Prostaglandin G/H synthase 1; Converts arachidonate to prostaglandin H2 (PGH2), a committed step in prostanoid synthesis. Involved in the constitutive production of prostanoids in particular in the stomach and platelets. In gastric epithelial cells, it is a key step in the generation of prostaglandins, such as prostaglandin E2 (PGE2), which plays an important role in cytoprotection. In platelets, it is involved in the generation of thromboxane A2 (TXA2), which promotes platelet activation and aggregation, vasoconstriction and proliferation of vascular smooth muscle cells.
**Socs2**	Suppressor of cytokine signaling 2; SOCS family proteins form part of a classical negative feedback system that regulates cytokine signal transduction. SOCS2 appears to be a negative regulator in the growth hormone/IGF1 signaling pathway. Probable substrate recognition component of a SCF-like ECS (Elongin BC-CUL2/5-SOCS-box protein) E3 ubiquitin ligase complex which mediates the ubiquitination and subsequent proteasomal degradation of target proteins.
**Syn1**	Synapsin-1; Neuronal phosphoprotein that coats synaptic vesicles, binds to the cytoskeleton, and is believed to function in the regulation of neurotransmitter release. Regulation of neurotransmitter release. The complex formed with NOS1 and CAPON proteins is necessary for specific nitric-oxide functions at a presynaptic level.
**Trib1**	Tribbles homolog 1; Adapter protein involved in protein degradation by interacting with RFWD2/COP1 ubiquitin ligase. Promotes CEBPA degradation and inhibits its function. Controls macrophage, eosinophil and neutrophil differentiation via the COP1-binding domain. Regulates myeloid cell differentiation by altering the expression of CEBPA in a COP1- dependent manner. Interacts with MAPK kinases and regulates activation of MAP kinases, but has no kinase activity (By similarity); Belongs to the protein kinase superfamily. CAMK Ser/Thr protein kinase family.

Supplemental Information
TRANSPARENT METHODS

Cell proliferation, migration, co-culture transwell assays and doubling time using Cell Index technology

MMTV–Prune-1/Wnt1 and MMTV–Wnt1 cells were harvested, washed with PBS, resuspended to 2 $\times 10^5$ cells/mL in high-glucose Dulbecco's modified Eagle's medium (Euroclone) without fetal bovine serum (FBS), and aliquoted into single wells of plates (xCELLigence CIM plate 16; #05665817001; Acea Biosciences). Cell migration was driven by a 10% FBS gradient, with 0% FBS used as the negative control. Measurements were taken at 2-min intervals, as impedance changes across the electrodes at the bottoms of the wells, for 12 h.

The effects of conditioned media from Prune-1–overexpressing, Prune-1–silenced, and empty-vector 4T1 cell clones were investigated on macrophage (J774A.1, Raw264.7) migration using a real-time XCelligence System Analysis instrument (ACEA Biosciences). Briefly, 1 $\times 10^6$ 4T1 cells of each clone were resuspended in 10 mL complete RPMI 1640 medium and plated into 10-cm-diameter plates. After 24 h, the conditioned media were collected and put into the lower chambers as the chemoattractant in the cell motility assays. Here, 2.5 $\times 10^4$ macrophages were seeded into the upper chambers, in complete RPMI 1640 medium. Three replicates were used for each experimental point. The Cell Index was automatically determined every 2 min, for 12 h. For each experimental point, the mean ±standard deviation of the Cell Index was calculated. Two independent sets of experiments were performed.

The co-culture transwell migration assay with MMTV–Prune-1/Wnt1 cells and J774A.1 macrophages were performed using a real-time XCelligence System Analysis instrument (ACEA Biosciences). Briefly, J774A.1 macrophages were starved for 6 h, then grown for 48 h in conditioned media collected from vehicle- or AA7.1-treated MMTV–Prune-1/Wnt1 cells, or from MMTV–Wnt1 cells. Then, MMTV–Prune-1/Wnt1 cells (2.5 $\times 10^5$) were harvested, washed with PBS, resuspended in high-glucose Dulbecco's modified Eagle's medium (Euroclone) without FBS and aliquoted into single wells of plates (xCELLigence CIM plate 16; #05665817001; Acea Biosciences). Macrophages (1 $\times 10^5$ cells) were plated into single plates of "RTCA E-Plate Inserts" at the top of the microplates. Untreated macrophages were used as negative controls. Cell migration was driven by a 2% FBS gradient. Measurements were taken at 5-min intervals, as impedance changes across the electrodes at the bottoms of the wells, for 8 h.

For cell proliferation assays, MMTV–Prune-1/Wnt1 and MMTV–Wnt1 cells were harvested, washed with PBS, resuspended in high-glucose Dulbecco's modified Eagle's medium (Euroclone)

with 10% FBS and aliquoted (1 $\times 10^4$ cells) into single wells of plates (xCELLigence E-plate 16; Acea Biosciences). Cell proliferation was recorded at 2-min intervals, as impedance changes across the electrodes at the bottoms of the wells, for up to 24 h.

For doubling time measurements, MMTV–Prune-1/Wnt1 and MMTV–Wnt1 cells were harvested, washed with PBS, resuspended (5 $\times 10^3$ cells) in high-glucose Dulbecco's modified Eagle's medium (Euroclone) with 10% FBS, and seeded into single wells of plates (xCELLigence E-plate 16; Acea Biosciences). Cell proliferation was recorded at 2-min intervals, as impedance changes across the electrodes at the bottoms of the wells, for up to 120 h. The doubling time was calculated using the RTCA software, from the logarithmic phase of the growth curves as a readout of cell proliferation and behavior that integrates changes in cell number, attachment, and morphology.

Coculture experiments

The effects of conditioned media from Prune-1–silenced, empty-vector 4T1 cell clones, and MMTV–Prune-1/Wnt1 and MMTV–Wnt1 cells were analyzed for activation of J774A.1 macrophages. Briefly 1 $\times 10^6$ 4T1 cells of each clone were resuspended in 10 mL complete RPMI 1640 or DMEM medium, plated into 10-cm-diameter plates, and grown at 37 °C for 24 h. Then the conditioned media were collected. One day before culture in the conditioned media, J774A.1 and Raw264.7 macrophages were plated into 10-cm-diameter plates. At the time of the culturing in conditioned media, macrophages were at 50% confluence, and after 6 h of starvation in serum-free medium, the macrophages were grown in conditioned media for 30 min (for Western blotting) or 48 h (for real-time PCR). Each experimental point was carried out in duplicate. After the culturing in the conditioned media, the macrophages were washed with PBS, collected, and used for protein and/or RNA extraction.

Cytokine antibody array

The conditioned media from three different Prune-1–overexpressing, Prune-1–silenced, and empty-vector 4T1 cell clones were pooled. In the same manner, the sera from three different MMTV–Prune-1/Wnt1 and MMTV–Wnt1 cells were also collected and pooled. The relative levels of cytokines in the pooled conditioned media and murine sera were measured (RayBio Mouse Cytokine Antibody Array C, series 2000; Prodotti Gianni), according to the manufacturer protocol. Densitometric analysis was carried out using the Quantity One software (BioRad). Expression levels were normalized to the levels of the positive control spots (contained within the membrane). For each cytokine, the mean ±standard error was determined.

In-vivo mouse experiments

Mouse experiments were approved by the Institutional Animal Care and Ethical Committee of CEINGE 'Federico II' University of Naples (Protocol 29, September 30, 2012; *Dipartimento Sanità Pubblica Veterinaria* D.L. 116/92). The transgenic mouse model MMTV–Prune-1 (Strain ID: EM:09937; code: FVB-Tg(MMTV-PRUNE)/Cnrm) has been archived to The European Mutant Mouse Archive (EMMA).

Extracellular vesicles isolation

Extracellular vesicles were purified from media culture supernatants of MMTV–Prune-1/Wnt1 and MMTV–Wnt1 cells through methodology previously described (Thery et al., 2006), with modifications. Briefly, MMTV–Prune-1/Wnt1 and MMTV–Wnt1 cells were grown in 'exosomes-depleted medium' (obtained via overnight centrifugation at 100,000 ×g) until they reached 80% confluence. After 48 h, the conditioned medium was collected by centrifugation at 300× *g* for 10 min. The supernatant was then pre-cleared to remove cells, dead cells, and cellular debris by centrigufation at 2,000× *g* for 20 min. The extracellular vesicles were then obtained via ultracentrifugation at 100,000× *g* for 70 min. After washing with PBS, the extracellular vesicles were further purified (ExoQuick Exosome Precipitation Solution; Cat.#EXOQ5A-1; System Biosciences) by incubation for 12 h and centrifugation at 1,500× *g* for 60 min.

Mutation analyses

Whole exome sequencing was performed on tumorigenic cells obtained from primary tumors developed in MMTV–Prune-1-Wnt1 and MMTV–Wnt1 mice. The samples were prepared according to the preparation guidelines for the SureSelect Target Enrichment kits (SureSelect Mouse Capture kits; Agilent). The libraries were sequenced with an Illumina HiSeq sequencer platform. The mapping reference was *mm10* from UCSC (original GRCm38 from NCBI, December 2011), and the annotation database dbSNP version 142.

For the MMTV–Prune-1-Wnt1 sample, for the post-alignment statistics, the initial mappable reads (*i.e.,* number of mapped reads to mouse genome) was 74,311,106, the non-redundant reads (*i.e.,* number of de-duplicate reads from Picard tools version picard-tools-1.118) 68,415,279, the on-target reads (*i.e.,* number of reads mapped to target regions) 45,504,404, the on-target yield (*i.e.,* the sum of the bases in the final alignment to the target regions) 5,298,453,340 bp and the mean depth of target regions (*i.e.,* {on-target yield} / {Target regions}) 107.3 X.

For the MMTV–Wnt1 sample, for the post-alignment statistics, the initial mappable reads was 81,372,126, the non-redundant reads 74,412,189, the on-target reads 50,202,591, the on-target yield 5,880,337,790 bp, and the mean depth of target regions 119.1 X.

The mutually exclusive variants of the MMTV–Prune-1-Wnt1 sample were selected by using the *Ablebitis* tool. These mutations were filtered by excluding: (i) the synonymous, (ii) the noncoding exon variants, (iii) those mapping within intronic and the intergenic regions, (iv) the in-frame deletions and insertions, (v) the upstream or downstream gene variants, and (vi) the 3' and 5' UTR region. Then the variants annotated with putative impact/ deleteriousness as 'low' were excluded. Only the 'coding' variants (that had passed all filters) with a putative impact/ deleteriousness as 'high, modifier or moderate' were analysed through Ensembl Variant Effect Predictor to determine the effects of the selected variants on the protein sequence. Finally, the variants predicted as 'tolerated' by Sorting Intolerant From Tolerant, version 5.2.2, (GRCh38) were also excluded.

Survival data analyses

The analyses of the survival data were obtained from the publicity available dataset of Breast Invasive Carcinoma (n=1075) from TCGA. In detail, the patients were classified into two expression groups (based on the 'fragments per kilobase million' value of each gene), and the correlation between expression levels and patient survival was examined. The survival outcomes of the two groups were compared by log-rank tests.

RNAseq analyses

Library construction and sequencing: Total RNA was isolated from cells using Trizol reagent (Invitrogen), according to the manufacturer protocol. cDNA libraries were prepared using the TruSeq Stranded mRNA LT Sample Prep kits (Illumina), according to the manufacturer guidelines, TruSeq Stranded mRNA Sample Preparation Guide, Part # 15031047 Rev. E. In brief, after being purified from total RNA, the mRNA was randomly fragmented and reverse transcribed into cDNA. Adapters were ligated onto both ends of the cDNA fragments to amplify them using PCR. After amplification, the fragments with insert size between 200 bp and 400 bp were selected for paired-end sequencing. The sequencing process was performed on an Illumina NovaSeq 6000 instrument, using the NovaSeq 6000 S4 Reagent kits, and according to the TruSeq Stranded mRNA Sample Preparation Guide, Part # 15031047 Rev. E.

Expression profiling: The quality of reads was determined by using FastQC[1] version 0.11.7. Trimmomatic[2] version 0.38 was used to filter out low-quality reads by performing the following

trimming tasks: cutting of adapter sequences, cutting of bases off the ends of reads if below a quality score of 3, cutting of windows of four bases if the average quality fell below a quality score of 15, and, finally, dropping reads shorter than 36 bp. HISAT2 version 2.1.0[3], a spliced alignment program, was used to align the trimmed reads to the reference genome (Mouse GRCm38/mm10). The expression levels of known genes were determined using StringTie version 1.3.4d[4], with the mm10 reference annotation as the assembly guide. Raw read counts where normalized using the median ratio normalization method[5], and the resulting normalized count tables were fed to GFOLD version 1.1.4[6], to perform gene expression analysis, setting the significant cut-off for fold-change to the default 0.01. Accession to RNAseq data: http://www.ebi.ac.uk/arrayexpress/experiments/E-MTAB-9231.

Gene-set enrichment analysis: Gene-set enrichment analysis was performed using GSEA Pre-ranked version 4.0.3[7,8]. As input, we used the list of *gfold* values calculated by GFOLD, which can be considered as a sort of reliable log2 fold-change, ranked by *gfold* value, and the list of gene-sets from the Molecular Signatures Database[7,9] (MSigDB version 7.0) belonging to the collection of canonical pathways (CPs) of the Curated Gene Sets (C2). To avoid unrankable values in the input list, genes with *gfold*=0, which GFOLD classifies as nondifferentially expressed, were removed from the list before running GSEA. The number of permutations to assess the statistical significance of the enrichment score, done by gene set, was set to 1000. To identify a subset of 'core genes' potentially responsible for most of the features associated with different phenotypes, we compared the lists of genes from the leading edge of enriched gene-sets coming from each CP sub-collection (Biocarta, Kegg, Pid, Reactome, Naba), and selected those shared by at least four out of five of these sub-collections.

Cell culture

The triple-negative murine 4T1 Dual LN P3 (referred to as 4T1; ATCC: #CRL-2539) breast-cancer cell line was grown in RPMI 1640 medium (Euroclone) supplemented with 10% (v/v) fetal bovine serum (FBS; Invitrogen), 2 mM L-glutamine (Invitrogen), and 1% (v/v) antibiotics (10000 U/mL penicillin, 10 mg/mL streptomycin [Invitrogen]) (Pen/Strep). J774A.1 murine macrophages (referred to as J774 cells; ATCC: #TIB-67) were grown in high-glucose Dulbecco's modified Eagle's medium (DMEM; Euroclone) supplemented with 10% (v/v) FBS, 2 mM L-glutamine, and 1% (v/v) antibiotics (Pen/Strep). RAW-264.7 murine macrophages (referred to as RAW-264 cells; ATCC: #TIB-71) were grown in high-glucose DMEM supplemented with 10% (v/v) FBS, 4 mM L-glutamine, and 1% (v/v)

antibiotics (Pen/Strep). The cells were grown at 37 °C in a humidified atmosphere of 95% air, 5% CO_2 (v/v).

Primary murine TNBC cells from tumors generated by MMTV–Prune-1/Wnt1 and MMTV–Wnt1 mice were obtained (2 months from tumor onset). MMTV–Prune-1/Wnt1 and MMTV–Wnt1 mice were grown in high-glucose DMEM (Euroclone) supplemented with 20% (v/v) FBS, 2 mM L-glutamine, 1% non-essential amino acid solution (M7145; Sigma-Aldrich/Merck) and 1% (v/v) antibiotics (Pen/Strep).

Generation of 4T1 stable clones and transient transfection in MMTV-Prune-1/Wnt1 cells

A construct encoding a fusion protein containing three Flag epitopes (Flag sequence: DYKDDDDK) upstream the Prune-1 protein was generated in the pcDNA3.1 Hygro vector (H-Prune-1-3X-Flag-Hygro plasmid). This construct was transfected to 4T1 cells to generate clones overexpressing the Flag-h-Prune-1 fusion protein (referred to as Prune-1–overexpressing 4T1 clones). The pcDNA3.1 Hygro vector was also transfected to generate control clones.

To generate 4T1 clones in which mouse prune endogenous expression was down-regulated (referred to as Prune-1–silenced 4T1 clones), the cells were transfected with a plasmid containing sh-Prune (Open Biosystems). Similarly, control clones were generated by transfecting the 4T1 cells with the empty vector plasmid.

One day before transfection, the 4T1 cells were plated in 60-mm dishes. At the time of transfection, the cell confluence was 50%, and the cells were transfected with 2.4 µg each vector, using TransIT-LT1 Transfection Reagent (#MIR2300; Mirus Bio LLC), according to the manufacturer instructions. Forty-eight hours after transfection, the cells were cultured under the selection pressure of 250 µg/mL hygromycin B (Invitrogen) for the pcDNA3.1 Hygro constructs. After the selection, about 20 clones for each transfected construct were picked and the expression levels of human and mouse prune proteins were evaluated.

For transient transfection, the day before transfection, the MMTV-Prune-1/Wnt1 cells were plated in 60-mm dishes. At the time of transfection, the cell confluence was 50%, and the cells were transfected with 1 µg IL-17shRNA Pllasmid (m) #sc-146204-SH, using TransIT-LT1 Transfection Reagent (#MIR2300; Mirus Bio LLC), according to the manufacturer instructions. After 24 hours, 1 $\times 10^6$ MMTV-Prune-1/Wnt1 transfected cells were resuspended in 10 mL complete DMEM medium, plated into 10-cm-diameter plates, and grown at 37 °C for 24 h. Then the conditioned media were collected.

Genotyping

Mice had their tail docked (0.25 inches) for DNA analysis from 8-10 days of age. Tail samples were digested with 100 mM Tris-HCl, pH 8.8, 5 mM EDTA, pH 8.0, 0.2% SDS, 200 mM NaCl and 100 μg/mL proteinase K at 55 °C overnight. Samples were then vortexed and centrifuged for 5 min (14,000 rpm, at room temperature) to pellet debris. PCR analyses for MMTV–Prune-1 and MMTV–Wnt1 detection were performed using PCR (GeneAmp System 9700; Applied Biosystem) under the following conditions: 94 °C for 5 min; 94 °C for 30 s, 55 °C for 45 s, 72 °C for 1 min (×45 cycles); 72 °C for 10 min.

Oligonucleotide sequences for MMTV-Prune-1: Forward, GTGAGACAAGTGGTTTCCTGA; Reverse, GCTCGATGGGTCGATGGTCT.

Oligonucleotide sequences for MMTV-Wnt1 (Liu et al., 2008): Forward, GGACTTGCTTCTCTTCTCATAGCC; Reverse, CCACACAGGCATAGAGTGTCTGC.

Immunoblotting

The cells were washed in cold phosphate-buffered saline (PBS) and lysed in cell lysis buffer (20 mM sodium phosphate, pH 7.4, 150 mM NaCl, 10% [v/v] glycerol, 1% [w/v] nadeoxycholate, 1% [v/v] Triton X-100) supplemented with protease inhibitors (Roche, Basel, Switzerland). Cell lysates were cleared by centrifugation at 16,200× *g* for 10 min at room temperature, and the supernatants were removed and assayed for protein concentrations (Protein Assay Dye Reagent; Bio-Rad). Cell lysates (50 μg protein lysate) were separated on SDS-PAGE gels of different percentages, depending on the molecular weights of the proteins of interest. The proteins were then electrophoretically transferred to PVDF membranes (Millipore). After 1 h in blocking solution with 5% (w/v) dry milk fat in PBS, or 5% (w/v) bovine serum albumin (Sigma-Aldrich) in Tris-buffered saline (both of which contained 0.02% [v/v] Tween-20), the membranes were incubated with the primary antibody overnight at 4 °C, and then with the secondary antibodies for 1 h at room temperature. Primary and secondary mouse or rabbit horseradish-peroxidase-conjugated antibodies (NC 27606; ImmunoReagents, Inc) were diluted in 5% (w/v) bovine serum albumin in TBS-Tween or in 5% (w/v) milk fat in PBS-Tween, according to the manufacturer instructions. The protein bands were visualized with a chemiluminescence detection system (Pierce-Thermo Fisher Scientific Inc., IL, USA). Western blotting was performed in triplicate. The densitometry analysis was carried out using the ImageJ software program. The peak areas of the bands were measured on the densitometry plots, and the percentages were calculated. Then, the density areas of the peaks were normalized with those of the loading controls, and the ratios for the corresponding controls (*e.g.*, empty vector) are presented as fold-changes.

Immunohistochemistry of paraffin-embedded tissues

Paraffin sections (thickness, 3 μm) of the tumor specimens were deparaffinized (Bioclear, 06-1782D; Bio-Optica) for 30 min, rehydrated in 100%, 90%, then 70% ethanol, and washed with PBS and then PBS containing 0.02% Triton-X 100 (215680010; Acros Organics). After incubation in pre-warmed target retrieval solution (S170084; Dako) at 97 °C for 45 min, the sections were washed with PBS and placed in a solution of absolute methanol and 0.3% hydrogen peroxide for 15 min. The tissue sections were then blocked with Antibody Diluent Background Reducing (S302281; Dako) for 1 h at room temperature, and then incubated with the primary antibodies overnight at 4 °C in a humidified chamber. Tissue sections were washed in PBS and incubated with labeled streptavidin biotin LSAB mouse and rabbit reagents (K0672; Dako). Detection was with the Liquid DAB Substrate Chromogen System (K3468, Dako). All of the slides were counterstained with Gill's hematoxylin (Bio-Optica). The slides were then washed, dehydrated with 70%, 90%, and then 100% ethanol, and mounted with cover slips using Eukitt (09-00250; Bio-Optica). Micrographs were taken with a high-definition digital microscope camera (ICC50 HD; Leica) or with Mantra Quantitative Pathology Workstation, using the 40× objective. The quantification was performed by using inForm image analysis software.

The detection of macro-metastases in lungs derived from MMTV–Prune-1/Wnt1 and MMTV–Wnt1 cells was performed following staining with Bouin's fixative (25% of 37% formaldehyde solution, 70% picric acid, 5% acetic acid) for 24 h.

Immunofluorescence of paraffin-embedded tissue sections

Paraffin sections of the tumor specimens (thickness, 3 μm) were deparaffinised and rehydrated by immersing the slides in Xylene Substitute (A5597; Sigma), as three washes for 5 min each, then serially in 100%, 95%, 70%, 50%, and 30% ethanol (two washes for 10 min, for each), and deionised water (two washes, for 5 min each). The washes were then followed by PBS, PBS containing 0.02% Triton-X 100 (215680010; Acros Organics), and PBS (two washes for 5 min, for each). For antigen retrieval, the slides were immersed in boiling 10 mM sodium citrate buffer (pH 6.0) using a microwave oven, and then maintained at sub-boiling temperature for 10 min. The slides were left to cool at room temperature for 30 min. The sections were then washed by immersion in distilled water for 5 min. To block endogenous peroxidase activity, the tissue sections were placed in a solution with 3.0% hydrogen peroxide in methanol for 15 min. Then, to decrease the nonspecific background fluorescence, the tissues were digested by treating them with a solution containing 0.2% trypsin (T2600000; Sigma-Aldrich) and 0.001% $CaCl_2$ for 10 min at 37 °C, in a humidified chamber. The slides were then washed in PBS, PBS containing 0.02% Triton-X 100 (215680010; Acros Organics), and PBS (two washes for 5 min, for each). The tissue sections were blocked with 6% bovine serum

albumin (A9418; Sigma), 5% fetal bovine serum (ECS0180L; Euroclone), and 20 mM $MgCl_2$ in PBS containing 0.02% Triton-X 100, for 1 h at room temperature, and incubated overnight with the primary antibodies at 4 °C in a humidified chamber. Tissue sections were washed in PBS and PBS containing 0.02% Triton-X100, and incubated with anti-mouse Alexa Fluor 488 (ab150113; Abcam), anti-rabbit Alexafluor 488 (150077; Abcam) and antirabbit Alexa Fluor 546 (#A10040; ThermoFisher), as the secondary antibodies. DNA was stained with DRAQ5 (#62254; ThermoFisher). The slides were then washed, dehydrated with 70%, 90%, and 100% ethanol, and mounted with cover slips using 50% glycerol (G5150; Sigma-Aldrich). Confocal microscopy was carried out using Mantra Quantitative Pathology Workstation, using the 40× objective or a laser scanning confocal microscope Leica TCS SP5, using the 63× oil immersion objective. The quantification was performed by using inForm image analysis software.

Antibodies used

The following antibodies were used in this study: anti-Flag (1:5000; Sigma-Aldrich); anti-FLAG (1:200; TA100023; Origene); anti-phospho-p44/42 MAPK (ERK1/2) (Thr 202/Tyr 204) (1:500; Cell Signaling Technology); anti-ERK1/2 (1:500; Santa Cruz Biotechnology); anti-phospho-NF-κB p65 (Ser 311) (1:200; Santa Cruz Biotechnology); anti-NF-κB p65 (1:3000; Abcam); anti-STAT3 (1:5000; Abcam); anti-phospho-STAT3 (Tyr 705) (1:1000; Abcam); anti-phospho-STAT3 (Ser 727) (1:1000; Abcam); anti-Prune-1 (1:500; Abcam); anti-phospho-Ser467-Smad2 (1:1000; Cell Signaling); anti-PTEN (1:1000; 9552, Cell Signaling); anti-phospho-Ser473-Akt (1:500; Cell Signaling); anti-N-cadherin (1:1000; Cell Signaling) anti-E-cadherin (1:500; Transduction Laboratories:); anti-GSK (1:500, Transduction Laboratories); anti-Ser9/21-GSK (1:200; Cell Signaling); anti-FAK (1:1000; Abcam); anti-phospho-Y397-FAK (Abcam); anti-CD68 (1:200; Abcam); anti-CD163 (1:200; SantaCruz); anti-activated β-catenin (1:500; Millipore); anti- β-catenin (1:2000; Transduction Laboratories); anti-Wnt3a (1:500; Abcam); anti-cyclin D1 (1:5000; Abcam); and anti-Her2 (1:200; Cell Signaling); Anti-Vimentin (1:200; ab137321; Abcam) The following in-house antibodies were produced: rabbit polyclonal anti-Prune-1 (C45; 1:500; (Ferrucci et al., 2018)); rabbit polyclonal anti-phospho-NME1 (pS120-pS122-pS125; 1:500; (Garzia et al., 2008). These antibodies were detected using horseradish peroxidase–conjugated anti-mouse (1:5000; Amersham) and anti-rabbit (1:3000; Amersham). An anti-β-actin (1:5000; Sigma-Aldrich) and anti-α-tubulin (1:3000; Abcam) antibodies were used as controls for equal loading.

Quantitative real-time polymerase chain reaction (RT-PCR)

Total RNA was isolated from cells using Trizol reagent (Invitrogen), according to the manufacturer protocol. cDNA was synthesized using random hexamers with iScript cDNA synthesis kits (Bio-Rad), according to the manufacturer protocols. After digestion with DNAse RNAse-free, 2 μg total RNA in 20 μL was used in each reaction. qRT-PCR was performed using the SYBR Green PCR Master Mix (Applied Biosystems) and a sequence detection system (model 7900HT, Applied Biosystems), according to the manufacturer protocols. The primers were designed with the Primer Express 2.1 program (Applied Biosystems). All qRT-PCRs were performed in duplicate, with 50 ng ss-cDNA used in each 10-μL reaction. β-Actin mRNA was used to normalize mRNA concentrations. For statistical analysis of gene expression data, the relative expression $2^{-\Delta Ct}$ ±standard deviation and the mean fold change = $2^{-(\text{average } \Delta\Delta Ct)}$ ±standard deviation were calculated using mean differences in ΔCt between the genes and the internal control. The ΔCt was calculated using the differences in the mean Ct between the genes and the internal control.

Generation of *MMTV*–Prune-1-Flag construct

The pMSG vector (Pharmacia Biotech Sevrage, Uppsala, Sweden) contains the mouse Mammary tumor virus long terminal repeat (MMTV-LTR) upstream of a polylinker. The human Prune-1 cDNA containing the complete protein coding region with the FLAG tag fused in-frame at the carboxyl-terminus was cloned into polylinker sites of pMSG in a sense orientation relative to the MMTV-LTR, and downstream of the SV-40 early promoter. The resultant construct was designated pMSG-MMTV–Prune-1-FLAG.

To generate MMTV–Prune-1, transgenic animals in an FVB background were used, with pronuclear injections of the fertilized oocytes, and embryo transfer to the pseudopregnant females. Male MMTV–Wnt1 mice (FVB) were crossed with MMTV–Prune-1 (FVB) to generate the double transgenic MMTV–Prune-1/Wnt1 mouse model.

Tail vein injection of MMTV–Prune-1/Wnt1 cells in FVB mice

MMTV–Prune-1/Wnt1 cells (1×10^5) were injected (via tail vein) in immunocompetent syngenic (strain FVB) mice (n=8). At 14 days from cell injection, the mice were grouped according to their weight and AA7.1 (60 mg/kg/day, IP) or PBS (as vehicle negative control) were administered daily. At 14 days from treatment start (*i.e.,* n.28 days from TNBC cell injection), the mice were injected with a fluorescent imaging probe (XenoLight RediJect 2-DG-750; Perkin Elmer) that targets cells with high metabolic activity in terms of glucose uptake, for *ex-vivo* targeting of tumorigenic cells.

Orthotopic syngenic mice models of 4T1 cells in BalbC mice

Five-week-old female immunocompetent BalbC mice were anesthetized with ketamine/ xylazine (87.5 mg/kg, 12.5 mg/kg, respectively). They were then implanted in the VIII right-side mammary gland with 2.5 $\times 10^5$ 4T1 cells of Prune-1–silenced, extracellular vesicle clones, or 4T1-LUC cells (stably expressing the firefly luciferase gene through lentiviral particles; RediFect Red-Fluc-Puromycin; CLS960002; Perkin Elmer, Waltham, MA, USA)). The mice were imaged weekly and tumor growth was evaluated by bioluminescence acquisition using an imaging system (IVIS 3D Illumina; Xenogen/ Caliper), as described by Asadzedeh et al. (79). Briefly, for the acquisitions, the mice were anesthetized by inhalational of isofluorane, and D-luciferin (15 mg/mL stock; 122799; PerkinElmer) was injected intraperitoneally (100 µL per 10 g body weight). To quantify the bioluminescent imaging (BLI), the integrated fluxes of photons (ph/s) within each area of interest were determined using the Living Images Software Package 3.2 (Xenogen-Perkin Elmer).

For treatment with AA7.1 *in-vivo*, starting 14 days from tumor implantation (i.e., once tumors were established), the mice were grouped according to their bioluminescence values and injected intraperitoneally with AA7.1 60 mg/kg daily, or with PBS as the vehicle control. Tumor growth was monitored every 7 days by BLI aquisition, with means ±standard error of the photon integrated fluxes calculated for each experimental point. At the end of the experiments, the primary tumors were dissected out and embedded in paraffin for immunohistochemistry (IHC) analysis.

Proteomic analysis performed on extracellular vesicles

The extracellular vesicles (EVs) isolated from culture media from MMTV–Prune-1/Wnt1 and MMTV–Wnt1 cells (following methodology described previously (Thery et al., 2006)) were prepared for mass spectrometry as follows. The EVs were lysed by addition of 0.4% sodium dodecyl sulfate (SDS). After a brief period of incubation, the samples were diluted to drop the SDS concentration to 0.1% SDS. To purify the samples, we used the short SDS-PAGE method (to remove the disturbing components from the proteins upon subsequent protein staining). Briefly, we ran only in the stacking part of the polyacrylamide gel. Once the SDS-PAGE run was finished, the proteins were visualised by Coomassie blue and cut out of the gel to perform in-gel digestion. These samples were analysed using a mass spectrometer (LTQ-Orbitrap VELOS), with a 60-min gradient run. The data obtained by the mass spectrometry were presented to the Swiss-Prot '*Mus musculus*' database. To identify MS/MS spectra and further proteins, we used the Mascot database search engine. The identification was carried out with confidence settings of 99%. The threshold score itself was set by the 99% confidence interval setting, the size of the database, tolerances set to the measured masses to the precursor and the fragments. After the database search the results were stored in the ms_lims system (Helsens et al., 2010).

Statistical analysis

Data representative of three independent experiments were analyzed using unpaired two-tailed t-tests (Student's t-tests). For all of the Figures, error bars represent standard deviation (SD) of the mean, and $p \leq 0.05$ was considered significant.

***In-vivo* human experiments**

Patients and specimens: From 2003 to 2010, 138 patients who underwent a mastectomy, quadrantectomy or metastectomy at the 'Giovanni Pascale' National Cancer Institute of Naples, Italy, were enrolled into this study. In our institution, the proportion of tumors classified as TNBC is 15% to 19% of the total number of breast cancer surgeries. All cases of TNBC and nonTNBC samples were reviewed according to WHO classification criteria, using standard tissue sections and appropriate IHC slides. Medical records for all of the cases of TNBC and nonTNBC samples were reviewed for clinical information, including histologic parameters that were determined from the hematoxylin-eosin stained slides. The following clinical and pathological parameters were evaluated for each tumor included in the study: patient age at initial diagnosis, tumor size, histologic subtype, grade, nodal status, tumor recurrence or distant metastasis. In addition, all of the specimens were characterized for all routine diagnostic immunophenotypic parameters.

Tissue microarray building: The 138 patients were used for tissue microarray building, using the most representative areas from each single case. All of the tumors and controls were reviewed by two experienced pathologists (MDB/GB). Discrepancies between two pathologists from the same case were resolved in a joint analysis of the cases.

IHC analysis: IHC staining was carried out on slides from formalin-fixed, paraffin embedded tissues, corresponding to the TNBC tissue microarrays, to evaluate the expression of Prune-1, ER, PgR, c-Erb B2, Ki67, pERK, NF-κB-p65-Ser311, CD68, and CD163 markers. Slides were incubated with antibodies against routinely diagnostic markers: mouse anti-human ERα (1:35; DAKO), mouse anti-human PR (1:50; DAKO), rabbit anti-c-Erb B2 (1:300; DAKO), and mouse anti-human Ki67 (1:75; DAKO), and with primary antibodies listed as follow: rabbit anti-prune (1:200; made in home) (Ferrucci et al., 2018), rabbit anti- ERK1/2 (phospho Thr202/Tyr204) (1:500 dilution; Cell Signaling), rabbit anti-NFkB p65 (phospho S311) (1:400; Abcam), mouse anti-CD68 (prediluited, Ventana, Roche), mouse anti-CD163 (1:75; Novocastra). The negative controls omitted the primary antibody. Detection was achieved with a Liquid DAB + Substrate Chromogen System (Dako).

Sections were counterstained with hematoxylin and mounted. Results were interpreted using light microscopy.

Evaluation of IHC: Antigen expression was evaluated independently by two pathologists using light microscopy. Observers were unaware of the clinical outcome. For each sample, at least five fields (inside the tumor and in the area showing tumor invasion; 400×400) and >500 cells were analyzed. Using a semiquantitative scoring system microscopically, and referring to each antigen scoring method in other studies, an observer evaluated the intensity, extent, and subcellular distribution of Prune-1, ER, PgR, c-Erb B2, Ki67, ERK 1/2 (phospho Thr202/Tyr204), NF-kB p65 Ser311, CD68, and CD163 markers. The cut-off used to distinguish 'positive' from 'negative' cases was ≥1% ER/PR positive tumor cells. IHC analyses of c-erbB-2 expression described the intensity and staining pattern of the tumor cells. Only membrane staining intensity and pattern were evaluated using the 0 to 3+ score, as illustrated in the HercepTest kit scoring guidelines. The FDA-recognized test, the Herceptest (DAKO), describes four categories: no staining, or weak staining in <10% of the tumor cells (0); weak staining in part of the membrane in >10% of the tumor cells (1+); complete staining of the membrane with weak or moderate intensity in >10% of the neoplastic cells (2+); and strong staining in >10% (3+). Scores of 0 or 1+ were considered negative for *HER-2/neu* -expression, 2+ was uncertain, and 3+ was positive. Cases 2 + undergo FISH analysis (Collina et al., 2016).

The proliferative index Ki67 was defined as the proportion (%) of immunoreactive tumor cells out of the total number of cells. The proportion of positive cells per case was scored according to two different groups: group 1: ≤20% (low proliferative activity); group 2: >20% (high proliferative activity).

There are no standardized criteria for Prune-1, ERK (phospho Thr202/Tyr204), NF-κB p65 (phospho Ser311), CD68, and CD163 marker staining evaluation, so the median of the percentage positivity for each marker was used as the cut-off. We schematized the score evaluation as follows: for cytoplasmic prune staining, we used a combo score considering both intensity of reaction (high (3)/ moderate (2)/ low (1)/ absent (0)) and cell percentage positivity (0=negative/ 1=>1%<30%/ 2=>30<60/ 3=>60%) and considered high expression cases when the combo value was >5; also for nuclear phospo-p65 (Ser311), we used a combo score considering both an intensity score (based on a four-point system: high (3)/ moderate (2)/ low (1)/ absent (0) staining) and cell percentage positivity (0=negative/ 1=<50%/ 2=≥50%), and considered high expression cases when the combo value was >2 (median value); for nuclear phospho-ERK we considered cell percentage positivity as low when it was ≤20%, high when >20%.

To characterize microenvironment markers, we selected 32 cases in our casuistry (16 high and 16 low for Prune expression) and we performed the analysis on whole sections. We used these following scores: for cytoplasmic CD68, as low when it was <30%, high when ≥30%, for cytoplasmic CD163, as low when it was <30%, high when ≥30%.

Statistical analysis: Student's *t*-tests and Pearson χ^2 tests were used to determine whether a relationship existed between the categorical variables included in the study. The level of significance was defined as P <0.05. All of the statistical analyses were carried out using the Statistical Package for Social Science v. 20 software (SPSS Inc., Chicago, IL, USA).

Supplemental References

Collina, F., Cerrone, M., Peluso, V., Laurentiis, M. D., Caputo, R., Cecio, R. D., Liguori, G., Botti, G., Cantile, M. and Bonito, M. D. (2016). Downregulation of androgen receptor is strongly associated with diabetes in triple negative breast cancer patients. *Am J Transl Res,* 8**,** 3530-9.

Ferrucci, V., De Antonellis, P., Pennino, F. P., Asadzadeh, F., Virgilio, A., Montanaro, D., Galeone, A., Boffa, I., Pisano, I., Scognamiglio, I., et al. (2018). Metastatic group 3 medulloblastoma is driven by PRUNE1 targeting NME1-TGF-beta-OTX2-SNAIL via PTEN inhibition. *Brain,* 141**,** 1300-1319.

Garzia, L., D'angelo, A., Amoresano, A., Knauer, S. K., Cirulli, C., Campanella, C., Stauber, R. H., Steegborn, C., Iolascon, A. and Zollo, M. (2008). Phosphorylation of nm23-H1 by CKI induces its complex formation with h-prune and promotes cell motility. *Oncogene,* 27**,** 1853-64.

Helsens, K., Colaert, N., Barsnes, H., Muth, T., Flikka, K., Staes, A., Timmerman, E., Wortelkamp, S., Sickmann, A., Vandekerckhove, J., et al. (2010). ms_lims, a simple yet powerful open source laboratory information management system for MS-driven proteomics. *Proteomics,* 10**,** 1261-4.

Liu, B., Yu, H. M., Huang, J. and Hsu, W. (2008). Co-opted JNK/SAPK signaling in Wnt/beta-catenin-induced tumorigenesis. *Neoplasia,* 10**,** 1004-13.

Thery, C., Amigorena, S., Raposo, G. and Clayton, A. (2006). Isolation and characterization of exosomes from cell culture supernatants and biological fluids. *Curr Protoc Cell Biol,* Chapter 3**,** Unit 3 22.
